# Improving generalizability of drug–target binding prediction by pre-trained multi-view molecular representations

**DOI:** 10.1093/bioinformatics/btaf002

**Published:** 2025-01-07

**Authors:** Xike Ouyang, Yannuo Feng, Chen Cui, Yunhe Li, Li Zhang, Han Wang

**Affiliations:** School of Information Science and Technology, Institute of Computational Biology, Northeast Normal University, Changchun, Jilin 130117, China; School of Information Science and Technology, Institute of Computational Biology, Northeast Normal University, Changchun, Jilin 130117, China; School of Computer Science and Engineering, Changchun University of Technology, Changchun, Jilin 130051, China; School of Information Science and Technology, Institute of Computational Biology, Northeast Normal University, Changchun, Jilin 130117, China; School of Computer Science and Engineering, Changchun University of Technology, Changchun, Jilin 130051, China; School of Information Science and Technology, Institute of Computational Biology, Northeast Normal University, Changchun, Jilin 130117, China

## Abstract

**Motivation:**

Most drugs start on their journey inside the body by binding the right target proteins. This is the reason that numerous efforts have been devoted to predicting the drug–target binding during drug development. However, the inherent diversity among molecular properties, coupled with limited training data availability, poses challenges to the accuracy and generalizability of these methods beyond their training domain.

**Results:**

In this work, we proposed a neural networks construction for high accurate and generalizable drug–target binding prediction, named Pre-trained Multi-view Molecular Representations (PMMR). The method uses pre-trained models to transfer representations of target proteins and drugs to the domain of drug–target binding prediction, mitigating the issue of poor generalizability stemming from limited data. Then, two typical representations of drug molecules, Graphs and SMILES strings, are learned respectively by a Graph Neural Network and a Transformer to achieve complementarity between local and global features. PMMR was evaluated on drug–target affinity and interaction benchmark datasets, and it derived preponderant performance contrast to peer methods, especially generalizability in cold-start scenarios. Furthermore, our state-of-the-art method was indicated to have the potential for drug discovery by a case study of cyclin-dependent kinase 2.

**Availability and implementation:**

https://github.com/NENUBioCompute/PMMR.

## 1 Introduction

In the process of new drug discovery, the identification of drug–target binding is a highly complex step, critical for uncovering drug candidates, understanding the mechanism of action of drug molecules, conducting multi-targeting studies of drug molecules, and exploring drug repurposing (Malathi and Ramaiah 2018). Compared with the wet experiments for drug screening, computational methods are widely spread for the reason of fast, convenient and high-throughput characteristics (Schneider 2018, Dara *et al.* 2022).

Molecular docking (Forli *et al.* 2016, Yan *et al.* 2017) is a classical computational approach for drug–target binding discovery. It can simulate the binding conformation of drug–target and has good physical interpretability. However, it requires huge computing resources and high-quality complex structures of drug–targets are scarce, which brings challenges to large-scale screening.

Another type of drug–target binding prediction approach prefers to utilize machine learning methods for modeling and prediction, especially deep learning in recent decade. When the model input includes only molecular descriptors of drugs [such as SMILES strings (Weininger 1988)] and amino acid sequences of target proteins, drug–target binding prediction can be performed rapidly (Abbasi *et al.* 2020, Yuan *et al.* 2022, Zhang *et al.* 2022, Gim *et al.* 2023). However, relying solely on the SMILES representations of a drugs may not fully capture the unique features of its molecular structure, potentially reducing the predictive performance of the model (Liu *et al.* 2024). Recently, graph neural networks (GNNs) have been widely used due to their strong ability to effectively capture complex structures in non-Euclidean spatial data (Jin *et al.* 2021, Li *et al.* 2022, Bi *et al.* 2023, Hua *et al.* 2023a,b, Zhang *et al.* 2023, Peng *et al.* 2024). MolTrans (Huang *et al.* 2021) and MgraphDTA (Yang *et al.* 2022) utilized graph representations of drugs and showed good prediction performance on some public datasets. Although these proposed methods show good prediction performance on various datasets, their performance in handling unseen inputs leaves much room for improvement due to the limited availability and diversity of data (Singh *et al.* 2023). Furthermore, the improvement of model accuracy depends not only on the design of the model but also on the richness of the features. Current methods have not fully exploited the complementarity between different view features, which is crucial for prediction.

In this study, we proposed a drug–target binding prediction method called Pre-trained Multi-view Molecular Representations (PMMR). One way to address the limitations of the dataset is by transferring target protein and drug representations from language models (LMs) to drug–target binding prediction. Therefore, PMMR first uses a protein language model (ESM-2) and a chemical language model (ChemBERTa-2) to extract pre-trained features of protein sequences and drug SMILES respectively to alleviate the problem of poor generalization performance caused by insufficient data. In addition, PMMR utilizes a drug decoder to fuse the graph features extracted by GNN with the pre-trained SMILES features fine-tuned by Transformer, thereby achieving the complementarity between local and global features. The performance of PMMR is demonstrated through tests on various datasets and cold-start scenarios. Finally, it offers new insights into drug discovery through a case study on Cyclin-dependent kinase 2 (CDK2).

## 2 Materials and methods

### 2.1 Benchmarks overview

#### 2.1.1 DTA benchmarks

Evaluation of regression performance of PMMR on three benchmark affinity datasets: TDC-DG (Huang *et al.* 2021), Davis (Davis *et al.* 2011), PDBbind (Liu *et al.* 2017). TDC-DG train set contains binding affinity $\left({IC}_{50}\right)$ data from interactions patented between 2013 and 2018, with test set drawn from interactions panteted in 2019 and 2021. Davis dataset contains pairwise interactions between 442 kinase proteins and 68 drugs, with affinity values evaluated by experimentally measured $K_{d}$ value. The structural dataset PDBbind includes a collection of experimentally verified protein-ligand binding affinity expressed with $-\log K_{i}$, $-\log K_{d}$ or $-\log{IC}_{50}$ from the Protein Data Bank (PDB) database (Burley *et al.* 2019). Here, we use the 2016 version of the training set and test set from the PDB database. These datasets have provided protein PDB files, pocket PDB files and ligand SDF files, etc. Here, we use the protein sequence data collected from PDB files and the SMILES data collected from SDF files based on DeepDTAF (Wang *et al.* 2021) as the original dataset. During the conversion of SMILES data into 2D graph representations, certain SMILES strings could not be parsed due to specification issues. Therefore, the generated training dataset contains 7512 affinity data, and the test dataset contains 207 affinity data, details are shown in Table 1.

**Table 1.** Details of benchmark datasets.

Dataset	Drugs	Targets	Training	Validation	Test
Davis	68	442	24 044	3006	3006
PDBbind	6487	5266	7512		207
TDC-DG	140 469	476	182 905		48 992
BindingDB	7165	1254	12 657		13 272

#### 2.1.2 DTI benchmarks

We test the classification performance of PMMR on the drug–target interaction dataset BindingDB (Liu *et al.* 2007). It consists of pairs of drugs and targets with experimentally determined dissociation constants ($K_{d}$). According to the literature (Singh *et al.* 2023), we treat pairs with $K_{d}$ < 30 as positive DTIs, while larger $K_{d}$ values are negative.

### 2.2 Model architecture

**Figure 1.**
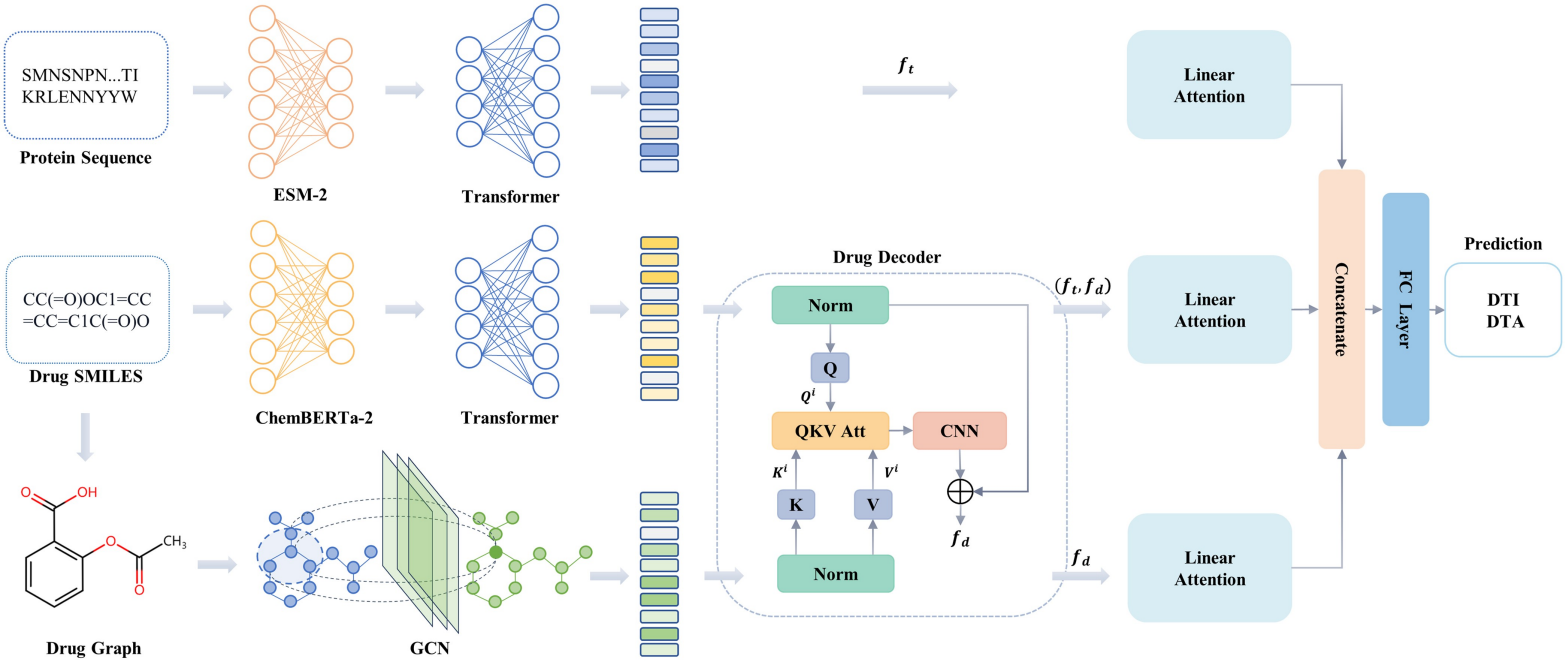
The overall architecture of PMMR. Firstly, PMMR employ pre-trained models to extract features from protein sequences and drug SMILES. Subsequently, Transformer fine-tunes these extracted features. Then, GCN is then utilized to extract features from drug molecular graphs. The SMILES features and molecular graph features are inputted into the drug decoder for decoding, yielding an overall drug feature representation. In addition, protein features, drug features, and the concatenate features between drugs and proteins are fed into a linear attention mechanism to extract effective features individually. Finally, Concatenation is performed on the extracted features for affinity and interaction prediction.

The overall architecture diagram of PMMR is shown in Fig. 1. First, pre-trained features of proteins and drugs were extracted using ESM-2 and ChemBERTa-2, respectively. Subsequently, Transformer was employed to fine-tune these pre-trained features. GCN was used to extract graph features of drugs, which were then fused with SMILES features using a drug decoder to obtain the final drug representation. Next, input the protein features ($f_{t}$), drug features ($f_{d}$), and spliced protein and drug features ($f_{t},\;f_{d}$) into the linear attention module respectively. Finally, these effective feature representations are concatenated, and a fully connected layer is utilized for prediction.

#### 2.2.1 Target protein encoding

For the target protein sequences, we use the protein language model ESM2-35M (Lin *et al.* 2023) to generate initial features. ESM-2 is a transformer-based protein language model that learns the interaction patterns between amino acids in protein sequence. This allows ESM-2 to capture protein evolutionary information. The initial features extracted by ESM-2 are represented as follows:

(1) $$e_{t}\;=\;\mathrm{ESM}\left(P_{s}\right)W_{p}\in R^{n\times d_{t}},$$

where *n* is sequence length, $d_{t}$ is the dimension of the hidden layer in the pre-training model and $W_{p}$ is a trainable weight matrix.

In order to adapt the pre-trained features to downstream tasks, we use a transformer (Vaswani *et al.* 2017) to fine-tune pre-trained features. At the same time, due to the inconsistency in protein sequence lengths, we take the largest sequence length in each batch as the unified length. The resulting features are expressed as follows:

(2) $$f_{t}\;=\;\mathrm{transformer}\left(e_{t}\right).$$

#### 2.2.2 Drug molecular encoding

In the drug encoding section, the chemical language model ChemBERTa-2 (MLM) (Ahmad *et al.* 2022) is used to obtain the pre-trained features of drug SMILES strings. ChemBERTa-2 is a BERT-like transformer model that learns molecular fingerprints through semi-supervised pre-training of the language model. ChemBERTa-2 employs masked-language modeling (MLM) and multi-task regression (MTR) over a large corpus of 77 million SMILES strings, a well-known text representation of molecules. Given the SMILES input, the generated features as follows:

(3) $$c_{\mathrm{smi}}\;=\;\mathrm{ChemBERTa}\left(X_{\mathrm{smi}}\right)W_{d}\in R^{n\times d_{c}}.$$

In order to ensure the length consistency of SMILES in each batch, we also take the maximum SMILES length as the unified length. Then, transformer is used to fine-tune pre-trained SMILES features.

(4) $$f_{s}\;=\;\mathrm{transformer}\left(c_{\mathrm{smi}}\right).$$

Instead, we use RDKit (Landrum 2013) to convert SMILES to graphs. Each node is a multi-dimensional binary feature vector expressing five pieces of information: the atom symbol, the number of adjacent atoms, the number of adjacent hydrogens, the implicit value of the atom, and whether the atom is in an aromatic structure (Nguyen *et al.* 2021). To ensure consistent numbers of graph nodes, we adopt the maximum number of graph nodes in each batch as the standard. Then, we use a multi-layer graph convolutional network (GCN) for feature extraction of the molecular graph as follows:

(5) $$f_{g}^{i}\;=\sigma \;\left({\tilde{D}}^{-\frac{1}{2}}\tilde{A}{\tilde{D}}^{-\frac{1}{2}}X_{g}^{\left(i-1\right)}W^{\left(i-1\right)}\right),$$

(6) $$\tilde{A}=A+I,$$

where σ is the Relu activation function and *W* is the trainable weight matrix. *X* is the feature matrix and *A* is the edge matrix.

#### 2.2.3 Multi-view fusion on molecular encoding

To prevent feature offset, SMILES features and graph features of the drug are normalized separately. Then, SMILES features of drugs as queries (*Q*) and graph features as keys (*K*) and values (*V*). Taking the *i*th layer as an example, the relationship is expressed as follows:

(7) $$Q_{s}^{i},\;K_{g}^{i},\;V_{g}^{i}\;=\;\mathrm{Norm}(f_{s}^{i})W_{Q}^{i},\;\mathrm{Norm}(f_{g}^{i})W_{K}^{i},\;\mathrm{Norm}(f_{g}^{i})W_{V}^{i},$$

where $W_{Q}^{i}$, $W_{K}^{i}$, $W_{V}^{i}$ are learnable weight matrices. Norm is layer normalization.

We apply QKV attention to $\left\{Q_{s}^{i},\;K_{g}^{i},\;V_{g}^{i}\right\}$, where SMILES represents the extracted useful structural information from graph features. Finally, the extracted useful structural information is residually connected with the original SMILES features to obtain the final feature representation of the drug, as follows:

(8) $$f_{d}^{i}\;=\;\mathrm{Norm}\left(f_{s}^{i}\right)+\;{\mathrm{CNN}}_{1D}\left(A\left(Q_{s}^{i},\;K_{g}^{i},\;V_{g}^{i}\right)\right),$$

where *A* represents QKV Attention, CNN refers to a 1D convolution operation.

#### 2.2.4 Adaptive feature extraction

In order to obtain effective feature representation and explore the interaction mechanism between drugs and proteins, we transfer the features of drugs and proteins to linear attention layers. The specific calculation method is as follows:

(9) $$\alpha =\;\mathrm{softmax}\left(W_{2}t\mathrm{anh}\left(W_{1}X\right)\right),$$

(10) $$\tilde{X}=\alpha X.$$

Equation (9) can be deemed as a 2-layer multilayer perceptron (MLP) without bias, and the parameters are $\left\{W_{1},W_{2}\right\}$.

For drugs and proteins, we use linear attention to extract effective features:

(11) $${\tilde{f}}_{t}\;=\mathrm{LinearAttention}\left(f_{t}\right),$$

(12) $${\tilde{f}}_{d}\;=\mathrm{LinearAttention}\left(f_{d}\right).$$

In order to extract the interaction features of drugs and proteins, we concatenate the protein and drug features that have been linearly transformed and use linear attention for the learning of interaction information, which is represented as follows:

(13) $${\tilde{f}}_{c}\;=\;\mathrm{LinearAttention}\left(\mathrm{Concat}\left(f_{t},f_{d}\right)\right).$$

Finally, we feed the obtained features to the fully connected layer for DTI or DTA prediction as follows:

(14) $$y_{\mathrm{pre}}\;=\;FC\left(\mathrm{Concat}\left({\tilde{f}}_{d},{\tilde{f}}_{c},{\tilde{f}}_{t}\right)\right).$$

## 3 Results

### 3.1 Performance evaluation

####  

Our experiments split the training, validation, and test sets in a ratio of 8:1:1. For the random setting, Davis dataset was split into 24 044, 3006, and 3006 samples for the training, validation, and test sets, respectively. For the PDBbind dataset, since the protein and drug data in the test set overlap with the training set, and to be consistent with previous methods, we report the best results. All datasets except PDBbind use the average results of five random tests. The detailed training process and hyperparameters setting are released in Supplementary File S1, available as supplementary data at *Bioinformatics* online.

We evaluate the performance of the PMMR using mean squared error (MSE), root mean squared error (RMSE), mean absolute error (MAE), Pearson correlation coefficient (PCC), Spearman’s rank correlation coefficient (SCC), concordance index (CI) (Gönen and Heller 2005), and mean reversion ($r_{m}^{2}$) on the DTA datasets. At the same time, we use area under the precision–recall curve (AUPR) and area under the receiver operating characteristic curve (AUROC) to evaluate the classification performance of the PMMR on the DTI dataset. The detailed calculation formulas are published in the Supplementary File S1, available as supplementary data at *Bioinformatics* online.

### 3.2 Comparative experiments

We first compare sequence- and structure-based methods on the Davis dataset and PDBbind dataset. According to Table 2, on the Davis dataset, PMMR achieved the best results across all indicators compared to all mainstream methods. Compared to MFR-DTA (Hua *et al.* 2023a,b), our method achieved improvements of 0.027, 0.005, and 0.046 in MSE, CI, and $r_{m}^{2}$ indices, respectively. To our knowledge, this also marks the first time that the $r_{m}^{2}$ value has exceeded 0.75.

**Table 2.** The performance of PMMR and other mainstream methods on Davis dataset.^a^

Method	MSE (SD)	CI (SD)	$r_{m}^{2}$ (SD)
DeepDTA	0.261	0.878(0.004)	0.630(0.017)
GrapDTA	0.233(0.004)	0.890	0.663(0.010)
MRBDTA	0.216(0.006)	0.901(0.004)	0.716(0.008)
MATT_DTI	0.229	0.890(0.003)	0.682(0.009)
MGraphDTA	0.207(0.001)	0.900(0.004)	0.710(0.005)
MT-DTI	0.245	0.887	0.665
MFR-DTA	0.221(0.001)	0.905(0.001)	0.705(0.003)
PMMR	**0.194(0.006)**	**0.910(0.004)**	**0.751(0.007)**

a The bold corresponds to the best performance for each metric.

As shown in Table 3, we compared our method with other mainstream approaches. Due to the nonoverlapping nature of the training and test data in the PDBbind dataset, this posed a challenge to the predictive accuracy of the models. Compared to the second-best method TANKBind (Lu *et al.* 2022), our proposed method PMMR achieved improvements of 0.006, 0.008, 0.09, and 0.124 in RMSE, MAE, Pearson, and Spearman indices, respectively. This improvement is mainly attributed to our comprehensive integration of drug SMILES and graph representation information, significantly enhancing the model's predictive accuracy.

**Table 3.** The performance of PMMR and other mainstream methods on PDBbind dataset.^a^

Method	RMSE	MAE	Pearson	Spearman
DeepGLSTM	1.636	1.177	0.676	0.673
DGDTA	1.679	1.243	0.664	0.672
GraphDTA	1.658	1.288	0.675	0.678
TANKBind	1.346	1.070	0.726	0.703
STAMP-DPI	1.658	1.325	0.545	0.411
PMMR	**1.340**	**1.062**	**0.816**	**0.827**

a The bold corresponds to the best performance for each metric.

### 3.3 Out-of-domain generalization testing

In this section, we evaluated the performance of PMMR on the Therapeautics Data Commons Domain Generalization (TDC-DG) dataset. This dataset divides the collected affinity data into a training set and a test set according to years. Since the data in the training set is likely not to be leaked into the test set, this puts higher requirements on the generalization performance of the model. As shown in Table 4, compared with other methods, our proposed method PMMR shows the best results on the Pearson index.

**Table 4.** The performance of PMMR and other mainstream methods on TDC-DG dataset.^a^

Method	Pearson (SD)
PMMR	**0.595(0.004)**
OTTER-KNOWLEDGE	0.588(0.002)
ProBertMorgan	0.538(0.008)
MMD	0.433(0.010)
CORAL	0.432(0.010)
ERM	0.427(0.012)
MTL	0.425(0.010)
GroupDRO	0.384(0.006)
AndMASK	0.288(0.019)

a The bold corresponds to the best performance for each metric.

### 3.4 Cold-start generalization testing

Most previous methods use random splits to partition training, validation, and test sets. This random division may lead to overly optimistic results, as it can cause drug and protein information to leak into the test set (Mayr *et al.* 2018). But in real drug discovery scenarios, models need to infer unseen drugs, unseen targets, and unseen drug–target pairs. Therefore, in the cold-start scenario, in order to be more consistent with the actual situation, we use three different cold-start methods: drug cold-start, target cold-start, and all cold-start. Taking target cold-start as an example, the proteins in the training set are not visible in the test set. By comparing PMMR with existing mainstream methods across different scenarios, we demonstrate its advantages in generalization ability and robustness.

As shown in Table 5, in the drug cold-start scenario, PMMR significantly outperforms other methods across key metrics such as CI, and $r_{m}^{2}$, with scores of 0.794 and 0.325, respectively, representing an increase of 0.057 and 0.133. In the target cold-start scenario, compared with other mainstream methods, PMMR has achieved the best results in various indices. PMMR exhibits strong robustness and generalization when dealing with unknown drug–target pairs. Compared with FusionDTA (Yuan *et al.* 2022), PMMR has shown improvements of 0.326, 0.038, and 0.119 in MSE, CI, and $r_{m}^{2}$ indices, respectively. This significant improvement demonstrates that pre-training has a certain effect in alleviating the issue of data limitations. In short, PMMR showed excellent performance in different cold-start scenarios, demonstrating its ability to be applied in practical drug discovery scenarios.

**Table 5.** The performance of drug cold-start, target cold-start, and all cold-start on Davis dataset.^a^

	MSE (SD)	CI (SD)	$r_{m}^{2}\;$ (SD)
**Drug cold-start**			
GraphDTA	0.920(0.029)	0.678(0.036)	0.160(0.019)
GEFA	0.847(0.012)	0.709(0.028)	0.182(0.015)
FusionDTA	0.581(0.094)	0.737(0.012)	0.187(0.034)
MGraphDTA	0.563(0.065)	0.729(0.022)	0.192(0.021)
PMMR	**0.517(0.042)**	**0.794(0.026)**	**0.325(0.086)**
**Target cold-start**			
GraphDTA	0.510(0.086)	0.729(0.012)	0.154(0.014)
GEFA	0.433(0.022)	0.759(0.009)	0.289(0.016)
FusionDTA	0.364(0.021)	0.826(0.011)	0.435(0.023)
MGraphDTA	0.359(0.023)	0.813(0.008)	0.425(0.028)
PMMR	**0.329(0.021)**	**0.833(0.013)**	**0.471(0.030)**
**All cold-start**			
GraphDTA	0.968(0.096)	0.579(0.017)	0.026(0.016)
GEFA	0.944(0.092)	0.610(0.029)	0.032(0.022)
FusionDTA	0.876(0.091)	0.645(0.043)	0.072(0.048)
MGraphDTA	0.874(0.090)	0.636(0.021)	0.071(0.041)
PMMR	**0.550(0.084)**	**0.683(0.020)**	**0.191(0.061)**

a The bold corresponds to the best performance for each metric.

### 3.5 Classification performance evaluation

**Figure 2.**
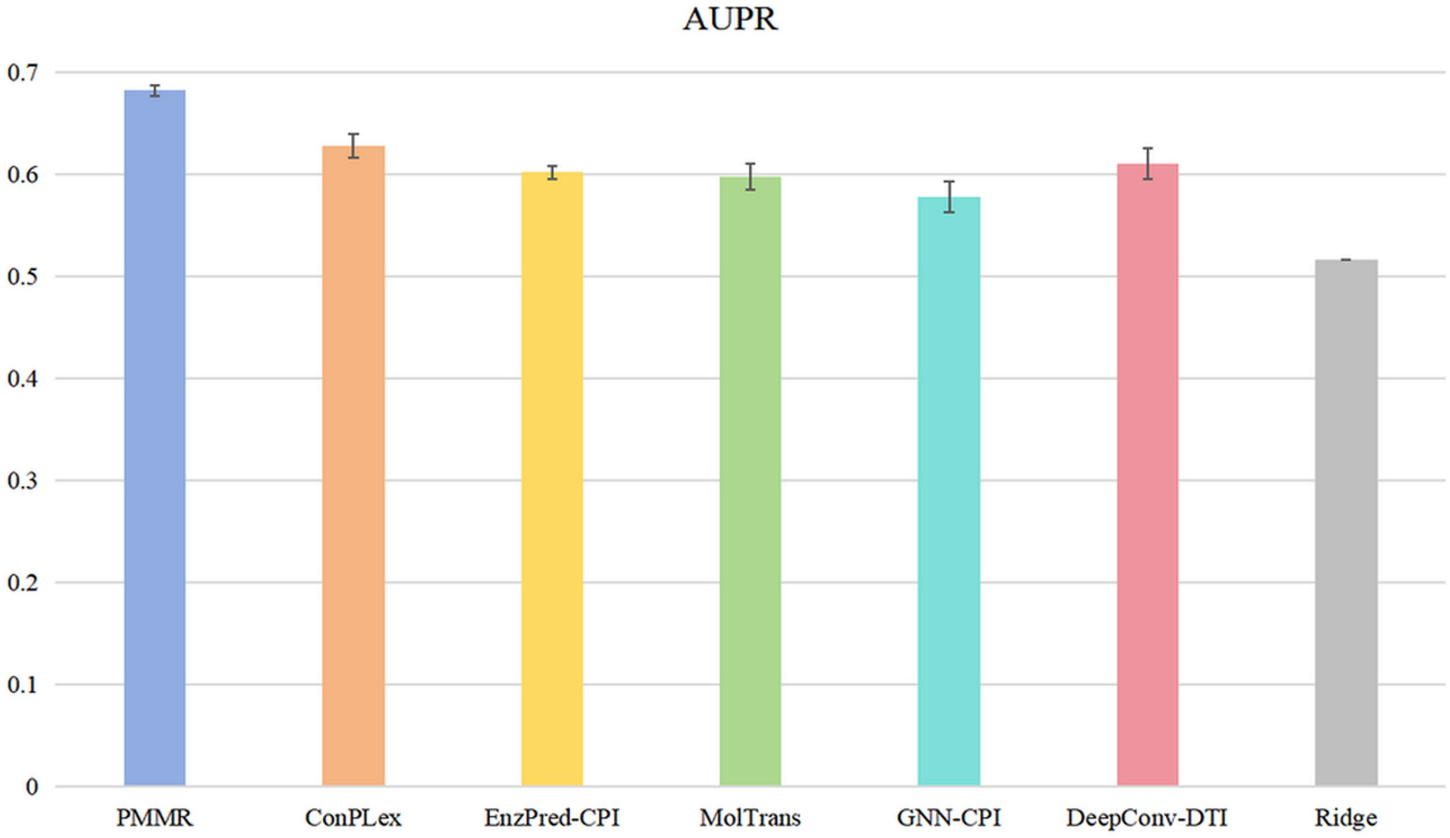
The average AUPR values of different methods after five random tests on the BindingDB dataset.

Although we have demonstrated the performance of PMMR in affinity prediction task, due to the generality of various features in drug–target binding prediction, we can easily convert the affinity prediction task into a binary classification task of interaction prediction. Therefore, based on the BindingDB dataset, we compared the AUPR values of five random tests of PMMR with methods such as ConPLex (Singh *et al.* 2023) and EnzPred-CPI (Goldman *et al.* 2022). And the AUROC curves of the five random tests are shown in Supplementary Fig. S1, available as supplementary data at *Bioinformatics* online. As shown in Fig. 2, PMMR’s AUPR value of 0.682 represents a 5.4% increase compared to ConPLex's AUPR value of 0.628, which also illustrates the excellent performance of our model in handling classification tasks.

### 3.6 The effect of various PLMs

**Figure 3.**
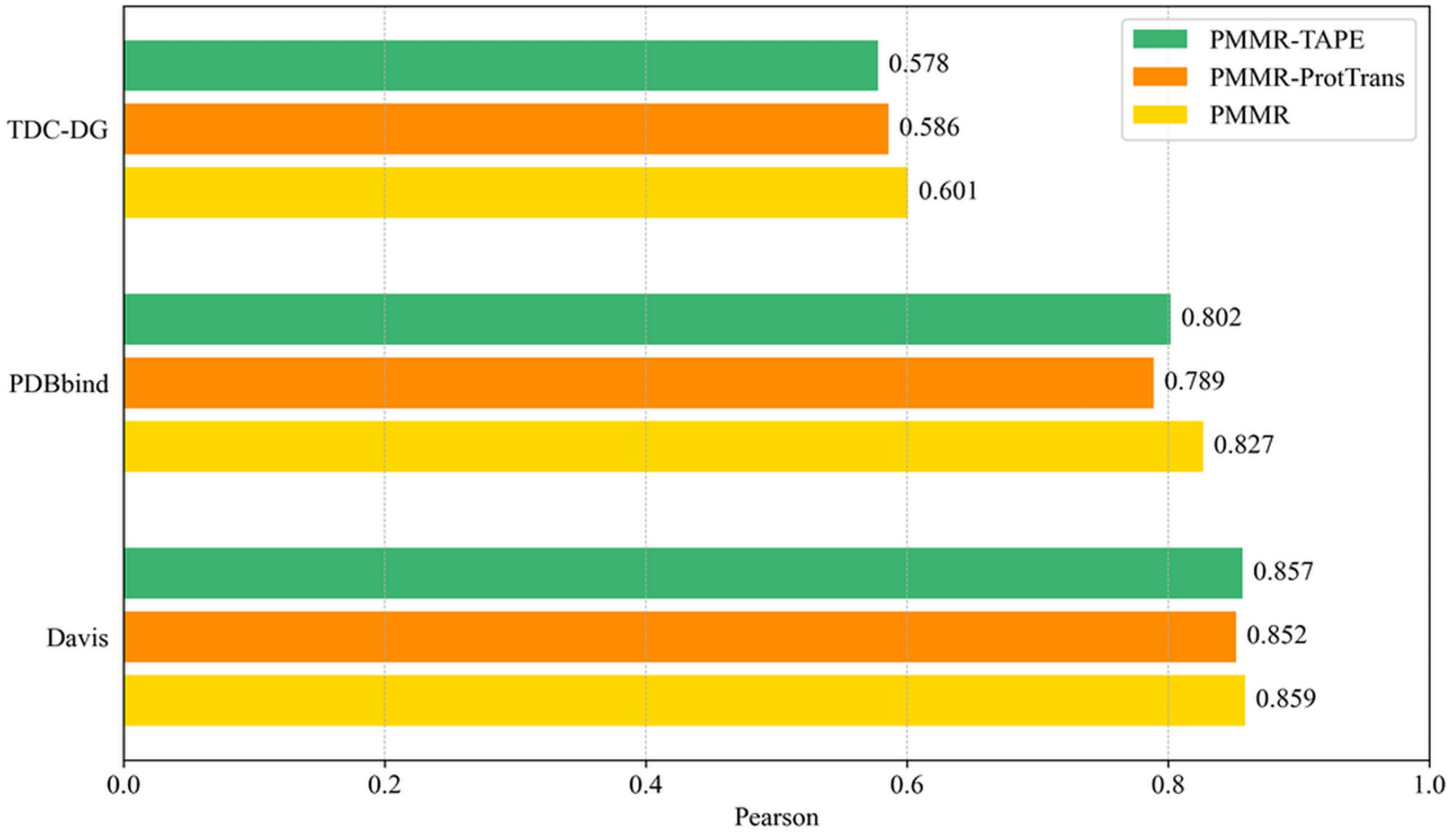
The evaluation of pre-trained features generated by different protein language models (PLMs) on three benchmark affinity datasets.

In this study, we explored several choices for using protein language models (PLMs) to generate sequence features, including ESM-2 (PMMR), ProtTrans (PMMR-ProtTrans) (Elnaggar *et al.* 2022), and TAPE (PMMR-TAPE) (Rao *et al.* 2019). All models provide per-residue features, which allows us to use the Transformer to fine-tune each residue feature in the sequence. As show in Fig. 3, we tested the impact of different pre-training methods on model performance on three benchmark affinity datasets. Judging from the Pearson value, the method of pre-training using ESM-2 showed the best performance on all datasets. Similarly, using TAPE or ProtTrans as a pre-training method achieved similar results on all benchmark datasets. At the same time, the Spearman results of the three pre-training methods are provided in Supplementary Fig. S2, available as supplementary data at *Bioinformatics* online.

### 3.7 The effect of different view features on drug molecules

**Figure 4.**
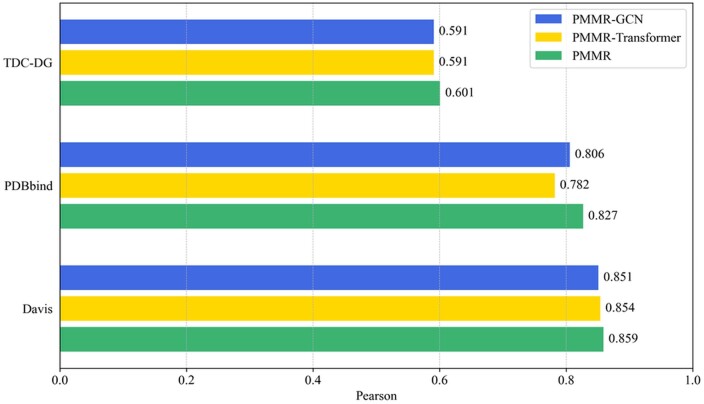
The impact of different view features of drugs on model performance was evaluated across three benchmark affinity datasets.

In order to verify whether the SMILES and graph features of drug molecules can have complementary effects. We set up two model variants, PMMR-GCN and PMMR-Transformer. PMMR-GCN is a model that uses molecular graphs. PMMR-Transformer is a model that fine-tunes the pre-trained features generated by ChemBERTa-2 through Transformer. As shown in Fig. 4, in both the TDC-DG dataset and the Davis dataset, PMMR achieved the best results using two-view features, while methods like PMMR-GCN and PMMR-Transformer, which utilized single-view features, obtained similar results. These findings demonstrate that under the influence of the drug decoder, PMMR effectively integrates both the SMILES and graph information of drug molecules, thereby improving the prediction accuracy of the model. Similarly, we provide Spearman results for different view features on three benchmark affinity datasets in Supplementary Fig. S3, available as supplementary data at *Bioinformatics* online.

## 4 Interpretability analysis

Traditional deep learning methods for drug–target prediction are usually a black box model, which poses a challenge to understanding the interaction between target proteins and drugs. With the development of the attention mechanism, it becomes possible to convert from black box mode to white box mode. In this study, since PMMR contains a linear attention mechanism, this allows us to explore the interaction mechanism between drugs and targets by analyzing attention weights.

**Figure 5.**
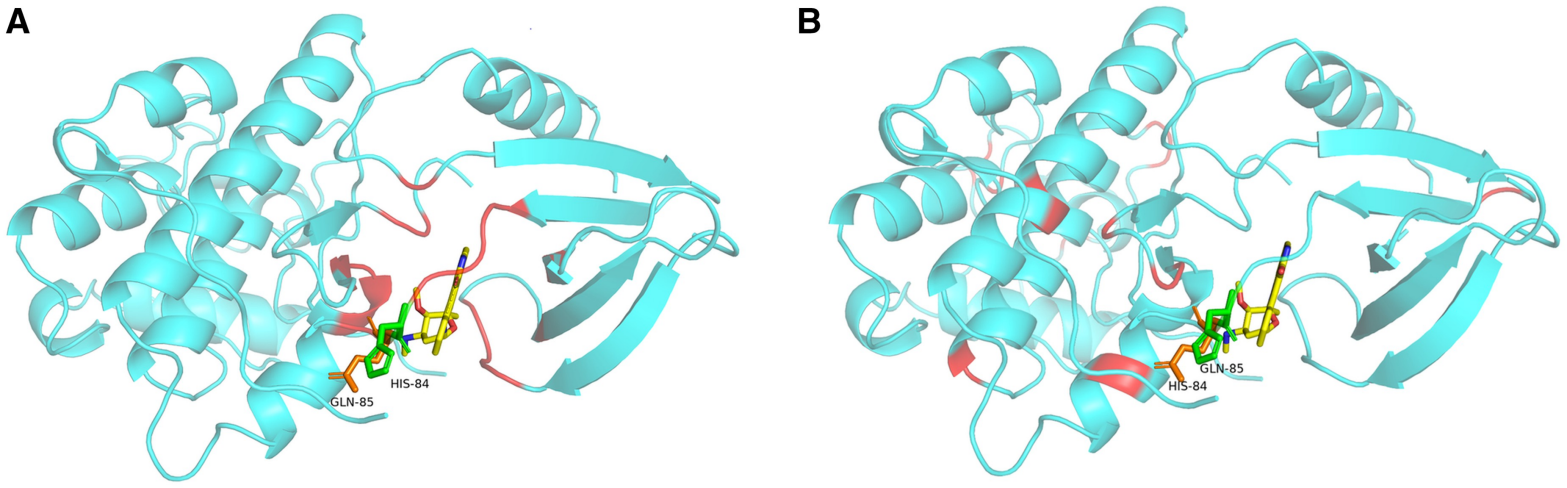
The visualization of interaction sites in 1AQ1. (A) Potential interaction sites. (B) Predicted interaction sites.

We selected 1AQ1 in the Protein Data Bank (PDB) database (Rose *et al.* 2017) as a case for weight visualization. Typically, in PDB database, potential interaction sites between drug and protein are defined by distances in the range < 5.0 angstroms between all amino acid residues of the drug and protein (Abbasi *et al.* 2020). We select amino acid residues with higher attention weight values as interaction sites predicted by PMMR, and the number of selected amino acid residues is equal to the number of potential interaction sites. As shown in Fig. 5, the potential and predicted interaction sites of 1AQ1 are marked in red respectively. The model correctly predicted potential interaction sites in the binding pocket with probability of binding: HIS-84 and GLN-85. Furthermore, the ASN136-GLY139 binding site was incorrectly predicted. Although PMMR incorrectly predicts some regions that may not bind, it can still focus on residues with binding potential, suggesting that it has some interpretability for exploring drug–target pairs.

## 5 Case study

**Figure 6.**
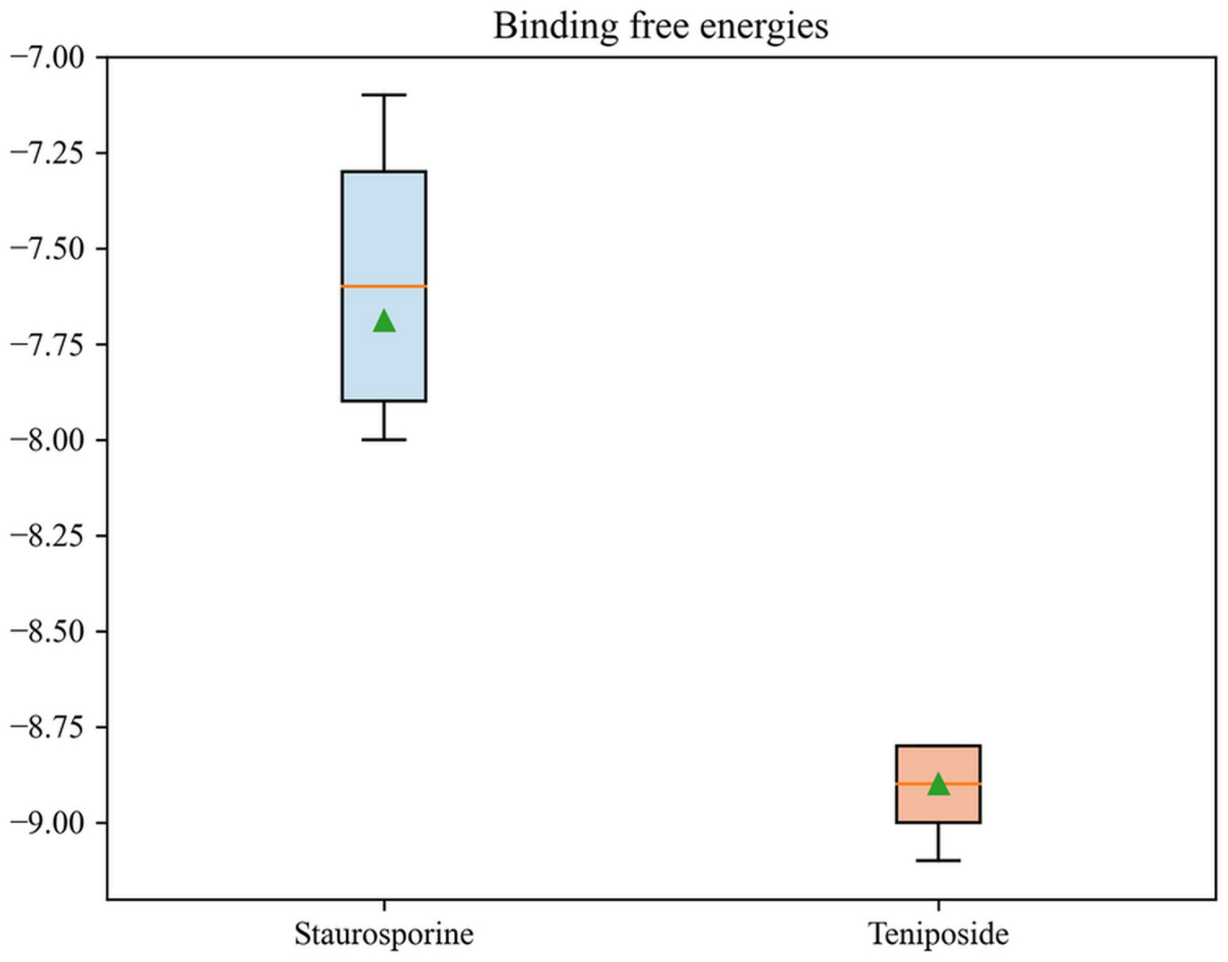
Binding free energies of CDK2 with staurosporine and teniposide.

CDK2 is a critical modulator of various oncogenic signaling pathways, and its activity is vital for loss of proliferative control during oncogenesis (Horiuchi *et al.* 2012). The selective CDK2 inhibition may provide a therapeutic benefit against certain tumors, and it continues to appeal as a strategy to exploit in anticancer drug development. Therefore, to explore possible inhibitors of CDK2, we predicted the affinity values ($K_{d}$) of CDK2 and 3137 FDA-approved drugs (Dittmar *et al.* 2021). Among the top 2 drugs with predicted affinity (Supplementary File S2, available as supplementary data at *Bioinformatics* online), staurosporine (the second-ranked drug) is a potent protein kinase C inhibitor that enhances cAMP-mediated responses in human neuroblastoma cells. According to DrugBank (Wishart *et al.* 2008) records, staurosporine (DB02010) is a drug known to bind to CDK2. It is worth noting that the number one drug, teniposide, is a semi-synthetic derivative of podophyllotoxin. Although its interaction with CDK2 has not been documented, its antitumor activity (Muggia 1994) suggests that it has a high potential to react with CDK2. The molecular docking results show that among the nine binding conformations, the binding free energy of teniposide and CDK2 is generally lower than that of staurosporine (Fig. 6). The median binding free energy of teniposide to CDK2 is −8.9 kcal/mol, significantly lower than that of staurosporine to CDK2: −7.6 kcal/mol.

**Figure 7.**
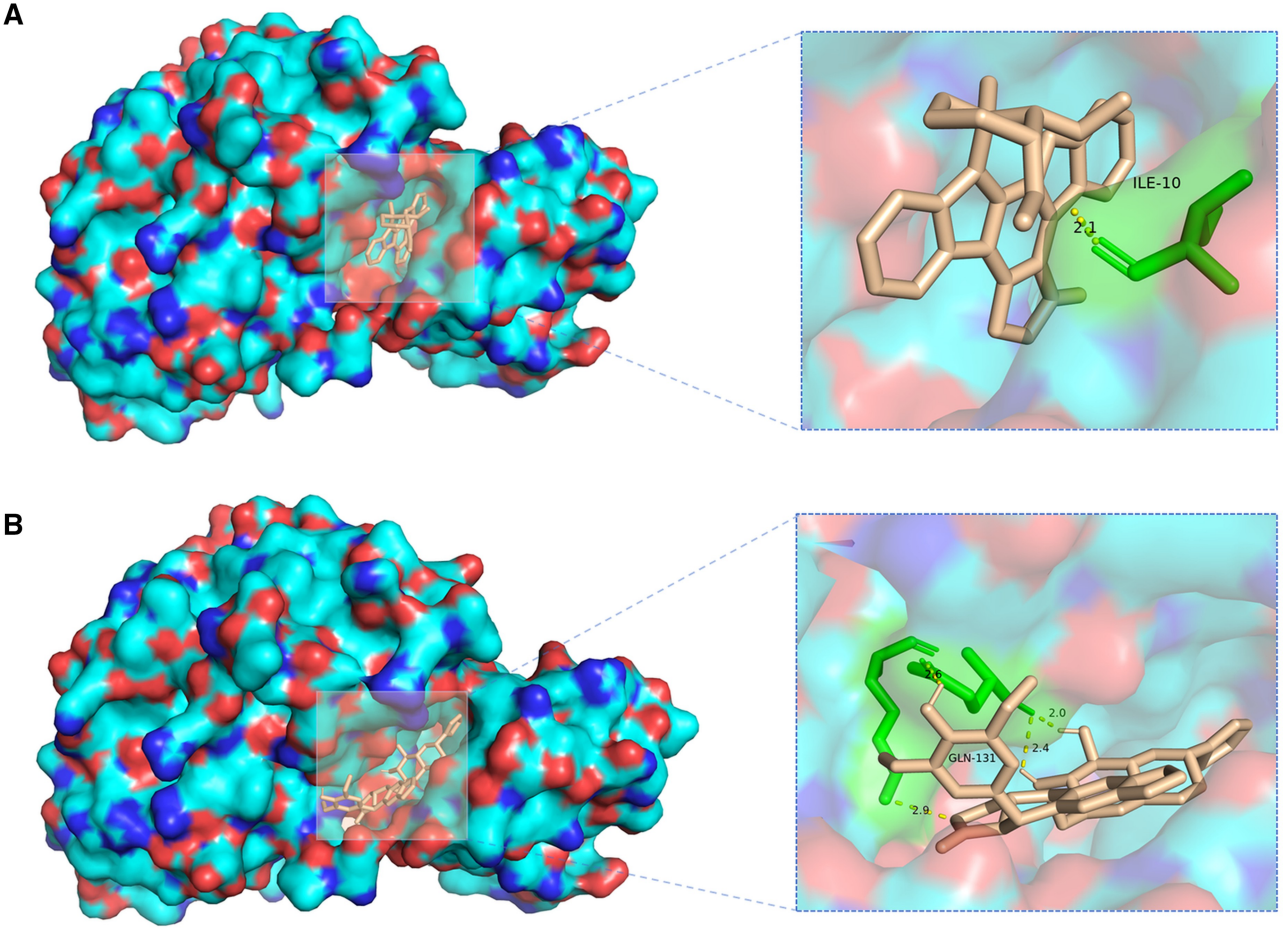
The visualization of docking results. (A) Binding conformation of CDK2 with staurosporine. (B) Binding conformation of CDK2 and teniposide.

Moreover, we further visualized the molecular docking results. As shown in the upper part of Fig. 7, the ILE-10 residue in CDK2 undergoes a hydrogen bonding interaction with staurosporine. In the lower part of Fig. 7, residues such as GLN-131 in CDK2 have hydrogen bond interactions with teniposide. Therefore, whether from the prediction results or the docking results, teniposide is likely to interact with CDK2, which provides certain reference for the next step of research.

## 6 Conclusion

In this work, we proposed PMMR, a method that PMMRs for drug–target binding prediction. The main contribution of this work is to utilize pre-training methods to address the challenge of limited availability and diversity of datasets, and to fully integrate the information from the multi-view of the drug. PMMR demonstrated promising results when tested on various affinity and interaction datasets. Meanwhile, in the cold-start scenario, PMMR showed excellent performance beyond previous methods. Finally, through interpretability analysis and a case study of CDK2, it was proved that PMMR has the ability to explore drug–target interaction mechanisms and be applied to real drug discovery scenarios.

## Supplementary Material

btaf002_Supplementary_Data

## Data Availability

Our study used open-access datasets, and both these datasets and the source code of PMMR are available in the GitHub repository mentioned above.

## References

[btaf002-B1] Abbasi K , Razzaghi P, Poso A et al DeepCDA: deep cross-domain compound–protein affinity prediction through LSTM and convolutional neural networks. Bioinformatics 2020;36:4633–42. 32462178 10.1093/bioinformatics/btaa544

[btaf002-B2] Ahmad W, Simon E, Chithrananda S et al Chemberta-2: towards chemical foundation models. arXiv, arXiv:2209.01712, 2022, preprint: not peer reviewed.

[btaf002-B4] Bi X , Zhang S, Ma W et al HiSIF-DTA: a hierarchical semantic information fusion framework for drug–target affinity prediction. IEEE J Biomed Health Inform 2023. 10.1109/JBHI.2023.3334239 37983161

[btaf002-B5] Burley SK , Berman HM, Bhikadiya C et al RCSB protein data bank: biological macromolecular structures enabling research and education in fundamental biology, biomedicine, biotechnology and energy. Nucleic Acids Res 2019;47:D464–74. 30357411 10.1093/nar/gky1004 PMC6324064

[btaf002-B6] Dara S , Dhamercherla S, Jadav SS et al Machine learning in drug discovery: a review. Artif Intell Rev 2022;55:1947–99. 34393317 10.1007/s10462-021-10058-4 PMC8356896

[btaf002-B7] Davis MI , Hunt JP, Herrgard S et al Comprehensive analysis of kinase inhibitor selectivity. Nat Biotechnol 2011;29:1046–51. 22037378 10.1038/nbt.1990

[btaf002-B8] Dittmar M , Lee JS, Whig K et al Drug repurposing screens reveal cell-type-specific entry pathways and FDA-approved drugs active against SARS-Cov-2. Cell Rep 2021;35:108959. 33811811 10.1016/j.celrep.2021.108959 PMC7985926

[btaf002-B9] Elnaggar A , Heinzinger M, Dallago C et al ProtTrans: toward understanding the language of life through self-supervised learning. IEEE Trans Pattern Anal Mach Intell 2022;44:7112–27. 34232869 10.1109/TPAMI.2021.3095381

[btaf002-B10] Forli S , Huey R, Pique ME et al Computational protein–ligand docking and virtual drug screening with the AutoDock suite. Nat Protoc 2016;11:905–19. 27077332 10.1038/nprot.2016.051 PMC4868550

[btaf002-B11] Gim M , Choe J, Baek S et al ArkDTA: attention regularization guided by non-covalent interactions for explainable drug–target binding affinity prediction. Bioinformatics 2023;39:i448–i457. 37387164 10.1093/bioinformatics/btad207 PMC10311339

[btaf002-B12] Goldman S , Das R, Yang KK et al Machine learning modeling of family wide enzyme–substrate specificity screens. PLoS Comput Biol 2022;18:e1009853. 35143485 10.1371/journal.pcbi.1009853 PMC8865696

[btaf002-B13] Gönen M , Heller G. Concordance probability and discriminatory power in proportional hazards regression. Biometrika 2005;92:965–70.

[btaf002-B14] Horiuchi D , Huskey NE, Kusdra L et al Chemical-genetic analysis of cyclin dependent kinase 2 function reveals an important role in cellular transformation by multiple oncogenic pathways. Proc Natl Acad Sci USA 2012;109:E1019–27. 22474407 10.1073/pnas.1111317109 PMC3340028

[btaf002-B15] Hua Y , Song X, Feng Z et al CPInformer for efficient and robust compound–protein interaction prediction. IEEE/ACM Trans Comput Biol Bioinf2023a;20:285–96. 10.1109/TCBB.2022.3144008 35044921

[btaf002-B16] Hua Y , Song X, Feng Z et al MFR-DTA: a multi-functional and robust model for predicting drug–target binding affinity and region. Bioinformatics2023b;39:btad056. 36708000 10.1093/bioinformatics/btad056 PMC9900210

[btaf002-B17] Huang K, Fu T, Gao W et al Therapeutics data commons: machine learning datasets and tasks for drug discovery and development. arXiv, arXiv:2102.09548, 2021, preprint: not peer reviewed.

[btaf002-B18] Huang K , Xiao C, Glass LM et al MolTrans: molecular interaction transformer for drug–target interaction prediction. Bioinformatics 2021;37:830–6. 33070179 10.1093/bioinformatics/btaa880 PMC8098026

[btaf002-B19] Jin Y , Lu J, Shi R et al EmbedDTI: enhancing the molecular representations via sequence embedding and graph convolutional network for the prediction of drug–target interaction. Biomolecules 2021;11:1783. 34944427 10.3390/biom11121783 PMC8698792

[btaf002-B20] Landrum G. RDKit: a software suite for cheminformatics, computational chemistry, and predictive modeling. Greg Landrum 2013;8:31.

[btaf002-B21] Li F , Zhang Z, Guan J et al Effective drug–target interaction prediction with mutual interaction neural network. Bioinformatics 2022;38:3582–9. 35652721 10.1093/bioinformatics/btac377 PMC9272808

[btaf002-B22] Lin Z , Akin H, Rao R et al Evolutionary-scale prediction of atomic-level protein structure with a language model. Science 2023;379:1123–30. 36927031 10.1126/science.ade2574

[btaf002-B23] Liu T , Lin Y, Wen X et al BindingDB: a web-accessible database of experimentally determined protein–ligand binding affinities. Nucleic Acids Res 2007;35:D198–201. 17145705 10.1093/nar/gkl999 PMC1751547

[btaf002-B24] Liu Y , Xing L, Zhang L et al GEFormerDTA: drug target affinity prediction based on transformer graph for early fusion. Sci Rep 2024;14:7416. 38548825 10.1038/s41598-024-57879-1 PMC10979032

[btaf002-B25] Liu Z , Su M, Han L et al Forging the basis for developing protein–ligand interaction scoring functions. Acc Chem Res 2017;50:302–9. 28182403 10.1021/acs.accounts.6b00491

[btaf002-B26] Lu W, Wu Q, Zhang J et al Tankbind: Trigonometry-aware neural networks for drug-protein binding structure prediction. Adv Neural Inf Process Syst 2022;35:7236–49.

[btaf002-B27] Malathi K , Ramaiah S. Bioinformatics approaches for new drug discovery: a review. Biotechnol Genet Eng Rev 2018;34:243–60. 30064294 10.1080/02648725.2018.1502984

[btaf002-B28] Mayr A , Klambauer G, Unterthiner T et al Large-scale comparison of machine learning methods for drug target prediction on ChEMBL. Chem Sci 2018;9:5441–51. 30155234 10.1039/c8sc00148k PMC6011237

[btaf002-B29] Muggia FM. Teniposide: overview of its therapeutic potential in adult cancers. Cancer Chemotherapy Pharmacol 1994;34:S127–S133. 10.1007/BF00684876 8070021

[btaf002-B30] Nguyen T , Le H, Quinn TP et al GraphDTA: predicting drug–target binding affinity with graph neural networks. Bioinformatics 2021;37:1140–7. 33119053 10.1093/bioinformatics/btaa921

[btaf002-B31] Peng L , Liu X, Yang L et al BINDTI: a bi-directional intention network for drug–target interaction identification based on attention mechanisms. IEEE J Biomed Health Inform 2024. 10.1109/JBHI.2024.3375025 38457318

[btaf002-B32] Rao R , Bhattacharya N, Thomas N et al Evaluating protein transfer learning with TAPE. Adv Neural Inf Process Syst 2019;32:9689–701. 33390682 PMC7774645

[btaf002-B33] Rose PW , Prlić A, Altunkaya A et al The RCSB protein data bank: integrative view of protein, gene and 3D structural information. Nucleic Acids Res 2017;45:D271–81. 27794042 10.1093/nar/gkw1000 PMC5210513

[btaf002-B34] Schneider G. Automating drug discovery. Nat Rev Drug Discov 2018;17:97–113. 29242609 10.1038/nrd.2017.232

[btaf002-B35] Singh R , Sledzieski S, Bryson B et al Contrastive learning in protein language space predicts interactions between drugs and protein targets. Proc Natl Acad Sci USA 2023;120:e2220778120. 37289807 10.1073/pnas.2220778120 PMC10268324

[btaf002-B36] Vaswani A, Shazeer N, Parmar N et al Attention is all you need. Adv Neural Inf Process Syst 2017;31:5998–6008.

[btaf002-B37] Wang K , Zhou R, Li Y et al DeepDTAF: a deep learning method to predict protein–ligand binding affinity. Brief Bioinform 2021;22:bbab072. 33834190 10.1093/bib/bbab072

[btaf002-B3] Weininger D. SMILES, a chemical language and information system. 1. Introduction to methodology and encoding rules. J Chem Inf Comput Sci 1988;28:31–6.

[btaf002-B38] Wishart DS , Knox C, Guo AC et al DrugBank: a knowledgebase for drugs, drug actions and drug targets. Nucleic Acids Res 2008;36:D901–6. 18048412 10.1093/nar/gkm958 PMC2238889

[btaf002-B39] Yan Y , Zhang D, Zhou P et al HDOCK: a web server for protein–protein and protein–DNA/RNA docking based on a hybrid strategy. Nucleic Acids Res 2017;45:W365–73. 28521030 10.1093/nar/gkx407 PMC5793843

[btaf002-B40] Yang Z , Zhong W, Zhao L et al MGraphDTA: deep multiscale graph neural network for explainable drug–target binding affinity prediction. Chem Sci 2022;13:816–33. 35173947 10.1039/d1sc05180f PMC8768884

[btaf002-B41] Yuan W , Chen G, Chen CY-C. FusionDTA: attention-based feature polymerizer and knowledge distillation for drug–target binding affinity prediction. Brief Bioinform 2022;23:bbab506. 34929738 10.1093/bib/bbab506

[btaf002-B42] Zhang L , Wang C-C, Chen X. Predicting drug–target binding affinity through molecule representation block based on multi-head attention and skip connection. Brief Bioinform 2022;23:bbac468. 36411674 10.1093/bib/bbac468

[btaf002-B43] Zhang Y , Hu Y, Han N et al A survey of drug–target interaction and affinity prediction methods via graph neural networks. Comput Biol Med 2023;163:107136. 37329615 10.1016/j.compbiomed.2023.107136

