# Supplementary material for: Improving generalizability of drug–target binding prediction by pre-trained multi-view molecular representations
Source: Bioinformatics. 2025 Jan 7;41(1):btaf002. doi: 10.1093/bioinformatics/btaf002 (PMC11751634; doi:10.1093/bioinformatics/btaf002)
Supplement: btaf002_Supplementary_Data [file btaf002_supplementary_data.zip › f1754_Supplementary File 2.pdf]

Predicted affinity values of CDK2 with 3137 FDA-approved drugs

Drug_Name	Affinity	Rank
Teniposide	8.678376	1
Staurosporine	8.544364	2
Vorapaxar	8.517833	3
Daclatasvir (BMS-790052)	8.459474	4
Meropenem	8.433542	5
Obeticholic Acid	8.403761	6
Brucine	8.385266	7
Meropenem trihydrate	8.375531	8
17-AAG (KOS953)	8.329867	9
Etoposide	8.325273	10
Quinapril HCl	8.324926	11
Metoprolol Tartrate	8.314344	12
Chenodeoxycholic Acid	8.313922	13
nystatin	8.297594	14
tunicamycin	8.289911	15
Vitamin D2 (Ergocalciferol)	8.253683	16
isometheptene	8.244697	17
radicicol	8.236606	18
Amikacin	8.223321	19
Birinapant (TL32711)	8.222646	20
Ispinesib (SB-715992)	8.21207	21
Lithocholic Acid	8.210523	22
lestaurtinib	8.191469	23
Caspofungin Acetate	8.190515	24
Ouabain Octahydrate	8.18309	25
Doxercalciferol	8.168772	26
Cholic acid	8.161698	27
Perindopril Erbumine	8.155984	28
Atazanavir	8.151569	29
Metoprolol Succinate	8.143267	30
Prilocaine hydrochloride	8.126141	31
Geniposide	8.114488	32
Articaine HCl	8.105653	33
Polydatin	8.105486	34
lovastatin	8.097644	35
doxycycline	8.084316	36
hydroxycholesterol	8.065481	37
Deoxycholic acid	8.065428	38
Flunixin Meglumin	8.054216	39
Calcium Gluceptate	8.046316	40
stiripentol	8.046141	41
Ginkgolide A	8.037993	42
dieldrin	8.036429	43
Pancuronium dibromide	8.018934	44
Imidapril HCl	8.017508	45
procaterol	8.005106	46
verrucarin-a	8.00148	47
MMPX	7.995329	48
Bisoprolol fumarate	7.9743185	49

Abiraterone	7.969803	50
Hygromycin B	7.955653	51
Elvitegravir (GS-9137)	7.952858	52
isorotenone	7.9524946	53
Streptozocin	7.9508457	54
Clindamycin HCl	7.946315	55
Esmolol HCl	7.9463024	56
Acebutolol HCl	7.943734	57
Oseltamivir	7.9305277	58
Alfacalcidol	7.9290037	59
ispinesib	7.9168367	60
buphenine	7.9041033	61
isoxsuprine	7.903244	62
acebutolol	7.901368	63
vancomycin	7.9010134	64
KW-06	7.8934393	65
kepone	7.892276	66
Ipratropium Bromide	7.889229	67
bimatoprost	7.878683	68
esmolol	7.878579	69
Oseltamivir acid	7.87356	70
cefoxitin	7.872365	71
Fidaxomicin	7.870817	72
Clindamycin	7.8647213	73
Netilmicin Sulfate	7.860218	74
Drospirenone	7.855692	75
Peramivir Trihydrate	7.8508806	76
isamoltane	7.8404207	77
orciprenaline	7.839595	78
lactotensin	7.8276415	79
metoprolol	7.8272457	80
Propranolol HCl	7.82286	81
Mevastatin	7.8118334	82
Caspofungin	7.8093596	83
eplerenone	7.7947016	84
Telbivudine	7.791215	85
Calcipotriol	7.7809334	86
Estradiol	7.7673616	87
propranolol	7.763074	88
pronetalol	7.74457	89
orthothymotinic-acid	7.744274	90
TOK-001	7.743595	91
Isoprenaline HCl	7.7408133	92
iproniazid	7.7380095	93
Artemisinine	7.7354765	94
Artemether	7.735181	95
Dutasteride	7.733449	96
minocycline	7.732935	97
methoxamine	7.7252197	98
Peramivir	7.7243958	99
atenolol-(+/-)	7.7220516	100

tolterodine	7.717985	101
Saxagliptin	7.706477	102
Spiramycin	7.701562	103
Stavudine (d4T)	7.7010355	104
Bestatin	7.6949015	105
Abiraterone acetate	7.689851	106
sibutramine	7.6636744	107
Estradiol valerate	7.6626453	108
suloctidil	7.6621666	109
artemisinin	7.656502	110
Clorprenaline HCL	7.6395817	111
artemether	7.6393642	112
Meglumine	7.6301203	113
Pravastatin sodium	7.629678	114
Dovitinib Dilactic acid	7.619087	115
pravastatin	7.615118	116
CEP-18770	7.6043024	117
naloxone	7.594199	118
Artesunate	7.5842724	119
nikkomycin	7.5685225	120
estriol	7.567458	121
avermectin	7.559265	122
acitretin	7.5589466	123
Nebivolol	7.558917	124
Mupirocin	7.5491266	125
ipratropium	7.5465126	126
Cytidine	7.529566	127
bongkrek-acid	7.5281267	128
Reserpine hydrochloride	7.5236645	129
aminobenztropine	7.5124025	130
Trilostane	7.506281	131
Tylosin tartrate	7.501239	132
oxandrolone	7.49148	133
Benazepril HCl	7.48421	134
ritodrine	7.472472	135
isoprenaline	7.4629755	136
levisoprenaline	7.462964	137
amantadine	7.4607706	138
Amphotericin B	7.4576683	139
ergometrine	7.448802	140
Epothilone B (EPO906, Patupilone)	7.445901	141
scopolamine	7.4414387	142
genipin	7.417656	143
Latanoprost	7.397249	144
Tobramycin	7.3819	145
Catharanthine	7.3697186	146
mocimycin	7.3602715	147
DC-45-A2	7.355996	148
alcuronium	7.350478	149
Dihydroartemisinin	7.342272	150
penitrem-a	7.3417063	151

elvitegravir	7.3363695	152
Tolterodine tartrate	7.328788	153
artesunate	7.3125944	154
monocrotaline	7.306074	155
triptolide	7.2941637	156
epothilone	7.288066	157
mestanolone	7.2841387	158
catharanthine	7.27232	159
Calcifediol	7.266549	160
Calcitriol	7.262534	161
chaetocin	7.2624826	162
perhexiline	7.259842	163
colistin	7.226225	164
Deoxycorticosterone acetate	7.2201185	165
vinorelbine	7.218028	166
doxercalciferol	7.2071	167
memantine	7.205496	168
topiramate	7.2023363	169
Topiramate	7.2016172	170
cyanocobalamin	7.1980658	171
Enalapril Maleate	7.1975546	172
Pregnenolone	7.1915627	173
monensin	7.1857224	174
dihydroergotamine	7.1846514	175
avrainvillamide-analog-3	7.179632	176
Digoxin	7.1771755	177
Micafungin sodium	7.1693783	178
Spectinomycin (hydrochloride hydrate)	7.157789	179
Leucovorin Calcium	7.1519947	180
LY2835219	7.147168	181
prenylamine	7.1360636	182
naltrindole	7.1243305	183
10-DAB (10-Deacetylbaccatin)	7.1237936	184
picrotoxin	7.120266	185
Roscovitine (Seliciclib,CYC202)	7.120097	186
Lovastatin	7.1133547	187
Spironolactone	7.1121464	188
Mestranol	7.1085806	189
Levonorgestrel	7.104065	190
Gestodene	7.0997734	191
latrunculin-b	7.0995874	192
strophanthidin	7.094304	193
mannitol	7.086381	194
seneciphylline	7.08459	195
myo-Inositol	7.076371	196
benzatropine	7.069131	197
canrenone	7.0509644	198
canrenoic-acid	7.0385637	199
Bromocriptine mesylate	7.033201	200
etodolac	7.029089	201
carisoprodol	7.024046	202

Mitomycin C	7.015417	203
Val-Val-Val	7.012231	204
mitomycin-c	7.008834	205
pyrithyldione	7.005245	206
ginsenoside	7.0027714	207
alpha-tocopherol	6.9876776	208
tibolone	6.986887	209
methyllycaconitine	6.9835634	210
Nystatin (Fungicidin)	6.9830914	211
Xylitol	6.9763517	212
gatifloxacin	6.969824	213
Labetalol HCl	6.9658947	214
estradiol	6.9518433	215
cymarin	6.9510136	216
zonisamide	6.9435167	217
Ivermectin	6.943474	218
Biapenem	6.937196	219
oxymetholone	6.934312	220
rifabutin	6.9341545	221
ursolic-acid	6.9307747	222
epitiostanol	6.9244375	223
Reserpine	6.9239655	224
Desogestrel	6.9163427	225
Epinephrine Bitartrate	6.9071445	226
fendiline	6.906271	227
pseudoephedrine	6.9052696	228
Zanamivir	6.9036045	229
bromocriptine	6.894223	230
Capecitabine	6.887274	231
melengestrol	6.883911	232
Prilocaine	6.8776035	233
Etodolac	6.86438	234
Vildagliptin (LAF-237)	6.8634844	235
androsterone	6.8629694	236
epiandrosterone	6.858782	237
Epleremone	6.8488503	238
hinokitiol	6.8146067	239
propantheline	6.811637	240
Vinorelbine ditartrate	6.804619	241
avrainvillamide-analog-5	6.8037987	242
Azelnidipine	6.7990227	243
efavirenz	6.798873	244
peruvoside	6.792306	245
Homatropine Methylbromide	6.7868423	246
brucine	6.7784834	247
AVA	6.7767296	248
crotamiton	6.7747173	249
Ramipril	6.763212	250
nadolol	6.7617292	251
Temocapril HCl	6.7568274	252
guggulsterone	6.7555304	253

epoxycholesterol	6.751292	254
Fluocinonide	6.745661	255
valyltryptophan	6.743046	256
PRIMA1	6.724845	257
nalbuphine	6.713151	258
ryanodine	6.7070723	259
ifenprodil	6.7008934	260
Bimatoprost	6.694868	261
n-acetyl-l-leucine	6.6799736	262
flunisolide	6.6798315	263
abiraterone	6.6699047	264
Rifabutin	6.665407	265
Geniposidic acid	6.6564445	266
equilin	6.656189	267
Memantine hydrochloride	6.652899	268
cholic-acid	6.650778	269
Vitamin B12	6.64336	270
halcinonide	6.642475	271
mestranol	6.636426	272
Methacycline HCl	6.6364	273
mecamylamine	6.6358156	274
formestane	6.632821	275
Vitamin C	6.6287203	276
troleandomycin	6.6195393	277
Xylose	6.618185	278
norgestimate	6.6157227	279
Bacitracin	6.6156406	280
amcinonide	6.6096725	281
Triamcinolone	6.6037188	282
prostratin	6.591467	283
chenodeoxycholic-acid	6.58587	284
Oxytetracycline (Terramycin)	6.5719757	285
ethylestrenol	6.5701036	286
VRT752271	6.56752	287
Dienogest	6.5639744	288
Novobiocin Sodium	6.5596895	289
propofol	6.555683	290
carnosol	6.5517845	291
MG-132	6.5509815	292
Valnemulin HCl	6.533966	293
BI6727 (Volasertib)	6.531226	294
penicillin	6.5258293	295
androstenedione	6.5101624	296
bambuterol	6.5025415	297
carbenoxolone	6.494693	298
parthenolide	6.492713	299
triamcinolone	6.4872894	300
E 64d	6.475971	301
androstanol	6.474824	302
oleanolic-acid	6.4743624	303
Chloramphenicol	6.4699564	304

parthenolide-(alternate-stereo)	6.4573383	305
anthothecol	6.457077	306
prometon	6.4548645	307
Galanthamine HBr	6.4489107	308
veratridine	6.445529	309
vindesine	6.442522	310
naltriben	6.439931	311
Doxycycline HCl	6.438554	312
Vinblastine sulfate	6.4290314	313
prednisone	6.423543	314
procarbazine	6.404249	315
Efavirenz	6.4023275	316
Cyproterone Acetate	6.4012403	317
galantamine	6.396366	318
atractyloside	6.3897142	319
Methylcobalamin	6.38422	320
reserpic-acid	6.3839283	321
Etonogestrel	6.3828707	322
cinobufagin	6.374567	323
(-)-Huperzine A	6.3729258	324
Finasteride	6.3728323	325
norgestrel	6.3705673	326
estropipate	6.368497	327
adrenosterone	6.3582463	328
danoprevir	6.3558245	329
oligomycin-a	6.354891	330
Ciprofibrate	6.3543196	331
beclometasone	6.349521	332
Hexestrol	6.3456244	333
Nateglinide	6.3330746	334
Desonide	6.3318458	335
Trimebutine	6.3291845	336
Tacrolimus (FK506)	6.3288565	337
stanozolol	6.3206115	338
Benztropine mesylate	6.310993	339
etiocholanolone	6.2971187	340
adapalene	6.282751	341
Fluvastatin	6.282125	342
Carbenicillin, Disodium Salt	6.28187	343
oxetane	6.280963	344
kitasamycin	6.27954	345
ivermectin	6.2780614	346
Rifampin	6.276699	347
CAM-9-026	6.274806	348
Griseofulvin	6.2734423	349
Doripenem	6.272712	350
Cabazitaxel	6.270188	351
gestrinone	6.268862	352
cantharidin	6.267294	353
Octreotide acetate	6.2597656	354
testosterone-propionate	6.251135	355

Norethindrone	6.247899	356
LE-135	6.247579	357
huperzine-a	6.2458973	358
RG7388	6.2444587	359
methysergide	6.240375	360
Estradiol Benzoate	6.227892	361
dextrorphan	6.226437	362
reichstein	6.210017	363
vincamine	6.2091413	364
thiostrepton	6.2087936	365
fludroxycortide	6.207766	366
atenolol	6.2067404	367
Nicotine Difartrate	6.2045956	368
Zidovudine	6.196233	369
2-Methoxyestradiol (2-MeOE2)	6.1944532	370
betulinic-acid	6.18075	371
celastrol	6.179281	372
hydrocortisone	6.1780605	373
roscovitine	6.1758747	374
acarbose	6.173003	375
Natamycin	6.1698027	376
digitoxin	6.1677904	377
Triamcinolone Acetonide	6.1624265	378
guanadrel	6.1528387	379
vincristine	6.146365	380
bufalin	6.141159	381
sparfloxacin	6.1371818	382
solanine	6.133134	383
cyclopamine	6.132392	384
digitoxigenin	6.1316457	385
fluticasone	6.1276445	386
Tiopronin (Thiola)	6.121499	387
Fumagillin	6.116757	388
Bardoxolone methyl	6.116632	389
Vincristine	6.1156445	390
methyl-testosterone	6.113766	391
alitretinoin	6.113429	392
Estradiol Cypionate	6.10795	393
Medroxyprogesterone acetate	6.1010065	394
Pimecrolimus	6.098155	395
gossypol	6.0963798	396
lylamine	6.0951385	397
Cinacalcet	6.093815	398
Exemestane	6.0934534	399
taurocholic-acid	6.0923543	400
Methylprednisolone	6.0861664	401
nisoldipine	6.0851755	402
Loteprednol etabonate	6.0814786	403
Adapalene sodium salt	6.080652	404
josamycin	6.0793257	405
pazufloxacin	6.0775633	406

guanethidine	6.068673	407
Streptomycin sulfate	6.062334	408
withaferin-a	6.051359	409
bulleyaconitine-a	6.0419555	410
isotretinoin	6.0366096	411
bafilomycin	6.0363913	412
levonorgestrel	6.0357857	413
Doripenem Hydrate	6.031625	414
sirolimus	6.029824	415
Rapamycin (Sirolimus)	6.0287523	416
Vecuronium Bromide	6.027317	417
nandrolone	6.02594	418
Simvastatin (Zocor)	6.017988	419
cortisone	6.0165644	420
lobelanidine	6.0161605	421
avrainvillamide-analog-6	6.0152135	422
Rimantadine Hydrochloride	6.010051	423
desoxycortone	6.0066614	424
levopropoxyphene	6.006448	425
Orphenadrine Citrate	6.004222	426
lasalocid	5.9955397	427
podophyllotoxin	5.99251	428
alclometasone	5.9923515	429
Ritodrine HCl	5.989374	430
spectinomycin	5.9849825	431
digoxigenin	5.979947	432
Ticarcillin sodium	5.9795485	433
tacrolimus	5.9783025	434
Lacidipine	5.976687	435
Tofacitinib (CP-690550) Citrate	5.972232	436
Paromomycin Sulfate	5.969888	437
Retapamulin	5.964547	438
butalbital	5.960429	439
Troglitazone	5.9602275	440
retinol	5.9590898	441
Cimetidine	5.957156	442
Procarbazine HCl	5.955502	443
Cortisone acetate	5.952636	444
Enoxolone	5.950199	445
Fluvastatin Sodium	5.947323	446
methandriol	5.944113	447
geldanamycin	5.942437	448
diflorasone	5.9411592	449
talampicillin	5.940233	450
nizatidine	5.9394045	451
Ethinyl Estradiol	5.938411	452
Sertraline HCl	5.937492	453
Remdesivir	5.9361377	454
medroxyprogesterone	5.931691	455
epothilone-a	5.929657	456
rimantadine	5.9284234	457

Verteporfin	5.927667	458
Ciclesonide	5.9276342	459
avrainvillamide-analog-2	5.923509	460
salmeterol	5.922088	461
prima-1-met	5.921732	462
methoxychlor	5.919485	463
Acarbose	5.914662	464
Dibucaine (Cinchocaine) HCl	5.908393	465
Docetaxel Trihydrate	5.9053497	466
tazobactam	5.904004	467
Pentoxyverine Citrate	5.9021606	468
dizocilpine	5.900499	469
nomilin	5.8956347	470
trolox	5.8950605	471
ingenol	5.8909154	472
timosaponin	5.8897133	473
Romidepsin (FK228, depsipeptide)	5.8894844	474
Alprostadil	5.8884325	475
lacidipine	5.8870907	476
halometasone	5.886361	477
deforolimus	5.8856087	478
montelukast	5.882566	479
Penicillin G Sodium	5.878317	480
Flumequine	5.8766413	481
hyperforin	5.8737726	482
oligomycin-c	5.868681	483
Etretinate	5.8641815	484
garcinol	5.861971	485
Prednisone	5.857891	486
ouabain	5.854896	487
vinblastine	5.8524256	488
Neomycin sulfate	5.841934	489
retinyl-acetate	5.841015	490
digoxin	5.8382425	491
dehydrocholic-acid	5.836096	492
Afuresertib	5.835397	493
Quinidine	5.8310905	494
S/GSK1349572	5.8247423	495
eserine	5.8125935	496
Decitabine (NSC127716, 5AZA-CdR)	5.8122797	497
megestrol-acetate	5.810218	498
nicergoline	5.808894	499
maraviroc	5.807028	500
trans-bis-q	5.801016	501
Lopinavir	5.795868	502
hexetidine	5.794654	503
nomegestrol	5.794485	504
R788 disodium	5.794012	505
pancuronium	5.7931232	506
Vinorelbine	5.792449	507
dirithromycin	5.792282	508

rifaximin	5.791498	509
torin-1	5.7798376	510
fluvastatin	5.777401	511
Amantadine HCl	5.773511	512
iodixanol	5.7733383	513
Bedaquiline fumarate	5.772065	514
flurandrenolide	5.7718253	515
totaralolal	5.7698774	516
LY2228820	5.768916	517
lanatoside-c	5.7634716	518
sericetin	5.761522	519
Tamoxifen Citrate	5.7595563	520
treprostinil	5.75502	521
estrone	5.7527046	522
Marimastat	5.74866	523
cyproterone	5.7482524	524
Fostamatinib (R788)	5.7464004	525
Fusidic Acid (sodium salt)	5.74244	526
verteporfin	5.73695	527
Ketorolac tromethamine salt	5.7361298	528
ebelactone-b	5.735753	529
Nizatidine	5.727047	530
Ibuprofen	5.72146	531
dithiothreitol	5.7195015	532
dehydroisoandosterone	5.7194805	533
flumetasone	5.7168503	534
Asunaprevir (BMS-650032)	5.7161064	535
Simeprevir	5.714564	536
avrainvillamide-analog-1	5.714485	537
flumethasone	5.7123957	538
tetrabenazine	5.708124	539
avrainvillamide-analog-4	5.707468	540
pyridine-2-aldoxime	5.7071257	541
Toremifene Citrate	5.7058773	542
spironolactone	5.7049747	543
heliomycin	5.703809	544
cerivastatin	5.7029734	545
dalcetrapib	5.7014723	546
rifampicin	5.699478	547
homoharringtonine	5.698909	548
CO-1686 (AVL-301)	5.6985903	549
ergocornine	5.698161	550
6-hydroxytropinone	5.6926947	551
Rifaximin (Xifaxan)	5.691886	552
nomegestrol-acetate	5.689867	553
Ac-Leu-Leu-Nle-CHO	5.6888785	554
Oxeladin Citrate	5.686765	555
ticarcillin	5.685827	556
Hyoscyamine	5.685819	557
SPB02137	5.680973	558
maprotiline	5.680134	559

depudecin	5.6772914	560
mometasone	5.6771245	561
ionomycin	5.676915	562
Prednisolone	5.674308	563
Cobimetinib	5.672736	564
Maraviroc	5.671544	565
ceftazidime	5.6713824	566
Ethynodiol diacetate	5.6704006	567
Nintedanib (BIBF 1120)	5.6654353	568
Deflazacort	5.665292	569
capsaicin	5.6621385	570
deoxycholic-acid	5.660894	571
fluocinolone	5.6607456	572
avicin-g	5.660124	573
megestrol	5.6583138	574
VX-222 (VCH-222, Lomibuvir)	5.652941	575
Vinpocetine	5.6526155	576
Choline Fenofibrate	5.651684	577
Alverine Citrate	5.6516023	578
1,2,3,4,5,6-hexabromocyclohexane	5.650468	579
aztreonam	5.646886	580
fostamatinib	5.6466694	581
Risedronate Sodium	5.6466355	582
Mometasone furoate	5.644803	583
Moxifloxacin HCl	5.6437845	584
Fluocinolone Acetonide	5.6432343	585
Ropinirole HCl	5.6430883	586
flavokavain-b	5.642605	587
usnic-acid	5.6367536	588
Docetaxel	5.6366677	589
Clomiphene citrate	5.6365776	590
4-hydroxyretinoic-acid	5.6339912	591
Carbenoxolone disodium	5.633521	592
Yohimbine Hydrochloride	5.626602	593
corticosterone	5.626585	594
Urapidil HCl	5.626277	595
carbidopa	5.626156	596
Cefoxitin (sodium salt)	5.6235886	597
androstenol	5.6229706	598
Valsartan	5.622262	599
Mosapride Citrate	5.620631	600
Dexamethasone (DHAP)	5.618307	601
amoxicillin	5.6161222	602
alexidine	5.615691	603
apramycin	5.61376	604
clocortolone	5.6131983	605
ibuprofen-(S)	5.6118317	606
Medroxyprogesterone	5.611755	607
bephenium	5.6093664	608
temsirolimus	5.6070538	609
prednisolone	5.6056905	610

penbutolol	5.605157	611
cefmetazole	5.6045837	612
noretynodrel	5.6026583	613
Carboplatin	5.60177	614
Methenamine	5.6002283	615
Everolimus (RAD001)	5.599532	616
propylthiouracil	5.599471	617
methylprednisolone	5.5946617	618
docetaxel	5.5945277	619
fluphenazine	5.594107	620
NAN-190	5.5934486	621
prostaglandin-a1	5.593418	622
MLN9708	5.591915	623
everolimus	5.5913815	624
Bindarit	5.590049	625
chlormadinone	5.5896235	626
Latrepirdine	5.587262	627
fenretinide	5.5865707	628
acepromazine	5.585184	629
Ceftazidime	5.584017	630
benzethonium	5.582164	631
Progesterone	5.5819163	632
scopolamine-n-oxide	5.581706	633
deflazacort	5.5806646	634
ICI-162846	5.580368	635
Miltefosine	5.5763264	636
Probucol	5.57564	637
hydroxychloroquine	5.5756235	638
betaescin	5.575409	639
Dabrafenib Mesylate (GSK-2118436)	5.5743303	640
zosuquidar	5.571138	641
medrysone	5.568925	642
tubocurarine	5.5685043	643
tetracycline	5.56587	644
proscillaridin-a	5.565386	645
Licofelone	5.563629	646
Domiphen Bromide	5.5612116	647
chlorzoxazone	5.5602956	648
dropropizine	5.5598764	649
flupentixol	5.5597258	650
z-leu3-VS	5.5571556	651
thonzonium	5.556145	652
ethinylestradiol	5.5555325	653
ungerine	5.553913	654
terreic-acid-(-)	5.5539083	655
Noradrenaline bitartrate monohydrate	5.553747	656
Altrenogest	5.55151	657
Pergolide mesylate	5.5512123	658
pirarubicin	5.5511293	659
tributyltin	5.5507336	660
eseroline	5.549294	661

5-methoxy-alpha-methyltryptamine	5.549259	662
acetohydroxamic-acid	5.548605	663
Temsirolimus	5.548258	664
propidium	5.546954	665
amiprilose	5.5461855	666
tropanyl-3,5-dimethylbenzoate	5.5449038	667
Sulbactam	5.544267	668
pentoxifylline	5.543972	669
Amoxicillin trihydrate	5.5428796	670
sulbactam	5.5426893	671
amphotericin-b	5.541004	672
tyloxapol	5.5360494	673
Oseltamivir phosphate	5.5357084	674
vecuronium	5.5348034	675
Benzethonium Chloride	5.5347805	676
Clofibrate	5.5341873	677
pivampicillin	5.534035	678
Aliskiren Hemifumarate	5.5334673	679
Sivelestat (ONO-5046)	5.5309577	680
kavain	5.5307755	681
Terbinafine	5.530339	682
etifenin	5.527634	683
physostigmine	5.5272646	684
ethynodiol	5.527055	685
selinidin	5.5270176	686
loteprednol	5.525282	687
oxalomalic-acid	5.5251474	688
Sodium salicylate	5.5227785	689
Irinotecan HCl Trihydrate	5.5223503	690
tridihexethyl	5.5221834	691
Vitamin A Acetate	5.520132	692
Tazarotene	5.519856	693
Telaprevir (VX-950)	5.5186377	694
Fingolimod (FTY720)	5.5178666	695
Olopatadine HCl	5.5166245	696
exemestane	5.515818	697
sn-Glycero-3-phosphocholine	5.5146375	698
3-matida	5.5133133	699
Estropipate	5.512048	700
Fosbretabulin (Combretastatin A4 Phosphate (CA4P)) Disodium	5.511654	701
bretylium	5.511636	702
heptaminol	5.5093555	703
Tranylcypromine hydrochloride	5.507223	704
Dehydroepiandrosterone (DHEA)	5.506337	705
prostaglandin	5.5055914	706
methyl-benzethonium	5.5052605	707
testosterone	5.504959	708
Crenolanib (CP-868596)	5.504266	709
Edrophonium (chloride)	5.5036945	710
Dicyclomine HCl	5.501996	711
sonidegib	5.501157	712

Amoxicillin	5.49992	713
Salbutamol Sulfate	5.4988422	714
gallamine	5.498198	715
Methscopolamine	5.4945383	716
Citric acid	5.493128	717
clobetasol	5.4919157	718
quiflapon	5.491049	719
GSK1349572 sodiuM salt	5.490346	720
Verapamil HCl	5.490202	721
Penciclovir	5.4885826	722
alfaxalone	5.4881325	723
methyl-dopate	5.4873114	724
Abacavir sulfate	5.4870234	725
Azlocillin sodium salt	5.4855433	726
Thio-TEPA	5.484583	727
n-nitrosodiethylamine	5.4827194	728
vinpocetine	5.4825335	729
securinine	5.482277	730
Carbidopa	5.482251	731
Tebipenempivoxil	5.481693	732
lisuride	5.4815855	733
Flavoxate hydrochloride	5.4812794	734
voriconazole	5.479645	735
nitrendipine	5.4784093	736
ENMD-2076	5.478066	737
CAM-9-021	5.4776745	738
Eflornithine hydrochloride monohydrate	5.4764442	739
Sodium Tauroursodeoxycholate (TUDC)	5.4763184	740
diosmin	5.4758964	741
tremulacin	5.4758453	742
meglumine	5.4755483	743
Mepyramine maleate	5.474492	744
Climbazole	5.4735556	745
tosedostat	5.472791	746
methylatropine	5.472761	747
Cyclophosphamide	5.472342	748
mepyramine	5.47213	749
Fluconazole	5.471937	750
pempidine	5.4717436	751
Aminothiazole	5.470916	752
nicardipine	5.4707375	753
calcifediol	5.4700537	754
acetylcholine	5.469963	755
luteolin	5.469922	756
sarmentogenin	5.469631	757
Noscapine HCl	5.4688263	758
stavudine	5.467956	759
Retinyl (Vitamin A) Palmitate	5.4676	760
Efinaconazole	5.4674296	761
allantoin	5.467189	762
cytochalasin-b	5.4665203	763

metacycline	5.464835	764
hippeastrine	5.463625	765
Isosorbide	5.463394	766
Anidulafungin	5.463252	767
Meptazinol HCl	5.4631786	768
rottlerin	5.462911	769
flunixin	5.462595	770
harpagoside	5.461049	771
Gallamine Triethiodide	5.4610457	772
Acetylcholine Chloride	5.4610195	773
phorbol-myristate-acetate	5.4609613	774
Clenbuterol (hydrochloride)	5.460795	775
clenbuterol	5.4605017	776
ABT-263 (Navitoclax)	5.4602127	777
ethisterone	5.4587874	778
pinacidil	5.4587145	779
saquinavir	5.4574566	780
Ampicillin	5.4558105	781
Doxylamine Succinate	5.4553404	782
methoprene-acid	5.454712	783
Entrectinib	5.4543486	784
imiquimod	5.45352	785
Gemfibrozil	5.453455	786
Cefoperazone	5.4533086	787
Bephenium Hydroxynaphthoate	5.452413	788
Metronidazole	5.4523315	789
TTNPB	5.4521146	790
LY335979 (Zosuquidar 3HCL)	5.451621	791
Nortriptyline (hydrochloride)	5.4515953	792
Sunitinib	5.451351	793
rimexolone	5.4508457	794
Tianeptine sodium	5.4502077	795
Gadodiamide	5.4495015	796
tretinoin	5.448734	797
proxymetacaine	5.4486876	798
SANT-1	5.448498	799
Lamivudine	5.447373	800
palonosetron	5.4470778	801
tanespimycin	5.447029	802
tramadol	5.446028	803
cilastatin	5.4459066	804
Scopine	5.445369	805
phenothiazine	5.445287	806
Alendronate	5.444802	807
glucosamine	5.444296	808
glipizide	5.4442234	809
L-Glutathione Reduced	5.4439955	810
pentetic-acid	5.4439516	811
phylloquinone	5.443259	812
Clofazimine	5.4430447	813
Aztreonam	5.44276	814

lithocholic-acid	5.441556	815
Otilonium Bromide	5.44005	816
licochalcone-a	5.439522	817
dolasetron	5.4392776	818
Valproic acid sodium salt (Sodium valproate)	5.4379253	819
alvocidib	5.4379206	820
Donepezil HCl	5.437769	821
Duloxetine HCl	5.43771	822
chloroform	5.437645	823
Terbutaline Sulfate	5.437483	824
ramifenazone	5.436861	825
Acitretin	5.435467	826
Betaine	5.435461	827
camptothecin	5.435385	828
terguride	5.4339437	829
C2-dihydroceramide	5.4337993	830
blebbistatin	5.4328814	831
Pefloxacin Mesylate	5.4325695	832
Proparacaine HCl	5.431674	833
Uracil	5.431284	834
Sotrastaurin (AEB071)	5.4311504	835
calcitriol	5.4300756	836
orphenadrine	5.429818	837
Manidipine	5.429663	838
ibandronic-acid	5.429225	839
Sertaconazole nitrate	5.4291525	840
ipriflavone	5.429117	841
meropenem	5.428893	842
sunitinib	5.428426	843
Bexarotene	5.4273043	844
Bedaquiline	5.4272947	845
Pioglitazone HCl	5.4260693	846
nintedanib	5.4253483	847
antazoline	5.4251175	848
tylosin	5.4249306	849
5-Aminolevulinic acid HCl	5.4240437	850
Ulipristal acetate	5.4238305	851
avicin-d	5.42376	852
arachidonyl-trifluoro-methane	5.423744	853
Trometamol	5.422992	854
Lomitapide	5.421996	855
Pamidronate Disodium	5.42186	856
methylergometrine	5.4217463	857
myriocin	5.42089	858
Ivabradine HCl	5.4208345	859
Terfenadine	5.4205837	860
disulfiram	5.420162	861
pregnenolone	5.4198084	862
Emtricitabine	5.419773	863
argatroban	5.4195423	864
BIBR-1048	5.4183316	865

clobutinol	5.4182377	866
pirinixic-acid	5.4178834	867
oxetacaine	5.417858	868
metrifonate	5.417612	869
Flumethasone	5.416378	870
cycloserine-(d)	5.4161215	871
nitrocaramiphen	5.4160957	872
mepenzolate	5.4160547	873
pralidoxime	5.415449	874
tiapride	5.41532	875
cefepime	5.414674	876
procainamide	5.413307	877
bethanechol	5.412344	878
SKF 525A (hydrochloride)	5.411575	879
Atomoxetine HCl	5.4110975	880
verapamil	5.410021	881
thioperamide	5.409474	882
melphalan	5.4091225	883
Guanabenz Acetate	5.408954	884
Dacarbazine	5.408947	885
butyrolactone	5.408886	886
clofilium	5.408181	887
Fenofibric acid	5.4079704	888
endo-IWR-1	5.407908	889
z-guggulsterone	5.4078093	890
Mizoribine	5.406399	891
Tipiracil hydrochloride	5.406229	892
Cisatracurium Besylate	5.4059477	893
Elacridar	5.4054613	894
Epinephrine HCl	5.405211	895
procaine	5.4050117	896
Oxytetracycline Dihydrate	5.4047594	897
Tolazoline HCl	5.4046993	898
LFM-A12	5.403922	899
kanamycin	5.4038973	900
RDR-01440SC	5.4037066	901
Gabapentin HCl	5.403361	902
Riluzole	5.403084	903
Montelukast Sodium	5.4028273	904
Nicardipine HCl	5.402627	905
(+)-Ketoconazole	5.4024134	906
Lithium Citrate	5.402114	907
etretinate	5.402049	908
Clemastine Fumarate	5.401662	909
valsartan	5.401585	910
10-hydroxycamptothecin	5.4014845	911
Darunavir	5.4012165	912
meclofenamic-acid	5.400834	913
flavoxate	5.400626	914
Irinotecan	5.4004054	915
Bicalutamide	5.399973	916

2-Thiouracil	5.399302	917
simvastatin	5.3986645	918
edaravone	5.3985224	919
Procainamide HCl	5.3972178	920
tiletamine	5.3963966	921
manumycin-a	5.396309	922
Mesalamine	5.3952665	923
Clobetasol propionate	5.3951974	924
Histamine 2HCl	5.3950024	925
Crystal Violet	5.394907	926
nifedipine	5.39464	927
actinomycin-d	5.3933434	928
azlocillin	5.3932595	929
pivmecillinam	5.392861	930
terbinafine	5.392843	931
dydrogesterone	5.391882	932
trimethadione	5.3916535	933
Fudosteine	5.3905945	934
etidronic-acid	5.390315	935
Avobenzone	5.3896084	936
10-DEBC	5.3893595	937
Benidipine HCl	5.3893423	938
Beclomethasone dipropionate	5.3892336	939
metitepine	5.3891883	940
Diphemanil Methylsulfate	5.388812	941
Bemegride	5.3885064	942
KDM-103	5.388482	943
benactyzine	5.388275	944
gabapentin	5.3869066	945
Sildenafil Citrate	5.386892	946
acyclovir	5.386422	947
lynestrenol	5.3861933	948
Naphazoline HCl	5.3860264	949
Anastrozole	5.3859468	950
Cyclophosphamide monohydrate	5.384942	951
danazol	5.384446	952
meclofenoxate	5.3842597	953
Oxybuprocaine HCl	5.3841224	954
Decamethonium Bromide	5.3841133	955
GBR-12783	5.3826203	956
5-methylhydantoin	5.3823547	957
flunarizine	5.3822346	958
scandenin	5.3814588	959
Dinaciclib (SCH727965)	5.3813953	960
Ampicillin Trihydrate	5.3812857	961
lofexidine	5.380467	962
quinidine	5.3802495	963
Sulbactam sodium	5.37847	964
MLN2238	5.378251	965
Gimeracil	5.378173	966
Adiphenine HCl	5.3768396	967

Guanfacine hydrochloride	5.3767614	968
meprylcaine	5.3762746	969
ampicillin	5.376108	970
MEK1-2-inhibitor	5.37566	971
Fluoxetine HCl	5.375531	972
Alcaftadine	5.3750973	973
Tetrahydrozoline HCl	5.374732	974
tigecycline	5.374719	975
dactinomycin	5.3746543	976
naftopidil	5.3742776	977
Metformin HCl	5.374129	978
Danazol	5.374076	979
Sunitinib malate	5.3740597	980
Divalproex Sodium	5.37324	981
Cetirizine	5.3732033	982
Nedaplatin	5.372333	983
Cefaclor	5.372045	984
cycloserine	5.371318	985
Doxepin (hydrochloride)	5.3706627	986
propoxycaine	5.3705673	987
morantel	5.3704667	988
Maprotiline HCl	5.370097	989
dantrolene	5.369723	990
methantheline	5.3696566	991
n-bromoacetyltryptamine	5.3686743	992
Adefovir Dipivoxil	5.3686457	993
ethoxyquin	5.368353	994
(+,-)-Octopamine HCl	5.368061	995
Tripelennamine HCl	5.3675466	996
Tizanidine HCl	5.3674874	997
indinavir	5.3669796	998
Mefloquine hydrochloride	5.366671	999
Betamethasone Dipropionate	5.3666687	1000
celiprolol	5.366475	1001
Cilomilast	5.3663173	1002
theobromine	5.36582	1003
fasudil	5.365758	1004
xylazine	5.3651133	1005
allopurinol	5.3646226	1006
Difluprednate	5.3644094	1007
cloperastine	5.364362	1008
epidepride	5.3643546	1009
norketamine	5.3637257	1010
Venlafaxine	5.3636227	1011
levocetirizine	5.36307	1012
pyrrolidine-dithiocarbamate	5.3621616	1013
hydroxyl	5.36185	1014
Tiotropium Bromide	5.3616223	1015
Cephalexin	5.361583	1016
Abacavir	5.361079	1017
Amodiaquine dihydrochloride dihydrate	5.360984	1018

BAX-channel-blocker	5.3609085	1019
Trospium chloride	5.3606195	1020
trap-101	5.3587637	1021
glutamine	5.3586817	1022
chloramphenicol	5.35823	1023
Losartan	5.3578367	1024
phenylpropanolamine	5.3573704	1025
idoxuridine	5.3573647	1026
urethane	5.357112	1027
moxonidine	5.3567104	1028
Guanidine HCl	5.356683	1029
apicidin	5.3557205	1030
iodophenpropit	5.3554983	1031
iodoacetic-acid	5.355341	1032
N-methyllidocaine-iodide	5.3553305	1033
oxytetracycline	5.355142	1034
nornicotine	5.3548923	1035
Ziprasidone HCl	5.354734	1036
Clonidine HCl	5.354678	1037
Moroxydine HCl	5.35408	1038
Ebastine	5.3540344	1039
BIRB 796 (Doramapimod)	5.353848	1040
carbon-tetrachloride	5.3534884	1041
fluoxymesterone	5.3528414	1042
Mechlorethamine HCl	5.3527727	1043
Ranolazine 2HCl	5.3527107	1044
chlorcyclizine	5.352705	1045
Diosimin	5.352537	1046
dicoumarol	5.3524923	1047
mevalonic-acid	5.352397	1048
meclizine	5.3519945	1049
Protoporphyrin IX	5.351856	1050
Prednisolone Acetate	5.3513556	1051
Ibandronate sodium	5.3509703	1052
Sodium Picosulfate	5.3508224	1053
Pimavanserin	5.350654	1054
KDM-096	5.3503647	1055
tryptophan	5.3501835	1056
Phenazopyridine HCl	5.3500385	1057
Pridinol Methanesulfonate	5.3496327	1058
acetamide	5.3491783	1059
Alendronate sodium	5.349099	1060
Triflurdine (Viroptic)	5.3486333	1061
Chlorhexidine HCl	5.3484607	1062
Sodium 4-Aminosalicylate	5.348138	1063
Vidarabine	5.347783	1064
opipramol	5.346829	1065
orlistat	5.3463764	1066
novobiocin	5.3458796	1067
Lidocaine	5.345397	1068
lomifylline	5.3453913	1069

ribostamycin	5.345339	1070
benproperine	5.3452363	1071
Cetirizine DiHCl	5.345224	1072
Methyldopa	5.345154	1073
Eprazinone 2HCl	5.345089	1074
Amonafide	5.344801	1075
Irbesartan	5.3447914	1076
mefexamide	5.3439665	1077
dacarbazine	5.343951	1078
meptazinol	5.343545	1079
etomoxir	5.3431997	1080
Ozagrel HCl	5.343198	1081
hydralazine	5.343109	1082
COL-3	5.3429804	1083
Sitafloxacin Hydrate	5.342666	1084
Bezafibrate	5.3426657	1085
Ospemifene	5.342218	1086
Vilazodone Hydrochloride	5.341762	1087
reversine	5.3413844	1088
Levosimendan	5.3413754	1089
pentoxyverine	5.3413467	1090
GBR-12935	5.340688	1091
zearalenone	5.3403664	1092
Regorafenib hydrochloride	5.3398895	1093
naphazoline	5.339781	1094
sulfaquinoxaline	5.3388886	1095
Cyproheptadine hydrochloride	5.338659	1096
Moguisteine	5.3386164	1097
cromoglicic-acid	5.3385105	1098
Dibucaine	5.33843	1099
cephalosporanic-acid	5.338402	1100
Ticagrelor	5.338214	1101
Semagacestat (LY450139)	5.3380523	1102
Amiloride HCl	5.3379726	1103
halofantrine	5.3377604	1104
Camostat Mesilate	5.337599	1105
Hydroxychloroquine Sulfate	5.3375025	1106
benfotiamine	5.3370323	1107
Meclofenamate Sodium	5.336439	1108
sisomicin	5.336364	1109
Cetrimonium Bromide (CTAB)	5.3360176	1110
clomifene	5.3358345	1111
oxyphenonium	5.3356123	1112
terbutaline	5.33428	1113
acetazolamide	5.3341055	1114
Diltiazem HCl	5.333701	1115
carbarsone	5.3334503	1116
GNF-2	5.333026	1117
Ranitidine	5.332963	1118
vanoxerine	5.332402	1119
IPI-145 (INK1197)	5.3321896	1120

Zoledronic Acid	5.331732	1121
Aclidinium Bromide	5.3314247	1122
1-monopalmitin	5.3313675	1123
mephentermine	5.3312864	1124
Topotecan HCl	5.3311167	1125
fipexide	5.3308477	1126
Succinylcholine Chloride Dihydrate	5.3307753	1127
Moxalactam (sodium salt)	5.3306966	1128
misoprostol	5.330618	1129
tripelennamine	5.3302054	1130
Arbutin	5.330158	1131
Auranofin	5.3299685	1132
Furaltadone HCl	5.3298626	1133
alendronic-acid	5.329548	1134
Rutin	5.329202	1135
levalbuterol	5.329069	1136
floxuridine	5.328376	1137
Dapoxetine HCl	5.3283625	1138
Dibutyryl-cAMP, sodium salt	5.3283377	1139
Phenylephrine HCl	5.3281555	1140
safinamide	5.327717	1141
GBR-13069	5.3268194	1142
AR-C133057XX	5.326806	1143
Argatroban	5.3267384	1144
L-Glutamine	5.326699	1145
tivantinib	5.326505	1146
isoflupredone-acetate	5.3264785	1147
alpha-methylserotonin	5.326103	1148
piperacillin	5.325861	1149
dihydroxyphenylglycine	5.325857	1150
inhibitor-BEC	5.3257294	1151
homosalate	5.3256745	1152
cinnarizine	5.3256497	1153
Buflomedil HCl	5.3256006	1154
Toltrazuril	5.325261	1155
Diclofenac Potassium	5.325136	1156
leucovorin	5.32485	1157
Buspirone HCl	5.3243923	1158
mifepristone	5.324338	1159
felamidin	5.3242197	1160
levcromakalim	5.32417	1161
Ziprasidone hydrochloride monohydrate	5.3240952	1162
vigabatrin	5.323799	1163
Tetracaine HCl	5.3230095	1164
AR-A014418	5.3229923	1165
Phenformin HCl	5.322969	1166
Praziquantel	5.3225656	1167
Olanzapine	5.3224716	1168
Desipramine hydrochloride	5.3224397	1169
ciprofloxacin	5.3222437	1170
Diclofenac Sodium	5.3220167	1171

salbutamol	5.3219275	1172
thioproperazine	5.321372	1173
butirosin	5.3211603	1174
Clomipramine HCl	5.3208447	1175
Rupatadine Fumarate	5.3207645	1176
formoterol	5.3204026	1177
xamoterol	5.3203926	1178
Penfluridol	5.320104	1179
Olsalazine Sodium	5.3199463	1180
isoflupredone	5.31986	1181
Plerixafor 8HCl (AMD3100 8HCl)	5.319785	1182
methyl-fasudil	5.319762	1183
Amitriptyline HCl	5.319409	1184
quinoclamine	5.319407	1185
QX-314	5.319108	1186
loxoprofen	5.318921	1187
PHTPP	5.3186717	1188
metaraminol	5.3183537	1189
hemicholinium-3	5.3181458	1190
Butenafine HCl	5.318083	1191
Dexamethasone acetate	5.3177233	1192
gliquidone	5.317709	1193
Fexofenadine HCl	5.3175015	1194
xaliproden	5.3169823	1195
VX-809	5.3166385	1196
Ibutilide Fumarate	5.3166294	1197
dacinostat	5.3165197	1198
norfenfluramine	5.3165016	1199
quinelorane	5.316496	1200
Dextrose (D-glucose)	5.316168	1201
aceclidine	5.3160176	1202
tranylcypromine	5.316002	1203
thiomersal	5.315074	1204
debrisoquine	5.314806	1205
toremifene	5.3145914	1206
Rifapentine	5.3144417	1207
GS-9973	5.314251	1208
nafcilline	5.3140306	1209
Ranolazine	5.3138742	1210
troxipide	5.3138065	1211
Cefsulodin (sodium salt)	5.3134613	1212
chromomycin-a3	5.313223	1213
Voriconazole	5.3127117	1214
bacitracin	5.312546	1215
Topotecan	5.3124323	1216
apigenin	5.3116736	1217
Diphenhydramine HCl	5.311638	1218
bacampicilline	5.3116236	1219
Pefloxacin Mesylate Dihydrate	5.311549	1220
tyrphostin-51	5.3112745	1221
Sulconazole Nitrate	5.3109856	1222

linoleamide	5.3108826	1223
Cinacalcet HCl	5.310804	1224
Desvenlafaxine	5.3105206	1225
Nitisinone	5.310482	1226
thapsigargin	5.310444	1227
fluorometholone	5.3102756	1228
Enrofloxacin	5.3102508	1229
scriptaid	5.310231	1230
Piperacillin Sodium	5.310194	1231
Phenytoin sodium	5.309947	1232
fluvoxamine	5.309557	1233
Varenicline Tartrate	5.3094172	1234
Ofloxacin	5.309359	1235
dimethylnitrosamine	5.30935	1236
Aripiprazole	5.309247	1237
tetradecylthioacetic-acid	5.3091855	1238
midostaurin	5.3088856	1239
ibutilide	5.308689	1240
moxifloxacin	5.3085003	1241
Florfenicol	5.3084784	1242
l-buthionine-sulfoximine	5.3084517	1243
Pomalidomide (CC-4047)	5.3083906	1244
Cefonicid (sodium salt)	5.30828	1245
ellipticine	5.3080807	1246
Defactinib	5.308017	1247
Ifenprodil Tartrate	5.307931	1248
Almotriptan Malate	5.307909	1249
Empagliflozin (BI 10773)	5.307555	1250
lofepramine	5.307501	1251
Gastrodin	5.30737	1252
Cinepazide maleate	5.3073483	1253
buthionine-sulfoximine	5.3068514	1254
captopril	5.306291	1255
demeclocycline	5.3059187	1256
Riociguat	5.3058715	1257
neomycin	5.305854	1258
levodopa	5.3056765	1259
famotidine	5.3056517	1260
Pimozide	5.305627	1261
Chlorpromazine HCl	5.305443	1262
cilomilast	5.305214	1263
dimaprit	5.3050156	1264
hycanthone	5.3043156	1265
fludrocortisone	5.304069	1266
reserpine	5.303875	1267
Tideglusib	5.303738	1268
phthalylsulfathiazole	5.3036776	1269
ryuvidine	5.303383	1270
OBAA	5.3033257	1271
oxantel	5.3032646	1272
Motolimod (VTX-2337)	5.302926	1273

aprepitant	5.3026648	1274
glycodeoxycholic-acid	5.3025064	1275
Gabexate mesylate	5.302393	1276
cladribine	5.3023024	1277
Linifanib (ABT-869)	5.3020673	1278
Regorafenib monohydrate	5.3018513	1279
phentolamine	5.301846	1280
Ribavirin	5.3018265	1281
Antazoline HCl	5.3017488	1282
lithium-chloride	5.301652	1283
Kanamycin Sulfate	5.3015575	1284
tetrachloroethylene	5.3011	1285
clofazimine	5.301023	1286
Pentostatin	5.3009553	1287
rivaroxaban	5.3009458	1288
adiphenine	5.300905	1289
tetracaine	5.3006363	1290
norethindrone	5.3004313	1291
Paclitaxel (Taxol)	5.3002987	1292
remacemide	5.3001904	1293
isoreserpine	5.2999063	1294
latrepirdine	5.2997746	1295
Voglibose	5.299664	1296
chlortetracycline	5.299634	1297
sulfadimidine	5.299597	1298
Pyrantel Pamoate	5.299567	1299
MDM2-inhibitor	5.299428	1300
Cefadroxil (hydrate)	5.299291	1301
itdac-1	5.2992754	1302
Ifosfamide	5.2990255	1303
paclitaxel	5.2990017	1304
Scopolamine hydrobromide	5.298705	1305
Bepotastine Besilate	5.298691	1306
cortisol	5.298505	1307
sulfathiazole	5.2983646	1308
CYT387	5.2982388	1309
etofenamate	5.298153	1310
acadesine	5.298148	1311
Carbamoylcholine chloride	5.2980833	1312
Cyclobenzaprine HCl	5.297426	1313
2-fluoropalmitic-acid	5.2973843	1314
ASN-05257430	5.2973814	1315
mycophenolate-mofetil	5.296955	1316
Felbamate	5.296333	1317
tyrphostin-AG-1288	5.296283	1318
pemoline	5.2962456	1319
Clozapine	5.29613	1320
Propafenone HCl	5.295994	1321
SPB02303	5.2959414	1322
Cidofovir	5.295909	1323
Tadalafil	5.2958803	1324

MK-4827	5.2958636	1325
Imipramine (hydrochloride)	5.29576	1326
Primidone	5.2953076	1327
nateglinide	5.2950907	1328
dexamethasone	5.295003	1329
Mezlocillin Sodium	5.2947035	1330
trehalose	5.2943983	1331
Carmustine	5.2941294	1332
depomedrol	5.294003	1333
5-hydroxytryptophan	5.293997	1334
phenazone	5.2939425	1335
dimethadione	5.293812	1336
doxapram	5.2937913	1337
ketoconazole	5.2937	1338
dimenhydrinate	5.293667	1339
Linagliptin (BI-1356)	5.2933035	1340
calcipotriol	5.293086	1341
Mecarbinate	5.2930384	1342
niacin	5.292935	1343
tyrphostin-AG-112	5.2923036	1344
pyrantel	5.292242	1345
lorglumide	5.2922316	1346
Adrenalone HCl	5.2920456	1347
Nifedipine	5.2919493	1348
prednicarbate	5.2917194	1349
eucatropine	5.291671	1350
polydatin	5.291664	1351
famprofazone	5.291644	1352
terazosin	5.2914305	1353
Cyclandelate	5.290902	1354
tegaserod	5.2908354	1355
Doxycycline hyclate	5.29076	1356
synephrine	5.29071	1357
idarubicin	5.2905235	1358
Mitotane (Lsodren)	5.290301	1359
Levobupivacaine HCl	5.290116	1360
Trichlormethiazide	5.290002	1361
zebularine	5.2896404	1362
Clindamycin Phosphate	5.289074	1363
perindopril	5.28875	1364
niguldipine	5.288739	1365
beta-alanine	5.288645	1366
Sodium 4-amiropparaty Hyalrate	5.288512	1367
lysergol	5.2882133	1368
Silodosin	5.288167	1369
Diphenylpyraline HCl	5.288116	1370
Tiagabine	5.2881002	1371
dyclonine	5.2878904	1372
Palonosetron HCl	5.287591	1373
K-115	5.2874618	1374
pentylenetetrazol	5.2871	1375

zolpidem	5.286951	1376
Tasisulam	5.286627	1377
Sulfamethizole	5.2864923	1378
prostaglandin-b2	5.2863855	1379
cyclizine	5.2862034	1380
iopamidol	5.2861934	1381
fenobam	5.2861047	1382
Budesonide	5.2860155	1383
relcovaptan	5.285987	1384
Naftifine HCl	5.2859793	1385
Anagrelide HCl	5.2857313	1386
meclocycline	5.2857003	1387
diphenoxylate	5.285654	1388
micropenin	5.2852902	1389
sertindole	5.2852845	1390
Erythromycin	5.2852364	1391
Pranoprofen	5.285083	1392
PMSF	5.2849684	1393
evoxine	5.2849636	1394
budesonide	5.284837	1395
atovaquone	5.28477	1396
rivastigmine	5.284738	1397
nalidixic-acid	5.284597	1398
ethosuximide	5.284422	1399
Amprolium HCl	5.2843313	1400
hyperoside	5.284276	1401
finasteride	5.284226	1402
isoquercetin	5.284006	1403
3,6-dimethoxyflavone	5.283985	1404
gliclazide	5.2839603	1405
Sodium Nitrite	5.283764	1406
trifluridine	5.2834835	1407
Primaquine Diphosphate	5.2833576	1408
paromomycin	5.2832556	1409
Cetylpyridinium Chloride	5.283081	1410
glibenclamide	5.283079	1411
Amlodipine Besylate	5.283041	1412
zardaverine	5.283003	1413
Gly-Gly-PALO	5.282961	1414
Sulfamethoxazole	5.2829294	1415
Entacapone	5.282903	1416
hyoscyamine	5.282628	1417
glutamyldopamine	5.282403	1418
BMS-708163 (Avagacestat)	5.2821503	1419
AF-DX-116	5.281953	1420
tranilast	5.281947	1421
Lomerizine HCl	5.28188	1422
topotecan	5.281663	1423
losartan	5.2816257	1424
Alosetron Hydrochloride	5.281617	1425
febuxostat	5.281598	1426

Haloperidol hydrochloride	5.2813435	1427
Ambroxol HCl	5.281203	1428
importazole	5.2810683	1429
curcumin	5.2810316	1430
vesamicol	5.2810087	1431
FCCP	5.280735	1432
Baricitinib phosphate	5.2806225	1433
gemcitabine	5.280588	1434
Arecoline hydrobromide	5.2804394	1435
butylscopolammonium	5.280143	1436
pelitinib	5.279994	1437
Isavuconazole	5.27994	1438
isosalsoline	5.2798376	1439
BYL-719	5.2797003	1440
mesna	5.2796116	1441
Pidotimod	5.279552	1442
Pizotifen	5.279543	1443
(S)-Flurbiprofen	5.2793446	1444
ICI-199441	5.279253	1445
sulforaphane	5.2791204	1446
hexamethonium	5.2790766	1447
rimcazole	5.2789783	1448
Allylthiourea	5.2784905	1449
imperatorin	5.2781205	1450
Zonisamide	5.277675	1451
labetalol	5.277533	1452
Fenoprofen calcium hydrate	5.2774067	1453
Zaltoprofen	5.277383	1454
Alogliptin Benzoate	5.2770767	1455
rifapentine	5.2769537	1456
erastin	5.2767315	1457
noreleagnine	5.2766895	1458
diltiazem	5.2765923	1459
PRL-3-inhibitor-I	5.276461	1460
Nilvadipine	5.2764506	1461
Rivastigmine	5.276438	1462
phosphodiesterase-V-inhibitor-II	5.27639	1463
MK-5172	5.2763233	1464
sparfosic-acid	5.276183	1465
Valproic acid	5.2756224	1466
fulvestrant	5.275419	1467
fluticasone-propionate	5.2752438	1468
BI-78D3	5.275178	1469
BO2-inhibits-RAD51	5.2751036	1470
Terbinafine HCl	5.274871	1471
nomifensine	5.274834	1472
Alvelestat	5.2747746	1473
Miconazole Nitrate	5.2747655	1474
nadifloxacin	5.2745924	1475
tribenoside	5.274493	1476
Quetiapine Fumarate	5.2743692	1477

tremorine	5.274001	1478
Cysteamine HCl	5.273923	1479
enalapril	5.2739124	1480
Sodium Phenylbutyrate	5.273883	1481
norethisterone	5.2732825	1482
Sorafenib Tosylate	5.2725677	1483
Ribostamycin Sulfate	5.272251	1484
phenethyl-isothiocyanate	5.2720413	1485
fosinopril	5.271042	1486
IQ1	5.270895	1487
gabexate	5.270838	1488
Sulfisoxazole	5.270643	1489
Napabucasin	5.2706327	1490
Rosiglitazone maleate	5.2706127	1491
chloropyramine	5.270524	1492
progesterone	5.270288	1493
M2-PK-activator	5.270275	1494
SJB-AF-2	5.2701025	1495
alpha-linolenic-acid	5.2698917	1496
Alfuzosin HCl	5.2696466	1497
3-methyl-GABA	5.2695045	1498
isosorbide	5.26948	1499
Oxiracetam	5.269248	1500
ampyrone	5.2692347	1501
mibefradil	5.2687273	1502
Epinastine HCl	5.2686377	1503
enalaprilat	5.268242	1504
paroxetine	5.267898	1505
Pilocarpine HCl	5.267829	1506
Famotidine	5.2677784	1507
Hydralazine HCl	5.267578	1508
fenofibrate	5.2674646	1509
Rotundine	5.2674255	1510
Estrone	5.267392	1511
Betamethasone	5.267271	1512
apomorphine	5.266965	1513
foliosidine	5.2667837	1514
tolcapone	5.2667255	1515
nutlin-3	5.266701	1516
eriodictyol	5.266588	1517
azithromycin	5.2665453	1518
Letrozole	5.2665143	1519
CTPB	5.266478	1520
KB-R7943	5.2664404	1521
nefopam	5.266132	1522
Etravirine (TMC125)	5.2659607	1523
Rabeprazole sodium	5.2658315	1524
tolazamide	5.265745	1525
Ketotifen Fumarate	5.265338	1526
Mycophenolate Mofetil	5.265232	1527
Sitagliptin phosphate monohydrate	5.2651944	1528

Mirabegron (YM178)	5.264748	1529
Tetracycline Hydrochloride	5.2647343	1530
thenoyltrifluoroacetone	5.2646437	1531
PHA-793887	5.2646275	1532
Busulfan	5.2643347	1533
Uridine	5.2641444	1534
Aminophylline	5.2641277	1535
lumicolchicine	5.2641253	1536
GMX-1778	5.263813	1537
procyclidine	5.2637644	1538
quercetagetin	5.263509	1539
fenpiverinium	5.2634254	1540
hydroxyzine	5.263172	1541
Dopamine HCl	5.2630787	1542
taurodeoxycholic-acid	5.2627163	1543
Phenylpiracetam	5.2627153	1544
methapyrilene	5.262541	1545
somatostatin	5.262512	1546
Choline Chloride	5.2624683	1547
Eprosartan Mesylate	5.2621517	1548
Glycopyrrolate	5.262149	1549
Ethacridine lactate monohydrate	5.262114	1550
Phenylbutazone	5.262083	1551
flurofamide	5.2620134	1552
Daptomycin	5.2618637	1553
Phenoxybenzamine HCl	5.2616825	1554
ketocholesterol	5.261672	1555
nisoxetine	5.2616625	1556
Telmisattan	5.2616463	1557
Flunarizine 2HCl	5.261213	1558
m-3M3FBS	5.260749	1559
gamma-linolenic-acid	5.2604914	1560
metformin	5.260424	1561
fluperlapine	5.2601857	1562
Brinzolamide	5.2601614	1563
Nitrofurazone	5.2599134	1564
Pizotifen Malate	5.2599072	1565
Loxapine Succinate	5.2596254	1566
Diphenidol HCl	5.259582	1567
Ropivacaine HCl	5.2594585	1568
sirtinol	5.2592425	1569
Cilostazol	5.2587967	1570
Oxacillin sodium monohydrate	5.2587795	1571
4-carboxy-3-hydroxyphenylglycine-(RS)	5.2583857	1572
cefotiam	5.2580285	1573
immepip	5.2579403	1574
Solifenacin succinate	5.257884	1575
cefalotin	5.257822	1576
Benzydamine HCl	5.257541	1577
monoethylhexylphthalate	5.257506	1578
oxolinic-acid	5.2574415	1579

atracurium	5.257306	1580
Zoxazolamine	5.25712	1581
Desvenlafaxine Succinate	5.257116	1582
hydroflumethiazide	5.2571087	1583
Milnacipran HCl	5.2570677	1584
lidoflazine	5.256985	1585
Isradipine (Dynacirc)	5.256775	1586
Bupropion hydrochloride	5.2563543	1587
Ticlopidine HCl	5.256241	1588
aurora-a-inhibitor-i	5.2561684	1589
alizapride	5.2560816	1590
tropisetron	5.2560487	1591
decitabine	5.256001	1592
cyclosporin-a	5.2559013	1593
cycloheximide	5.255805	1594
xanthinol	5.255499	1595
glycerol	5.255286	1596
ponalrestat	5.255213	1597
salsoline	5.255193	1598
mead-acid	5.2549934	1599
fluoropyruvate	5.2547812	1600
Deferoxamine mesylate	5.254678	1601
gangaleoidin	5.2545877	1602
Pheniramine Maleate	5.254572	1603
Closantel Sodium	5.254411	1604
Niflumic acid	5.2540436	1605
thiazolidinecarboxylic-acid	5.254039	1606
Sodium Monofluorophosphate	5.2538795	1607
etofylline	5.253772	1608
cytochalasin-d	5.2536793	1609
Nafcillin Sodium	5.2534657	1610
Benzocaine	5.2531557	1611
Aminoglutethimide	5.2531357	1612
Benserazide HCl	5.253004	1613
Quetiapine	5.252636	1614
Nefiracetam	5.2524014	1615
NAS-181	5.252396	1616
necrostatin-1	5.2522826	1617
CAM-9-027-3	5.2521753	1618
pimethixene	5.2520933	1619
azapropazone	5.2519445	1620
cyproheptadine	5.251875	1621
p-azido-PE-TFMPP	5.2516537	1622
deptropine	5.251581	1623
Anisodamine	5.2515516	1624
Metolazone	5.251277	1625
clavulanic-acid	5.251256	1626
butacaine	5.250806	1627
hydroquinidine	5.25074	1628
Sulfaguanidine	5.250301	1629
Regadenoson	5.2502737	1630

kinetin	5.2501135	1631
Indapamide	5.2501097	1632
flufenamic-acid	5.249957	1633
Terazosin HCl	5.2496924	1634
1-methylisoquinoline	5.2496223	1635
Tirofiban hydrochloride monohydrate	5.2496	1636
Ondansetron hydrochloride dihydrate	5.2494907	1637
Sulfameter	5.2491097	1638
desmethylclozapine	5.2490835	1639
Ganciclovir	5.2488036	1640
Teneligliptin hydrobromide	5.2481356	1641
epinephrine-(racemic)	5.247979	1642
melperone	5.247954	1643
Cromolyn sodium	5.2478046	1644
TH-302	5.247712	1645
Ezetimibe	5.2474747	1646
Pemetrexed disodium hemipenta hydrate	5.2472153	1647
Cepharanthine	5.2471294	1648
methylnorlichexanthone	5.246906	1649
hydroxytyrosol	5.2467923	1650
Orotic acid	5.246298	1651
J-104129	5.2462826	1652
Olmesartan medoxomil	5.2460747	1653
IRL-2500	5.246028	1654
diethylstilbestrol	5.2456756	1655
Fluticasone propionate	5.245652	1656
kinetin-riboside	5.245618	1657
Piperine	5.245391	1658
urapidil	5.244973	1659
methyl-hydantoin	5.244955	1660
pyroxamide	5.2447653	1661
Dexrazoxane HCl (ICRF-187, ADR-529)	5.24475	1662
Sulfanilamide	5.2445846	1663
Acetanilide	5.2442193	1664
Leflunomide	5.2442102	1665
moclobemide	5.2440987	1666
aminosalicylic-acid	5.24409	1667
hispidin	5.2440557	1668
benzathine	5.2437086	1669
silibinin	5.243683	1670
cloxacillin	5.2436113	1671
Xylazine HCl	5.243492	1672
olanzapine	5.2430754	1673
Hydroxyzine 2HCl	5.242853	1674
thiothixene	5.2424273	1675
cyclazosin	5.242345	1676
mupirocin	5.2421656	1677
pseudopelletierine	5.241944	1678
suberoyl-bis-hydroxamic-acid	5.2418327	1679
8-Methoxypsoralen	5.2415776	1680
TW-37	5.2415175	1681

lomefloxacin	5.241416	1682
Guaifensin	5.2413387	1683
daunorubicin	5.241308	1684
Scopolamine butylbromide	5.2411375	1685
probenecid	5.2406025	1686
Clofibric Acid	5.2401266	1687
piracetam	5.2398033	1688
Dovitinib (TKI-258, CHIR-258)	5.239665	1689
isradipine	5.2395864	1690
ivachtin	5.239479	1691
tropapride	5.2391744	1692
Naratriptan	5.2388716	1693
vitexin	5.238096	1694
Phentolamine Mesylate	5.237989	1695
Promethazine HCl	5.2379026	1696
ixazomib	5.237886	1697
anandamide	5.237676	1698
M-344	5.237652	1699
dexrazoxane	5.237606	1700
roxatidine-acetate	5.237562	1701
norcyclobenzaprine	5.237533	1702
GS-7340	5.2375216	1703
Tiratricol	5.237499	1704
Lansoprazole sodium	5.237319	1705
Aniracetam	5.2372723	1706
estradiol-valerate	5.2371483	1707
tetroquinone	5.2370076	1708
Hydrochlorothiazide	5.236852	1709
cyanopindolol	5.2365484	1710
Ropivacaine hydrochloride monohydrate	5.236512	1711
nitrazepam	5.236451	1712
cefpodoxime	5.2362547	1713
cytarabine	5.236125	1714
phenoxybenzamine	5.2360907	1715
loreclezole	5.2360706	1716
Lonafarnib	5.236067	1717
Lafutidine	5.236065	1718
tenidap	5.2357855	1719
Dyclonine HCl	5.23577	1720
Azaperone	5.23574	1721
trimetazidine	5.235739	1722
SAL-1	5.2356977	1723
Diacerein	5.235689	1724
III606050	5.235565	1725
tyrphostin-AG-126	5.2355194	1726
purmorphamine	5.2354565	1727
Boceprevir	5.2354403	1728
dyphylline	5.235326	1729
oleoylethanolamide	5.235258	1730
Indomethacin	5.2351265	1731
mirtazapine	5.234815	1732

Mesoridazine Besylate	5.234707	1733
Candesartan	5.234602	1734
pilocarpine	5.2344112	1735
Daidzein	5.2342834	1736
Folic acid	5.234216	1737
7,4'-dihydroxyflavone	5.233875	1738
IKK3-inhibitor-IX	5.233799	1739
targinine	5.233735	1740
modafinil	5.233683	1741
tiabendazole	5.233611	1742
cerulenin	5.233348	1743
sitagliptin	5.233306	1744
Carteolol HCl	5.2332335	1745
(6-)卢g-??minocaproic acid	5.233079	1746
piretanide	5.232953	1747
urosiol	5.232897	1748
PHA-665752	5.232875	1749
farnesylpyrophosphate	5.232866	1750
Acetylcysteine	5.2327795	1751
Fenoprofen Calcium	5.232626	1752
cefaclor	5.2325892	1753
Pyridostigmine Bromide	5.2325373	1754
larixinic-acid	5.232464	1755
brefeldin-a	5.232197	1756
N-Carbamyl-L-glutamic acid	5.2321796	1757
lamivudine	5.2319565	1758
Eslicarbazepine acetate	5.231743	1759
loracarbef	5.231656	1760
hydrastinine	5.231594	1761
cardamonin	5.2314906	1762
ponatinib	5.231326	1763
dexfenfluramine	5.230748	1764
Bethanechol chloride	5.230698	1765
salsolidine	5.2306232	1766
pheniramine	5.230599	1767
BX-795	5.2305593	1768
Daunorubicin HCl	5.2304935	1769
Sumatriptan Succinate	5.2303085	1770
methyldopa	5.23024	1771
acetylsalicylsalicylic-acid	5.230201	1772
amuvatinib	5.2301817	1773
tolfenamic-acid	5.2301445	1774
roxithromycin	5.2298408	1775
sulfamerazine	5.2296085	1776
1,2-propylene-glycol	5.229603	1777
olopatadine	5.229577	1778
lisinopril	5.2294617	1779
moxisylyte	5.229411	1780
fillalbin	5.2293386	1781
Fluorouracil (Adrucil)	5.229232	1782
N-arachidonyl-GABA	5.2291875	1783

cefazolin	5.229083	1784
tosyllysyl-chloromethyl-ketone	5.2289276	1785
Tivantinib (ARQ 197)	5.228862	1786
ephedrine	5.228755	1787
isocarboxazid	5.2287426	1788
Embelin	5.228668	1789
zalcitabine	5.2285404	1790
levosulpiride	5.228464	1791
Minocycline HCl	5.2284536	1792
tienilic-acid	5.228403	1793
torin-2	5.2281265	1794
Doxapram HCl	5.2279625	1795
Isoconazole nitrate	5.2274117	1796
westcort	5.2270803	1797
cyclobenzaprine	5.227029	1798
guaiacol	5.2268643	1799
Cabozantinib malate (XL184)	5.2266126	1800
Atropine	5.2264423	1801
cucurbitacin-i	5.226404	1802
7-nitroindazole	5.2261496	1803
Vorinostat (SAHA, MK0683)	5.226146	1804
dexchlorpheniramine	5.2259073	1805
Dexrazoxane	5.225624	1806
Pirfenidone	5.2256155	1807
fenipentol	5.225466	1808
TG101348 (SAR302503)	5.2252784	1809
dichloroacetic-acid	5.225257	1810
phenylbutyrate	5.2251916	1811
fludarabine	5.2251234	1812
Enzastaurin (LY317615)	5.224866	1813
cytosporone-b	5.224864	1814
bemesetron	5.2248306	1815
ABT-199	5.2248173	1816
Tigecycline	5.2248077	1817
Naproxen Sodium	5.22478	1818
coumestrol	5.224517	1819
Bosentan Hydrate	5.2242727	1820
Metoclopramide	5.2242165	1821
dofetilide	5.223831	1822
oxidopamine	5.2235117	1823
oxybenzone	5.2232227	1824
Clofarabine	5.223057	1825
mead-ethanolamide	5.222907	1826
Arsonic acid	5.222826	1827
Amfenac Sodium Monohydrate	5.222823	1828
Evacetrapib (LY2484595)	5.222807	1829
Antipyrine	5.222582	1830
heraclenol	5.222477	1831
GLPG0634	5.222308	1832
dibutyrylcyclic-gmp	5.2222395	1833
Bromhexine HCl	5.2217846	1834

Chlorpheniramine Maleate	5.221569	1835
Mianserin HCl	5.2215543	1836
pidotimod	5.220832	1837
Esomeprazole Sodium	5.2206078	1838
kaempferol	5.220585	1839
Butoconazole nitrate	5.220519	1840
4-(2-amino-ethyl)-benzenesulfonamide	5.2203355	1841
Tropicamide	5.220288	1842
semaxanib	5.220128	1843
milnacipran	5.2199926	1844
doxofylline	5.2197986	1845
diphenhydramine	5.219455	1846
Meloxicam (Mobic)	5.219268	1847
loperamide	5.2190704	1848
Betahistine 2HCl	5.219055	1849
metamizole	5.2189693	1850
Fenticonazole Nitrate	5.218814	1851
tizanidine	5.218563	1852
sulpiride	5.2185545	1853
ketanserin	5.2185316	1854
clopamide	5.2182846	1855
glycopyrrolate	5.2180853	1856
idebenone	5.218038	1857
raltitrexed	5.21774	1858
Acamprosate calcium	5.2175274	1859
Ozagrel	5.2174993	1860
flucytosine	5.2170787	1861
acetylcysteine	5.216997	1862
clindamycin	5.2169437	1863
zaprinast	5.2167463	1864
Pramipexole dihydrochloride	5.2166758	1865
enobosarm	5.216672	1866
Nepafenac	5.216588	1867
Nilotinib monohydrochloride monohydrate	5.216551	1868
alfacalcidol	5.2162733	1869
Metyrapone	5.2161245	1870
ceforanide	5.216048	1871
Rizatriptan Benzoate	5.2160006	1872
phenanthridone	5.2158756	1873
Sulfapyridine	5.2157097	1874
thiazolopyrimidine	5.2157073	1875
melatonin	5.2156157	1876
vidarabine	5.2156076	1877
Famciclovir	5.215583	1878
Torsemide	5.215575	1879
Amorolfine HCl	5.215478	1880
triflusal	5.2152667	1881
ropivacaine	5.215029	1882
Mirtazapine	5.215029	1882
SYK-inhibitor	5.2149925	1884
chlordiazepoxide	5.214754	1885

penciclovir	5.2147155	1886
carbachol	5.2144003	1887
Dovitinib (TKI258) Lactate	5.214394	1888
hesperidin	5.214303	1889
atorvastatin	5.214278	1890
Curcumin	5.214101	1891
aspirin	5.214057	1892
serotonin	5.2139187	1893
givinostat	5.2138453	1894
warfarin	5.2138214	1895
Trametinib DMSO solvate	5.213806	1896
epigallocatechin-gallate-(-)	5.21337	1897
rimonabant	5.2131944	1898
Tiagabine hydrochloride	5.2130136	1899
salvinorin-a	5.212991	1900
Betaine hydrochloride	5.2127466	1901
Histamine Phosphate	5.2125797	1902
bietaserpine	5.212494	1903
paracetamol	5.212403	1904
cinacalcet	5.212143	1905
Carbazochrome sodium sulfonate (AC-17)	5.212044	1906
Prazosin HCl	5.2116613	1907
AGK-2	5.211649	1908
Cilengitide	5.2116456	1909
azacitidine	5.21161	1910
scoulerine	5.2115965	1911
emedastine	5.211347	1912
batimastat	5.2112083	1913
epigallocatechin	5.2111044	1914
graveoline	5.210925	1915
Flucytosine	5.2108417	1916
acetyl-geranyl-cysteine	5.210809	1917
dexbrompheniramine	5.210525	1918
Roxatidine Acetate HCl	5.2105064	1919
methocarbamol	5.2104197	1920
Flurbiprofen	5.209916	1921
Rucaparib (AG-014699,PF-01367338)	5.209902	1922
benidipine	5.209888	1923
doxazosin	5.209865	1924
andarine	5.209842	1925
rolitetracycline	5.20977	1926
alda-1	5.209569	1927
zopiclone	5.209553	1928
triacsin-c	5.2095203	1929
enevalproate	5.2094684	1930
Amoxapine	5.20945	1931
Teriflunomide	5.2093754	1932
mestinon	5.2092934	1933
carbadox	5.2090664	1934
BOX-5	5.20883	1935
fosfosal	5.208743	1936

cefuroxime	5.2086053	1937
pentolinium	5.2085953	1938
levetiracetam	5.20856	1939
INCB-024360	5.2084265	1940
molsidomine	5.2083883	1941
Cefepime Dihydrochloride Monohydrate	5.208194	1942
IKK-2-inhibitor-V	5.2080264	1943
YM155	5.2079983	1944
Sulfadimethoxine	5.2079067	1945
Raltegravir (MK-0518)	5.207802	1946
nimetazepam	5.2077827	1947
glimepiride	5.207756	1948
carbaryl	5.207674	1949
chlorpromazine	5.2075214	1950
Roflumilast	5.207378	1951
Cefprozil	5.207318	1952
Cyclosporin A	5.2071304	1953
Linezolid	5.20701	1954
SJ-172550	5.2068763	1955
IAA-94	5.206841	1956
picotamide	5.2067757	1957
Brompheniramine hydrogen maleate	5.2066565	1958
Dantrolene, sodium salt	5.2066364	1959
AICA-ribonucleotide	5.206336	1960
moroxydine	5.2063313	1961
dimercaptosuccinic-acid	5.2060404	1962
Cefazolin (sodium salt)	5.205963	1963
latanoprost	5.2058535	1964
Regorafenib	5.2056503	1965
6-nitronorepinephrine	5.205634	1966
dienestrol	5.205365	1967
amikacin	5.205163	1968
Fludarabine Phosphate (Fludara)	5.2050714	1969
mirin	5.2050123	1970
Raloxifene HCl	5.205008	1971
Losartan Potassium (DuP 753)	5.204997	1972
levobunolol	5.2049904	1973
Erythromycin Ethylsuccinate	5.204882	1974
Erdosteine	5.2046947	1975
amlodipine	5.204532	1976
Fluoro-SAHA	5.204322	1977
Rocilinostat (ACY-1215)	5.204217	1978
Fludarabine	5.2041883	1979
levomepromazine	5.2036705	1980
trichostatin-a	5.2036376	1981
tyramine	5.2036076	1982
thiamphenicol	5.2034574	1983
DAPT (GSI-IX)	5.2033587	1984
trihexyphenidyl	5.203286	1985
APO-866	5.2032566	1986
Doxifluridine	5.203146	1987

Tolperisone HCl	5.203092	1988
Zileuton	5.203067	1989
Ceftiofur HCl	5.2030125	1990
esatenolol	5.202987	1991
benserazide	5.2027493	1992
tyrphostin-AG-99	5.202648	1993
nifuroxazide	5.2026467	1994
Loratadine	5.2024593	1995
Cefotaxime (sodium salt)	5.2024555	1996
terconazole	5.2023983	1997
Nelarabine	5.202298	1998
monastrol	5.202228	1999
papaverine	5.2020664	2000
Taurine	5.2020054	2001
Amprenavir (agenerase)	5.201825	2002
Dexmedetomidine HCl	5.2017975	2003
sulfasalazine	5.201729	2004
Nitrendipine	5.2017283	2005
Niclosamide	5.201586	2006
Idarubicin HCl	5.201339	2007
geranyl-geranyl-pyrophosphate	5.2012553	2008
rescinnamine	5.200801	2009
granisetron	5.2005615	2010
hexamethyleneamiloride	5.200494	2011
TCB2	5.2004757	2012
Broxyquinoline	5.200462	2013
n-methylphenothiazine	5.2004538	2014
entecavir	5.2004294	2015
Betamethasone Valerate	5.2003717	2016
diphenylpyraline	5.20026	2017
Pyrazinamide	5.200181	2018
desoximetasone	5.200064	2019
Zalcitabine	5.199971	2020
phenprobamate	5.1999474	2021
ABT-888 (Veliparib)	5.199921	2022
amlexanox	5.199911	2023
indole-3-carbinol	5.199818	2024
alfadolone	5.199813	2025
Ruxolitinib phosphate	5.199705	2026
remoxipride	5.199673	2027
farnesylcysteine-methyl-ester	5.1993527	2028
Dabigatran etexilate mesylate	5.1993494	2029
Sorafenib	5.199336	2030
phenformin	5.1989098	2031
JAK3-inhibitor-I	5.198884	2032
5-aminolevulinic-acid	5.1987686	2033
Brexpiprazole	5.198704	2034
Dimethyl Fumarate	5.1986246	2035
merbromin	5.198312	2036
forskolin	5.1982403	2037
roxarsone	5.1982083	2038

Methicillin (sodium salt)	5.1981597	2039
Serotonin HCl	5.198094	2040
Difloxacin HCl	5.1980286	2041
adipiodone	5.1980133	2042
Cilnidipine	5.197838	2043
Haloperidol	5.1975913	2044
Entecavir Hydrate	5.1975217	2045
fenspiride	5.197484	2046
ritonavir	5.197298	2047
furaltadone	5.197144	2048
skatole	5.196956	2049
theophylline	5.1968527	2050
succinylsulfathiazole	5.196819	2051
equol	5.1966934	2052
syrosingopine	5.196526	2053
Mexiletine HCl	5.1964064	2054
plinabulin	5.1962633	2055
Ritonavir	5.196124	2056
Oxiconazole (nitrate)	5.1959476	2057
Diperodon HCl	5.1958666	2058
clarithromycin	5.1958385	2059
acefylline	5.1957784	2060
Apatinib	5.1957035	2061
Trazodone HCl	5.195606	2062
adenosine-phosphate	5.1955976	2063
tolnaftate	5.1949973	2064
Mepenzolate Bromide	5.1948385	2065
isoeugenol	5.1947775	2066
IKK-inhibitor-X	5.1947727	2067
Carprofen	5.1946917	2068
lypressin	5.1946497	2069
PD 0332991 (Palbociclib) HCl	5.194579	2070
Pemirolast potassium	5.194252	2071
Gossypetin	5.1942015	2072
isonicotinamide	5.194079	2073
Salicylanilide	5.194028	2074
selegiline	5.1937904	2075
Droperidol	5.1937103	2076
tranexamic-acid	5.1935253	2077
Levetiracetam	5.193512	2078
Pentamidine isethionate	5.193432	2079
khellin	5.193393	2080
5-iodotubercidin	5.193352	2081
Halobetasol Propionate	5.1932454	2082
15-delta-prostaglandin-j2	5.1931105	2083
atomoxetine	5.1928167	2084
goserelin	5.1927614	2085
Fluconazole hydrate	5.1926556	2086
trametinib	5.19236	2087
Bromfenac Sodium	5.1921797	2088
imidazolidinyl-urea	5.1920924	2089

Fosaprepitant dimeglumine salt	5.191929	2090
bromperidol	5.1918745	2091
Clopidogrel	5.1917624	2092
florfenicol	5.191532	2093
Ondansetron HCl	5.191449	2094
Lenalidomide (CC-5013)	5.1914206	2095
norepinephrine	5.191228	2096
ganciclovir	5.191206	2097
Clevidipine Butyrate	5.191101	2098
nafadotride	5.1909823	2099
Carbamazepine	5.190925	2100
methyltyramine	5.190776	2101
Quinestrol	5.1907673	2102
nifekalant	5.1907034	2103
Cyromazine	5.1906714	2104
etoposide	5.1905465	2105
Vortioxetine (Lu AA21004) HBr	5.190428	2106
clopidogrel	5.190327	2107
Oxaliplatin	5.189962	2108
Ellagic acid	5.189847	2109
Nimodipine	5.189741	2110
mycophenolic-acid	5.189724	2111
chlorhexidine	5.1897197	2112
sinensetin	5.1896777	2113
Ibuprofen Lysine	5.1894503	2114
Pramipexole 2HCl Monohydrate	5.189188	2115
Oxybutynin chloride	5.1888924	2116
tioguanine	5.188785	2117
IKK-2-inhibitor	5.1887684	2118
tacedinaline	5.1886463	2119
nicotinamide	5.1885834	2120
levofloxacin	5.1885014	2121
TER-14687	5.188417	2122
Nicotinic Acid	5.188223	2123
hexamethylenebisacetamide	5.1880836	2124
Dirithromycin	5.1880627	2125
malonoben	5.1879177	2126
homoveratrylamine	5.1877074	2127
ACP-196	5.187468	2128
Flutamide	5.1873255	2129
mephenesin	5.187315	2130
parachlorophenol	5.1871977	2131
BX-912	5.1871195	2132
Carbadox	5.186477	2133
marbofloxacin	5.1863265	2134
Fenspiride HCl	5.1863184	2135
Rivastigmine Tartrate	5.1862693	2136
Etomidate hydrochloride	5.1858425	2137
benzofuran	5.185482	2138
Belinostat (PXD101)	5.185458	2139
ITE	5.1853867	2140

PATI-1-2-3	5.1853533	2141
purpurogallin	5.185342	2142
pentamidine	5.185321	2143
farnesylthiotriazole	5.1850758	2144
atropine	5.1850357	2145
bupivacaine	5.184774	2146
oxolamine	5.1843843	2147
eudesmic-acid	5.184334	2148
MAPP-D-erythro	5.1843157	2149
miglitol	5.18427	2150
mecillinam	5.1840296	2151
alvespimycin	5.1839924	2152
prunetin	5.183879	2153
myricetin	5.1837306	2154
Valganciclovir HCl	5.1836743	2155
fadrozole	5.183644	2156
homatropine	5.1835637	2157
tracazolate	5.183464	2158
AG-221 (Enasidenib)	5.1833005	2159
Ethambutol HCl	5.183181	2160
Tacrine hydrochloride	5.1831617	2161
felodipine	5.1826553	2162
Safinamide Mesylate	5.182626	2163
Olaparib (AZD2281, Ku-0059436)	5.1824055	2164
6-aminochrysene	5.1822042	2165
Inosine	5.182123	2166
promethazine	5.1820793	2167
Proxyphylline	5.1819334	2168
chlorothiazide	5.181884	2169
Chlorogenic acid	5.1817017	2170
Phenacetin	5.1813526	2171
quercetin	5.18132	2172
raclopride	5.1812897	2173
Homatropine Bromide	5.1812844	2174
VX-680 (MK-0457,Tozasertib)	5.1812363	2175
Etomidate	5.181075	2176
linezolid	5.181006	2177
plumbagin	5.1808662	2178
Zinc Pyrithione	5.1807604	2179
Sulfisoxazole Acetyl	5.1807356	2180
colchicine	5.180484	2181
Mesna	5.1803913	2182
Tranexamic Acid	5.180087	2183
sorafenib	5.179902	2184
Dapagliflozin	5.179661	2185
azasetron	5.179331	2186
bortezomib	5.179287	2187
Pitolisant hydrochloride	5.1792846	2188
sulfacetamide	5.1791496	2189
talniflumate	5.17887	2190
Cladribine	5.178622	2191

5-Methoxypsoralen	5.17859	2192
Alogliptin (SYR-322)	5.178491	2193
L-erythro-MAPP	5.1784906	2194
thiocolchicoside	5.178273	2195
Laquinimod (ABR-215062)	5.178266	2196
Caffeic acid	5.1782284	2197
5-methoxytryptamine	5.1780834	2198
rotenone	5.177776	2199
anisomycin	5.1775403	2200
Acyclovir	5.177182	2201
z-prolyl-prolinal	5.177154	2202
cephalexin	5.1770983	2203
Gemcitabine	5.177063	2204
ruxolitinib	5.176985	2205
esculin	5.1769166	2206
nimodipine	5.1768007	2207
hydrastine-(1r,-9s)	5.1767936	2208
Cisapride	5.176785	2209
Fluvoxamine maleate	5.17671	2210
Torcetrapib	5.176707	2211
Meticrane	5.176653	2212
Chloroxine	5.1760178	2213
foretinib	5.1758647	2214
xylometazoline	5.1756916	2215
tubaic-acid	5.1756315	2216
Carfilzomib (PR-171)	5.1753683	2217
morphothebaine	5.1753397	2218
oxyphenbutazone	5.1751537	2219
dopamine	5.1750774	2220
Trifluoperazine 2HCl	5.1749387	2221
Cinchophen	5.174789	2222
hemado	5.1747136	2223
aminohippuric-acid	5.174635	2224
tiotidine	5.1744123	2225
Molidustat (BAY85-3934)	5.1743994	2226
roquinimex	5.1743293	2227
Cinnamic acid	5.1742907	2228
alosetron	5.174286	2229
Radotinib(IY-5511)	5.1741796	2230
Mitiglinide Calcium	5.1739454	2231
FT-207 (NSC 148958)	5.1739244	2232
nilutamide	5.173676	2233
Canagliflozin	5.173617	2234
saclofen	5.173583	2235
Rabeprazole	5.1734715	2236
Thioridazine HCl	5.173278	2237
Dimesna	5.173243	2238
Eltrombopag	5.173188	2239
Azithromycin	5.1729074	2240
Prasugrel	5.172868	2241
Pemetrexed	5.172806	2242

nialamide	5.172803	2243
idazoxan	5.172777	2244
acetyl-farnesyl-cysteine	5.1725817	2245
Didanosine	5.172537	2246
salsolinol	5.172496	2247
9-methyl-5H-6-thia-4,5-diaza-chrysene-6,6-dioxide	5.1724515	2248
noscapine	5.1724386	2249
Azelastine HCl	5.172408	2250
APEC	5.1723557	2251
2-(4-methoxybenzylthio)-6-methylpyrimidin-4-ol	5.1723547	2252
trimethobenzamide	5.1722474	2253
7,8-dihydro-L-biopterin	5.1720257	2254
lymecycline	5.1719985	2255
Sarpogrelate hydrochloride	5.1718674	2256
lomerizine	5.1718626	2257
Tenoxicam	5.1717415	2258
morin	5.1717377	2259
Oxybutynin	5.1716547	2260
Conivaptan HCl	5.171614	2261
zidovudine	5.171602	2262
Citalopram hydrobromide	5.1713934	2263
deoxyrhapontin	5.171357	2264
triacetylresveratrol	5.171299	2265
droxinostat	5.171216	2266
rilmenidine	5.17113	2267
Prasugrel hydrochloride	5.171041	2268
sphingosine	5.170933	2269
pyrimethamine	5.170437	2270
proglumide	5.1703873	2271
arbutin	5.1703825	2272
NCI-16221	5.1702356	2273
trimidox	5.170207	2274
Tamoxifen	5.169775	2275
Prostaglandin F2α	5.169434	2276
tyrphostin-AG-835	5.169429	2277
Dronedarone	5.1693897	2278
Phloridzin	5.169385	2279
levocabastine	5.1692085	2280
Carvedilol	5.1690354	2281
tyrphostin-B44	5.168949	2282
L-Carnitine inner salt	5.168852	2283
Sumatriptan	5.1688156	2284
Pramiracetam	5.168748	2285
Altretamine	5.1685014	2286
oxcarbazepine	5.1684294	2287
lawsone	5.1683574	2288
butenafine	5.168338	2289
methyl-everninic-acid	5.168238	2290
norcitalopram	5.1680775	2291
CP-945598 HCl	5.167877	2292
Acemetacin	5.16774	2293

phorbol-12-myristate-13-acetate	5.1676702	2294
Betaxolol HCl	5.1673512	2295
sulfadoxine	5.166951	2296
piceatannol	5.1667743	2297
crizotinib	5.166772	2298
rotenonic-acid	5.1667347	2299
nerol	5.166729	2300
Idoxuridine	5.166416	2301
PCI-24781 (CRA-024781)	5.1663165	2302
enzalutamide	5.1662393	2303
Pimobendan	5.1661835	2304
EHPG-piperazine	5.1661615	2305
Nabumetone	5.16611	2306
Bisacodyl	5.1660814	2307
NCS-382	5.1660347	2308
haloprogin	5.165854	2309
Ruxolitinib (INCB018424)	5.165811	2310
Nilotinib(AMN-107)	5.165739	2311
Chlorambucil	5.1657104	2312
Thiamine HCl (Vitamin B1)	5.165597	2313
Pracinostat (SB939)	5.165593	2314
Repaglinide	5.165331	2315
spermidine	5.1652517	2316
vicriviroc	5.1647387	2317
Dibenzothiophene	5.164701	2318
Deoxyarbutin	5.1645803	2319
balsalazide	5.1645384	2320
iobenguane	5.164324	2321
perospirone	5.164316	2322
Betamipron	5.1643114	2323
SJB-shh-31	5.164065	2324
tandutinib	5.164048	2325
Saquinavir mesylate	5.16393	2326
Cabozantinib (XL184, BMS-907351)	5.163924	2327
Troxerutin	5.1637783	2328
Celecoxib	5.163479	2329
T 705	5.1634545	2330
benzo(e)pyrene	5.1632605	2331
Cytarabine hydrochloride	5.163225	2332
MNITMT	5.162711	2333
Rocuronium Bromide	5.1626534	2334
ISOX	5.1626425	2335
pinocembrin	5.162449	2336
Granisetron HCl	5.1624084	2337
IM-12	5.1621494	2338
Trelagliptin	5.161909	2339
Edoxaban tosylate monohydrate	5.16188	2340
norfloxacin	5.161664	2341
dinorcitalopram-oxalate	5.161577	2342
Mequinol	5.1614923	2343
latamoxef	5.161462	2344

Sildenafil	5.161236	2345
lincomycin	5.1611958	2346
metaxalone	5.160963	2347
pemetrexed	5.1604757	2348
haematoxylin-pentaacetate	5.1604605	2349
Ganetespib (STA-9090)	5.160438	2350
octopamine	5.160411	2351
L-Thyroxine	5.1603985	2352
herniarin	5.160381	2353
Diclazuril	5.1603727	2354
ACDPP	5.160349	2355
cefotetan	5.160121	2356
Pramoxine HCl	5.160116	2357
Mycophenolic acid	5.1600685	2358
Pyridoxine HCl	5.1599455	2359
siguazodan	5.159792	2360
5-Azacytidine	5.1596107	2361
IB-MECA	5.1593485	2362
PKCbeta-inhibitor	5.159346	2363
Tolbutamide	5.159286	2364
nor-2-chlorpromazine	5.1590495	2365
L-ascorbyl-6-palmitate	5.1590357	2366
Pralatrexate	5.158992	2367
Chloroquine diphosphate	5.158968	2368
Valaciclovir HCl	5.158784	2369
ethaverine	5.1586823	2370
baclofen	5.158532	2371
bosentan	5.1584673	2372
Miglitol	5.158411	2373
diperodon	5.158164	2374
amfepramone	5.1581135	2375
citiolone	5.158059	2376
1-phenylbiguanide	5.1579237	2377
Atovaquone	5.157524	2378
Pitavastatin Calcium	5.157481	2379
nifenazone	5.15734	2380
Baricitinib (LY3009104, INCB028050)	5.1571207	2381
cobalt(II)-chloride	5.1569033	2382
Mepivacaine HCl	5.156827	2383
Azithromycin Dihydrate	5.156823	2384
Azilsartan	5.1568108	2385
nortriptyline	5.1566825	2386
Brimonidine Tartrate	5.1565814	2387
Cobicistat (GS-9350)	5.1565714	2388
Nimesulide	5.156353	2389
acenocoumarol	5.1562395	2390
Luliconazole	5.1560936	2391
lamotrigine	5.1558347	2392
17-hydroxyprogesterone-caproate	5.1556454	2393
Colchicine	5.155534	2394
Adenine HCl	5.1555157	2395

Ciclopirox	5.155513	2396
Candesartan Cilexetil	5.155512	2397
lenalidomide	5.155301	2398
Triciribine	5.1552315	2399
acetohexamide	5.155051	2400
Pitavastatin	5.1549025	2401
Alibendol	5.154742	2402
spaglumic-acid	5.154717	2403
STAT3-inhibitor-VI	5.1546593	2404
lorazepam	5.1545997	2405
Posaconazole	5.1543684	2406
pentachlorophenol	5.1542854	2407
DASB	5.154254	2408
lopinavir	5.154232	2409
etamsylate	5.1541796	2410
dapsone	5.154172	2411
dinoprost	5.154098	2412
phenoxazine	5.1538916	2413
lonidamine	5.153754	2414
Metaxalone	5.1532135	2415
Cloxacillin Sodium	5.1531677	2416
Azaguanine-8	5.15313	2417
kynuramine	5.153122	2418
valaciclovir	5.1529837	2419
dicloxacillin	5.15292	2420
Tranilast	5.15283	2421
16,16-dimethylprostaglandin-e2	5.1527195	2422
Xylometazoline HCl	5.152606	2423
1-benzylimidazole	5.1525974	2424
ERK-inhibitor-11E	5.152482	2425
N-(3-acetamidophenyl)-3-chlorobenzamide	5.152318	2426
2',5'-dideoxyadenosine	5.1522484	2427
clodronic-acid	5.152004	2428
neratinib	5.1519365	2429
gentamicin	5.151634	2430
Amiloride HCl dihydrate	5.1512246	2431
ganglioside	5.1511946	2432
Tinidazole	5.1511517	2433
Sodium butyrate	5.151	2434
methimazole	5.1509333	2435
trapidil	5.150906	2436
etanidazole	5.150895	2437
10H-phenothiazin-10-yl)(p-tolyl)methanone	5.1508336	2438
midazolam	5.150775	2439
tofacitinib	5.150692	2440
oxacillin	5.1506042	2441
cefotaxime	5.1505966	2442
vanillyl-glycol	5.150497	2443
salermide	5.1503067	2444
fexaramine	5.150009	2445
Neostigmine Bromide	5.1496983	2446

Domperidone	5.1496944	2447
diphencyprone	5.149557	2448
(R)-Crizotinib	5.149513	2449
tetramethyl-haematoxylone	5.1492834	2450
Dacomitinib (PF299804, PF299)	5.1492424	2451
isoniazid	5.1490946	2452
Nelfinavir Mesylate	5.148857	2453
nicotinyl-alcohol-tartrate	5.1487093	2454
Sucralose	5.148527	2455
Olprinone Hydrochloride	5.1483307	2456
yohimbine	5.1482124	2457
narciclasine	5.1480637	2458
cotinine	5.14771	2459
IC-261	5.1472874	2460
oxymetazoline	5.147089	2461
chicago-sky-blue-6b	5.1469364	2462
deferiprone	5.1468744	2463
tivozanib	5.146862	2464
Epalrestat	5.1468143	2465
zimelidine	5.1467667	2466
Diosmetin	5.1466846	2467
Pargyline (hydrochloride)	5.1462955	2468
Clemizole hydrochloride	5.1462	2469
triciribine	5.14604	2470
Oltipraz	5.1458564	2471
pevonedistat	5.145807	2472
Doxazosin Mesylate	5.1457925	2473
cetraxate	5.145694	2474
Ramelteon	5.1456795	2475
Doxorubicin	5.1454287	2476
sulmazole	5.145071	2477
secnidazole	5.1450186	2478
Levosulpiride	5.14499	2479
Chlorotrianisene	5.144965	2480
iohexol	5.1449385	2481
nitazoxanide	5.1448917	2482
dinoprostone	5.144655	2483
gossypin	5.1445765	2484
fatostatin	5.1444626	2485
timolol	5.144437	2486
Sodium ascorbate	5.1444287	2487
oxamflatin	5.1444063	2488
Mubritinib (TAK 165)	5.1443763	2489
Pramipexole	5.14414	2490
neostigmine	5.1439776	2491
JAK3-inhibitor-VI	5.143818	2492
palbociclib	5.1437936	2493
Triclosan	5.143569	2494
kenpaullone	5.1434655	2495
epirubicin	5.1433897	2496
phloretin	5.1431093	2497

Sotalol	5.1430845	2498
Acipimox	5.143046	2499
belinostat	5.1430187	2500
Rivaroxaban	5.142822	2501
xanthohumol	5.142808	2502
isoliquiritigenin	5.14247	2503
Avanafil	5.14204	2504
Cefpodoxime Proxetil	5.142005	2505
Valdecoxib	5.1419697	2506
Nicorandil	5.1419096	2507
isonicotinohydroxamic-acid	5.1417937	2508
risperidone	5.1414833	2509
KPT-330	5.141432	2510
pargyline	5.141348	2511
COT-10b	5.141075	2512
Erlotinib Hydrochloride	5.1410713	2513
gallic-acid	5.141033	2514
Kinetin	5.140984	2515
Methylthiouracil	5.140876	2516
levothyroxine	5.1408424	2517
Pexidartinib (PLX3397)	5.1405296	2518
xanthoxyline	5.1404147	2519
Timolol Maleate	5.1402683	2520
Sulfacetamide Sodium	5.140255	2521
minoxidil	5.1402345	2522
Apremilast (CC-10004)	5.14017	2523
Prochlorperazine	5.1400924	2524
closantel	5.13991	2525
oxindole-I	5.139909	2526
Iohexol	5.1398845	2527
EX 527 (SEN0014196)	5.139858	2528
sulfamethoxypyridazine	5.1398134	2529
ascorbic-acid	5.139681	2530
nadide	5.1394844	2531
R-96544	5.139344	2532
2-aminobenzenesulfonamide	5.1392517	2533
dibutyryl-cAMP-Na	5.1387453	2534
Dorzolamide HCl	5.138638	2535
Nithiamide	5.138534	2536
sulfadiazine	5.138464	2537
Panobinostat (LBH589)	5.138156	2538
pirenzepine	5.137944	2539
Ozanimod (RPC1063)	5.137925	2540
4,5,6,7-tetrabromobenzotriazole	5.1376295	2541
IBC-293	5.1375875	2542
TWS-119	5.137418	2543
iopromide	5.137272	2544
Atorvastatin Calcium	5.1372485	2545
Leuprolide Acetate	5.1372213	2546
Eltrombopag Olamine	5.1371913	2547
methazolamide	5.137088	2548

oxiconazole	5.137018	2549
AN-2728	5.136981	2550
iloprost	5.136895	2551
fomepizole	5.1366844	2552
azelastine	5.136546	2553
mevastatin	5.1363964	2554
Pelitinib (EKB-569)	5.1362267	2555
suxibuzone	5.1361923	2556
Cyclamic acid	5.135792	2557
emodin	5.1356196	2558
Asenapine	5.1355886	2559
Flupirtine maleate	5.135491	2560
PCI-32765 (Ibrutinib)	5.135437	2561
Zolmitriptan	5.135124	2562
LDE225 (NVP-LDE225,Erismodegib)	5.1351223	2563
KB188-III-56	5.135066	2564
exifone	5.1349964	2565
sulfachlorpyridazine	5.1349754	2566
chlorprothixene	5.1349115	2567
tipifarnib-P2	5.1349077	2568
Orlistat	5.1348505	2569
Hydroxyfasudil hydrochloride	5.1347065	2570
ebselen	5.13427	2571
clorgiline	5.1340466	2572
fenoprofen	5.1339436	2573
5-nonyloxytryptamine	5.1338444	2574
Rigosertib (ON-01910,Estybon)	5.133837	2575
calpeptin	5.13381	2576
Pioglitazone	5.1337805	2577
Chlorpropamide	5.13373	2578
Roxithromycin	5.133712	2579
VEGF-receptor-2-kinase-inhibitor-IV	5.133665	2580
Ethamsylate	5.133478	2581
ormetoprim	5.1334515	2582
Ambrisentan	5.1333685	2583
esomeprazole	5.1330833	2584
furosemide	5.1330404	2585
hexachlorophene	5.132946	2586
bucladesine	5.13291	2587
PSI-7977	5.1326365	2588
chrysenequinone	5.132609	2589
tyrphostin-A9	5.132536	2590
anethole	5.132408	2591
BAY-K8644	5.1322203	2592
VER-155008	5.132181	2593
tubastatin-a	5.13211	2594
orteronel	5.132099	2595
BCI-hydrochloride	5.1318607	2596
evodiamine	5.1318483	2597
streptozotocin	5.1318016	2598
Amisulpride	5.13175	2599

nefazodone	5.131701	2600
psoromic-acid	5.1316957	2601
ibudilast	5.1316776	2602
Menadione	5.1316066	2603
AT13387	5.1316032	2604
allantoxanamide	5.131481	2605
Heparin sodium	5.1313305	2606
Lomustine	5.1312733	2607
cefadroxil	5.1305733	2608
Doxorubicin (Adriamycin) HCl	5.1301374	2609
TPCA-1	5.129694	2610
Enoxacin (Penetrex)	5.129302	2611
genistein	5.129247	2612
Arbidol HCl	5.1292057	2613
Malotilate	5.1291046	2614
ipidacrine	5.1289186	2615
6-nitrodopamine	5.1288853	2616
17-beta-estradiol	5.1288624	2617
rhizocarpic-acid	5.1287527	2618
proxyfan	5.128702	2619
hexylcaine	5.128466	2620
elesclomol	5.1283216	2621
REV-5901	5.1282587	2622
leoidin	5.128108	2623
ronidazole	5.1279826	2624
Losmapimod	5.127742	2625
tosufloxacin	5.1277122	2626
Glimepiride	5.127694	2627
Agomelatine	5.12768	2628
Prucalopride	5.1276145	2629
HC-toxin	5.1274834	2630
Pazopanib (GW-786034)	5.127139	2631
mocetinostat	5.1271377	2632
Eletriptan HBr	5.1270943	2633
acetyl-geranygeranyl-cysteine	5.127074	2634
Imatinib (STI571)	5.127028	2635
zeranol	5.12691	2636
temozolomide	5.126811	2637
neurodazine	5.1265116	2638
Rosiglitazone	5.125923	2639
foxy-5	5.125511	2640
Bendamustine HCl	5.125448	2641
Reboxetine mesylate	5.124953	2642
Tamibarotene	5.1249123	2643
azauridine	5.1248455	2644
ciproxifan	5.1248074	2645
Detomidine HCl	5.1247716	2646
alprazolam	5.124692	2647
sulfamonomethoxine	5.1245155	2648
Ornidazole	5.12419	2649
skimmianine	5.1238565	2650

methoxypyruvic-acid	5.1237903	2651
phenelzine	5.1237373	2652
Paroxetine HCl	5.1236553	2653
STF-62247	5.1235933	2654
Cefoselis Sulfate	5.1234827	2655
carbetocin	5.1234694	2656
miconazole	5.1230474	2657
nifurtimox	5.122997	2658
aflatoxin-b1	5.122978	2659
Lenvatinib (E7080)	5.1226997	2660
Flumazenil	5.1226826	2661
edrophonium	5.1226034	2662
Coumarin	5.122595	2663
Vatalanib (PTK787) 2HCl	5.1225214	2664
palmitoylethanolamide	5.1224813	2665
suramin	5.1224523	2666
Neratinib (HKI-272)	5.122341	2667
Tofacitinib (CP-690550,Tasocitinib)	5.1222496	2668
naftifine	5.1222186	2669
Pranlukast	5.1219635	2670
zibotentan	5.121805	2671
tetrahydrobiopterin	5.1217813	2672
3-amino-benzamide	5.1209154	2673
BAY-K-8644-(S)-(-)	5.1209135	2674
Benzbromarone	5.1208553	2675
tacrine	5.120681	2676
DL-Carnitine HCl	5.1205764	2677
Rasagiline Mesylate	5.120432	2678
Dexmedetomidine	5.1200686	2679
sulindac	5.1199703	2680
Rolipram	5.119261	2681
Bifonazole	5.1192293	2682
aceclofenac	5.119211	2683
honokiol	5.119204	2684
lysylphenylalanyl-tyrosine	5.1190567	2685
SRC-kinase-inhibitor-II	5.1189895	2686
clinafloxacin	5.1189756	2687
flucloxacillin	5.118931	2688
masitinib	5.118905	2689
TBEP	5.1188955	2690
methiazole	5.1188374	2691
mefloquine	5.118819	2692
farnesol	5.1187906	2693
eprosartan	5.118631	2694
Dronedarone HCl	5.1183314	2695
mebendazole	5.1181335	2696
tenofovir	5.1180515	2697
acamprosate	5.11796	2698
Chlorocresol	5.1177597	2699
Ciclopirox ethanolamine	5.117655	2700
taxifolin	5.117629	2701

orantinib	5.117488	2702
bisphenol-a	5.1172647	2703
tetramethylsilane	5.117035	2704
Milrinone	5.116708	2705
Iniparib (BSI-201)	5.116519	2706
Ketoprofen	5.1163707	2707
folic-acid	5.1163645	2708
erbstatin-analog	5.116294	2709
fraxidin	5.116206	2710
tubacin	5.116182	2711
Fasudil (HA-1077) HCl	5.1161723	2712
CX-4945 (Silmitasertib)	5.1161704	2713
azathioprine	5.1160026	2714
GDC-0941	5.1158895	2715
IKK-16	5.1157665	2716
Topiroxostat	5.1152706	2717
TCDD-dioxin	5.1150603	2718
caffeic-acid	5.1149273	2719
phenazopyridine	5.1147685	2720
todralazine	5.114731	2721
gavestinel	5.114667	2722
Thiamphenicol	5.114652	2723
Amidopyrine	5.1144614	2724
ranitidine	5.114373	2725
EMD-1214063	5.114325	2726
motesanib	5.1140947	2727
tipifarnib	5.1137958	2728
irsogladine	5.113786	2729
Capsaicin	5.113716	2730
vemurafenib	5.1133766	2731
Rufinamide	5.113197	2732
sulfabenzamide	5.1131806	2733
Rofecoxib	5.1131606	2734
olomoucine	5.113106	2735
cefixime	5.112999	2736
Clarithromycin	5.1129293	2737
Chlormezanone	5.11281	2738
Salicylic acid	5.112758	2739
tenovin-1	5.112747	2740
Itraconazole	5.1126795	2741
piromidic-acid	5.112566	2742
bicuculline	5.112419	2743
capecitabine	5.112362	2744
Poziotinib	5.1121616	2745
astemizole	5.1120405	2746
Fenoldopam	5.1120205	2747
candesartan	5.112004	2748
Puromycin dihydrochloride	5.1117926	2749
aminophylline	5.1115456	2750
Salmeterol xinafoate	5.111515	2751
rolipram	5.1114016	2752

ICI-89406	5.1114	2753
demecarium	5.1111984	2754
fumonisin-b1	5.1111755	2755
mianserin	5.1110287	2756
nor-1-chlorpromazine	5.110901	2757
Azacyclonol	5.110845	2758
valproxam	5.1107287	2759
IWP-2	5.110667	2760
zolmitriptan	5.1102486	2761
Dexlansoprazole	5.1102324	2762
aminoresveratrol	5.110198	2763
Sulfaphenazole	5.110098	2764
Levomefolate calcium	5.1100283	2765
BIIB-021	5.109885	2766
telenzepine	5.1098375	2767
Monobenzone	5.109739	2768
GDC-0449 (Vismodegib)	5.1093807	2769
bufexamac	5.109368	2770
GM 6001	5.1091423	2771
clemizole	5.109103	2772
Pyridoxine	5.1089334	2773
buparlisib	5.108852	2774
bosutinib	5.1087933	2775
2-aminotetralin	5.1087847	2776
4-hydroxy-2-nonenal	5.1080136	2777
a-disintegrin-and-metalloproteinase	5.1080074	2778
CYT-997	5.1075644	2779
Azathioprine	5.1075306	2780
LDK378	5.107442	2781
brimonidine	5.1073756	2782
ezetimibe	5.107295	2783
pipemidic-acid	5.107063	2784
Paliperidone	5.1067533	2785
NECA	5.1067038	2786
sulfaguanidine	5.1067004	2787
nafcillin	5.1065865	2788
JAK3-inhibitor-V	5.106536	2789
2-(biphenyl-4-ylsulfonamido)pentanedioic-acid	5.1064696	2790
prothionamide	5.1060185	2791
Diclofenac Diethylamine	5.1058717	2792
AN-2690	5.1058083	2793
danusertib	5.10577	2794
ER-27319	5.1057553	2795
MLN8237 (Alisertib)	5.105628	2796
temefos	5.1054974	2797
Carbimazole	5.1054964	2798
Protionamide	5.105464	2799
1,2,3,4-tetrahydroisoquinoline	5.1053295	2800
quizartinib	5.1051397	2801
Mercaptopurine (6-MP)	5.105092	2802
parecoxib	5.1050577	2803

Zafirlukast	5.104989	2804
triphenyl-tin	5.1048517	2805
amifostine	5.1045284	2806
dovitinib	5.104494	2807
vatalanib	5.104392	2808
Atazanavir sulfate (BMS-232632-05)	5.1043463	2809
desmethyl-DASB	5.1040387	2810
ethionamide	5.1040215	2811
thiamine	5.1038065	2812
lobeline	5.103795	2813
MGCD-265	5.1037507	2814
Lapatinib	5.103695	2815
bromfenac	5.1036415	2816
pitavastatin	5.103618	2817
tert-butylhydroquinone	5.1034117	2818
Oxaprozin	5.103249	2819
BAF312 (Siponimod)	5.103061	2820
dihydrexidine	5.102946	2821
Sasapyrine	5.102373	2822
Sulindac	5.102049	2823
Econazole nitrate	5.101935	2824
2-aminopurine	5.101391	2825
LM-1685	5.10091	2826
Dabrafenib (GSK2118436)	5.1006413	2827
geranylgeraniol	5.1006374	2828
didanosine	5.1006203	2829
Diazoxide	5.100596	2830
pramocaine	5.100445	2831
mercaptopurine	5.1004333	2832
TSU-68 (SU6668,Orantinib)	5.100334	2833
sertaconazole	5.1001883	2834
phenindione	5.100047	2835
itopride	5.1000338	2836
LDN-193189	5.099985	2837
Resveratrol	5.0999274	2838
fusaric-acid	5.099656	2839
pyrazolanthrone	5.099514	2840
dihydrocapsaicin	5.099252	2841
Balsalazide	5.0991583	2842
SK-383933	5.0990505	2843
CUDC-101	5.099043	2844
betazole	5.0988398	2845
FH-535	5.098787	2846
propafenone	5.098731	2847
Ethionamide	5.098624	2848
MEK162 (ARRY-162, ARRY-438162)	5.0985994	2849
TC-2559	5.0985017	2850
Escitalopram Oxalate	5.0983467	2851
Thioguanine	5.098276	2852
glafenine	5.098078	2853
Tedizolid	5.0977955	2854

apafant	5.0977116	2855
PF-04217903	5.097611	2856
prostaglandin-e1	5.097557	2857
Benzoic Acid	5.0972843	2858
calmidazolium	5.0971622	2859
Nafamostat Mesylate(FUT-175)	5.0965805	2860
Tioxolone	5.096154	2861
pazopanib	5.095831	2862
diarylsulfonesulfonamide	5.0956774	2863
selumetinib	5.0952897	2864
Aspartame	5.0951357	2865
DG-041	5.0950737	2866
LFM-A13	5.09506	2867
propylpyrazole	5.0947227	2868
fentiazac	5.09468	2869
nor-1-promazine	5.0944223	2870
pramipexole	5.094203	2871
Dipyridamole	5.0941467	2872
7b-cis	5.094048	2873
pantoprazole	5.094	2874
plerixafor	5.0936317	2875
rufloxacin	5.0934954	2876
Mitoxantrone HCl	5.093465	2877
harmine	5.093309	2878
secoisolariciresinol	5.0933027	2879
NCH-51	5.093261	2880
pyridine-2,4-dicarboxylic-acid	5.0931005	2881
imipenem	5.09264	2882
cefdinir	5.0925565	2883
GANT-58	5.0923786	2884
epirizole	5.0921545	2885
buflomedil	5.091999	2886
LEE011	5.0919576	2887
Trimethoprim	5.0919414	2888
asenapine	5.0918713	2889
Ivacaftor (VX-770)	5.0918026	2890
NBI-27914	5.091633	2891
pyrvinium-pamoate	5.0914183	2892
mafenide	5.0908422	2893
barasertib-HQPA	5.0898285	2894
Chromocarb	5.0897484	2895
clotrimazole	5.089621	2896
triamterene	5.0890417	2897
moexipril	5.088933	2898
alimemazine	5.088422	2899
Isovaleramide	5.0878773	2900
pranlukast	5.0876226	2901
mammea-a	5.0874963	2902
nonoxynol-9	5.08684	2903
Thalidomide	5.0865097	2904
Docosanol (Abreua)	5.0864654	2905

etoricoxib	5.0862966	2906
dorzolamide	5.08624	2907
Cytisine	5.0861773	2908
embelin	5.086136	2909
Clorsulon	5.086068	2910
dequalinium	5.0858326	2911
Tenofovir Disoproxil Fumarate	5.0857067	2912
mizolastine	5.085684	2913
Proflavine Hemisulfate	5.0856314	2914
Entinostat (MS-275,SNDX-275)	5.085434	2915
TCPOBOP	5.0852833	2916
zuclopenthixol	5.0851765	2917
naproxen	5.0850015	2918
GDC-0879	5.084861	2919
suprofen	5.084697	2920
Palbociclib (PD0332991) Isethionate	5.084562	2921
tyrphostin-AG-1295	5.0844493	2922
LY2157299	5.084442	2923
Allopurinol	5.084324	2924
brivanib	5.0843115	2925
damnacanthal	5.084034	2926
hydrocortisone-valerate	5.083593	2927
syringic-acid	5.083044	2928
Macitentan	5.082735	2929
Deferasirox	5.082348	2930
RITA	5.0820484	2931
Diclofenac	5.081987	2932
PTC124 (Ataluren)	5.081627	2933
Icotinib	5.0816126	2934
Apixaban	5.081609	2935
felbinac	5.081298	2936
APHA-compound-8	5.081056	2937
cefatrizine	5.081007	2938
vardenafil	5.080815	2939
mofezolac	5.080622	2940
NGB-2904	5.0804176	2941
Vardenafil	5.0795484	2942
Tenofovir	5.079418	2943
fenbendazole	5.0787096	2944
Cefdinir	5.078704	2945
Pasiniazid	5.0783024	2946
LY2784544	5.077471	2947
ochratoxin-a	5.0772057	2948
tyrphostin-47	5.077176	2949
puromycin	5.075825	2950
Methotrexate	5.075754	2951
rondual-kinase-inhibitor	5.075241	2952
Axitinib (AG 013736)	5.07506	2953
panobinostat	5.0744076	2954
Pimasertib (AS-703026)	5.0742197	2955
cediranib	5.074198	2956

Acetaminophen	5.073944	2957
trichloroethylene	5.0738688	2958
benazepril	5.073821	2959
rebamipide	5.073706	2960
wiskostatin	5.0735188	2961
4-iodo-6-phenylpyrimidine	5.07327	2962
Bumetanide	5.073175	2963
desmethylclomipramine	5.072462	2964
afatinib	5.0722675	2965
Trimipramine (maleate)	5.07168	2966
beta-CCP	5.0713835	2967
Trandolapril	5.071351	2968
eugenitol	5.0712953	2969
racecadotril	5.07111	2970
dexketoprofen	5.0709286	2971
Amiodarone HCl	5.0709	2972
benfluorex	5.070874	2973
linsitinib	5.0708303	2974
nTZDpa	5.0707273	2975
cefsulodin	5.0704365	2976
carmofur	5.070138	2977
Raltitrexed	5.0697517	2978
Iloperidone	5.0695434	2979
ethambutol	5.069334	2980
3,3'-diindolylmethane	5.068901	2981
Oxfendazole	5.0685263	2982
5-FP	5.068241	2983
CAL-101 (Idelalisib, GS-1101)	5.0679545	2984
BE-2254	5.0672665	2985
Imatinib Mesylate (STI571)	5.0671754	2986
TAK-438	5.0671186	2987
peucedanin	5.0670185	2988
antimycin-a	5.0669947	2989
Rosuvastatin Calcium	5.066795	2990
diaminoquinazoline	5.066498	2991
AKT-inhibitor-IV	5.06608	2992
methylene-blue	5.0660315	2993
pifithrin-alpha	5.0657144	2994
Piroxicam	5.065412	2995
fluprostenol	5.0653176	2996
FG-4592 (ASP1517)	5.065076	2997
chrysamine-g	5.064614	2998
reboxetine	5.063921	2999
ramipril	5.0638943	3000
enilconazole	5.0638185	3001
idelalisib	5.0632772	3002
Linsitinib	5.0632324	3003
α-Estradiol	5.0627627	3004
LE-300	5.0626116	3005
biotin	5.0625515	3006
ecopipam	5.062457	3007

erlotinib	5.061347	3008
canertinib	5.0613384	3009
pifithrin	5.0605907	3010
Mepiroxol	5.0604134	3011
Vortioxetine	5.0601225	3012
viloxazine	5.058976	3013
bisbenzimide	5.0588913	3014
m-chlorophenylbiguanide	5.058737	3015
rosmarinic-acid	5.0577183	3016
parbendazole	5.056513	3017
sulconazole	5.0564547	3018
ziprasidone	5.0563035	3019
nelfinavir	5.0560303	3020
ampiroxicam	5.055484	3021
sertraline	5.0551596	3022
Aprepitant	5.0550528	3023
mosapride	5.054678	3024
rucaparib	5.054017	3025
Lornoxicam	5.0535617	3026
Fulvestrant	5.053507	3027
propanoic-acid	5.0533504	3028
Chlorquinaldol	5.052704	3029
Tolvaptan	5.052457	3030
zaleplon	5.05208	3031
Afatinib (BIBW2992)	5.0511703	3032
olmesartan	5.0498476	3033
mepivacaine	5.0490074	3034
Vidofludimus	5.048833	3035
BIBR-1532	5.0482693	3036
SAR245409 (XL765)	5.0477943	3037
1-Hexadecanol	5.047533	3038
mesoridazine	5.047285	3039
nemonapride	5.0469694	3040
veliparib	5.0469103	3041
eicosatetraynoic-acid	5.0466976	3042
nobiletin	5.0447054	3043
emetine	5.0445833	3044
Lomefloxacin HCl	5.0442653	3045
Dasatinib (BMS-354825)	5.043035	3046
Saracatinib (AZD0530)	5.042959	3047
Phenytoin	5.042376	3048
NFKB-activation-inhibitor-II	5.0419817	3049
(+/-)-7-hydroxy-2-(N,N-di-n-propylamino)tetralin	5.0414677	3050
trioxsalen	5.0414567	3051
darinaparsin	5.040677	3052
farnesylthioacetic-acid	5.040621	3053
gaboxadol	5.0400424	3054
IDAM	5.0399804	3055
Lapatinib Ditosylate	5.03996	3056
gefitinib	5.0398617	3057
Tioconazole	5.0393586	3058

zatebradine	5.0392485	3059
gabazine	5.037756	3060
axitinib	5.037302	3061
p-aminophenethylspiperone	5.037279	3062
Sulfasalazine	5.03718	3063
actarit	5.0370293	3064
piperacetazine	5.0363617	3065
cyanoquinoline-11	5.035636	3066
Bazedoxifene acetate	5.0354004	3067
TAK-715	5.0353417	3068
Vandetanib (ZD6474)	5.0353403	3069
ioxaglic-acid	5.034754	3070
aminoindazole	5.0347433	3071
Istradefylline (KW-6002)	5.0343785	3072
flupirtine	5.033345	3073
7-hydroxy-PIPAT	5.0319395	3074
(+)-3-(1-propyl-piperidin-3-yl)-phenol	5.030031	3075
tangeritin	5.0299044	3076
Nevirapine	5.029256	3077
zolantidine	5.0288153	3078
Rilpivirine	5.028035	3079
amiodarone	5.027696	3080
darifenacin	5.0271053	3081
butamben	5.0264635	3082
Albendazole	5.026269	3083
Salirasib	5.025522	3084
tetraethylenepentamine	5.0251217	3085
ODQ	5.024902	3086
fananserin	5.024432	3087
tenatoprazole	5.0238667	3088
lavendustin-c	5.020563	3089
BI-2536	5.0204897	3090
indatraline	5.020365	3091
lavendustin-a	5.0187616	3092
viomycin	5.017145	3093
serdemetan	5.015568	3094
luzindole	5.014001	3095
obatoclax	5.0132103	3096
GSK2126458	5.012439	3097
Triclabendazole	5.010357	3098
flubendazole	5.0091887	3099
desloratadine	5.0089273	3100
Merck60	5.00706	3101
Mefenamic Acid	5.0050383	3102
pefloxacin	5.0040517	3103
caffeic-acid-phenethyl-ester	5.003372	3104
dichlorodiamine-platinum	5.0033393	3105
Esomeprazole Magnesium	5.0033026	3106
Azilsartan Medoxomil	5.0021744	3107
triclabendazole	5.002057	3108
cadmium-chloride	5.001959	3109

benzydamine	5.001246	3110
AKT-inhibitor-1-2	4.998198	3111
dihydrostreptomycin	4.997206	3112
cisplatin	4.996172	3113
pterostilbene	4.9960155	3114
Darifenacin HBr	4.995536	3115
Cariprazine	4.9947987	3116
GANT-61	4.9928417	3117
sarafloxacin	4.9921746	3118
Osimertinib (AZD9291)	4.9919534	3119
NF-449	4.9891825	3120
olvanil	4.9885073	3121
MK3102	4.9857645	3122
Nelfinavir	4.977195	3123
metergoline	4.9750547	3124
Chlorhexidine digluconate	4.971981	3125
Mebendazole	4.971431	3126
AG-14361	4.968335	3127
anacardic-acid	4.9663982	3128
RHO-kinase-inhibitor-III[rockout]	4.964534	3129
irilin-a	4.9635525	3130
Omeprazole	4.963001	3131
Flubendazole	4.9594584	3132
Albendazole Oxide	4.9576263	3133
varenicline	4.9201956	3134
iriflophenone	4.91286	3135
ioversol	4.8981714	3136
mebhydrolin	4.881054	3137
