# Supplementary material for: Improving generalizability of drug–target binding prediction by pre-trained multi-view molecular representations
Source: Bioinformatics. 2025 Jan 7;41(1):btaf002. doi: 10.1093/bioinformatics/btaf002 (PMC11751634; doi:10.1093/bioinformatics/btaf002)
Supplement: btaf002_Supplementary_Data [file btaf002_supplementary_data.zip › 2f93e_Supplementary File 1.pdf]

Supplementary Materials for 'Improving Generalizability of Drug-Target Binding Prediction by Pre-trained Multi-view Molecular Representations'

Xike Ouyang[1,†], Yannuo Feng[1,†], Chen Cui[2], Yunhe Li[1], Li Zhang[2,*], Han Wang[1,*]

1 School of Information Science and Technology, Institute of Computational Biology, Northeast Normal University, Changchun 130117, Jilin, China

2 School of Computer Science and Engineering, Changchun University of Technology, Changchun 130051, Jilin, China

This pdf file includes:

- Supplementary Methods and Materials
- Supplementary Results
- Supplementary Tables S1-S4
- Supplementary Figures S1-S5

*Corresponding authors. E-mail: wangh101@nenu.edu.cn; E-mail: lizhang@ccut.edu.cn

† Xike Ouyang and Yannuo Feng contributed equally to this work and as first authors.

Supplementary materials

1 Methods and Materials

1.1 Training Setting

In the training phase, for DTI and DTA prediction tasks, we use the cross-entropy loss function and the MSE loss function to calculate the loss respectively. Both use the Adam optimizer to optimize parameters, and use the ReduceLROnPlateau function to dynamically adjust the learning rate.

We try to design different learning rates (lr), batch sizes and epochs for each dataset and use grid search to determine the best parameters. We tried {1e-1, 1e-2, 1e-3, 1e-4, 5e-2, 5e-3, 5e-4} as learning rates, and finally selected {1e-3, 1e-3, 5e-4, 1e-3} for training and testing on the Davis, PDBbind, TDC-DG, and BindingDB datasets respectively. For the epochs of Davis, PDBbind, TDC-DG, and BinindDB datasets, we set them to 1000, 200, 200, and 500 respectively. Similarly, different datasets also have different batch sizes. For batch size, we tried {32, 64, 128, 256}. Finally, {256, 128, 64, 64} were selected as the batch sizes for training and testing on the Davis, PDBbind, TDC-DG, and BindingDB datasets respectively. All hyperparameters are shown in Table S1. All experiments were run on Linux OS with a NVIDIA GeForce RTX V100 GPU and a 32 vCPU Intel Xeon Processor (Skylake, IBRS).

Table S1. Summary of parameter settings for PMMR.

Hyperparameter	Setting
Number of linear attention heads	10
Number of Transformer heads	4
Num of Transformer layer	1
Num of GCN layers	3
Number of headers in the decoder	4
Num of decoder layers	1
Dropout rate	0.2
Optimizer	Adam

1.2 Metrics

MSE is a method of evaluating the error between the true value and the predicted value. It is defined as follows:

$$MSE = \frac{1}{n}\sum_{i=1}^{n}(P_i - Y_i)^2, \quad (1)$$

Where P_i is the predicted value, and Y_i is the real value.

RMSE (Root Mean Square Error) quantifies the average magnitude of errors between predicted and actual values, commonly used to assess the accuracy of predictive models. It is defined as follows:

$$RMSE = \sqrt{\frac{1}{n}\sum_{i=1}^{n}(P_i - Y_i)^2}, \quad (2)$$

CI is used to measure the probability of agreement between the predicted value and the real value.

$$CI = \frac{1}{Z}\sum_{\delta_x > \delta_y} h(b_x - b_y), \quad (3)$$

Where b_x is the prediction value of the model for the larger affinity δ_x, b_y is the prediction value of the model for the smaller affinity δ_y. Z is a normalization constant, and $h(k)$ is the step function:

$$h(k) = \begin{cases} 1, & k > 0 \\ 0.5, & k = 0. \\ 0, & k < 0 \end{cases} \quad (4)$$

$\mathrm{r_m^2}$ is a measure used to evaluate the generalization ability of the model. When the $\mathrm{r_m^2} > 0.5$ is determined to be an acceptable model.

$$r_m^2 = r^2 * \left(1 - \sqrt{r^2 - r_0^2}\right), \quad (5)$$

Where r^2 and r_0^2 are the squared correlation coefficients with and without intercept, respectively.

Pearson correlation coefficient measures the strength and direction of a linear relationship between two variables, ranging from -1 (perfect negative correlation) to 1 (perfect positive correlation).

$$Pearson = \frac{\sum_{i=1}^{n}(x_i - \bar{x})(y_i - \bar{y})}{\sqrt{\sum_{i=1}^{n}(x_i - \bar{x})^2}\sqrt{\sum_{i=1}^{n}(y_i - \bar{y})^2}}, \quad (6)$$

Where x_i and y_i are the individual data points, $\bar{x}$ and $\bar{y}$ represent their respective means.

Spearman is a non-parametric statistical method used to measure the correlation between two variables, based on their ranks rather than their raw values. It is defined as follows:

$$Spearman = 1 - \frac{6\sum_{i=1}^{n} d_i^2}{n(n^2-1)}, \quad (7)$$

Where d_i is the difference between two ranks in the predicted values and labels.

MAE (Mean Absolute Error) is a metric commonly used to evaluate the accuracy of a predictive model. It measures the average absolute difference between the predicted values and the actual values as follows:

$$MAE = \frac{1}{n}\sum_{i=1}^{n} |y_i - p_i|. \quad (8)$$

AUC (Area under Curve): The area under the ROC curve, between 0.1 and 1. AUC can be used as a numerical value to intuitively evaluate the quality of a classifier. The larger the value, the better. The specific calculation formula is as follows:

$$AUC = \sum_{i=1}^{n-1} \frac{1}{2}(TPR_i + TPR_{i+1}) \cdot (FPR_{i+1} - FPR_i), \quad (9)$$

Where TPR_i and TPR_{i+1} are the true positive rates at adjacent thresholds, and FPR_i and FPR_{i+1} are the false positive rates at adjacent thresholds. The summation is performed over all thresholds from 1 to $n-1$, where n is the total numbers of data.

AUPR (Area Under the Precision-Recall curve) quantifies the performance of a binary classification model by measuring the area under the curve formed by plotting precision against recall. It is defined as follows:

$$AUPR = \sum_{i=1}^{n-1} \frac{1}{2}(P_i + P_{i+1}) \cdot (R_{i+1} - R_i), \quad (10)$$

Where P_i and P_{i+1} are the precision values at adjacent thresholds, R_{i+1} and R_i are the recall values at adjacent thresholds, and the summation is performed over all thresholds from 1 to $n-1$, where n is the total numbers of data.

2 Results

2.1 Evaluation of computational efficiency

To comprehensively evaluate the performance of PMMR, we compared its training time, testing time, model size, and GPU memory usage with those of other baseline methods on the PDBbind dataset.

As shown in Table S2, when training and testing on the PDBbind dataset, the model's training and testing speeds per epoch are slower than those of other baseline models, and its GPU memory usage is significantly higher. The reason for this result is that the embeddings obtained from

pre-training are too large. For instance, the protein feature dimension generated by ESM-2 is $n \times 480$, while the drug feature dimension generated by ChemBerta-2 is $n \times 384$. At the same time, since the lengths of proteins and drugs are not uniform, the input length for each batch needs to be standardized. Our approach is to use the maximum length as the unified standard, padding data shorter than this length. This increases the data dimensions, computational complexity, and consequently, the training and testing times of the model. In contrast, previous methods, such as GraphDTA(Nguyen, et al., 2021) and DeepGLSTM(Mukherjee, et al., 2022), use a fixed length for proteins and drugs. If the length is shorter than the fixed value, it is padded; if it is longer, it is truncated. While this approach reduces the feature dimensions and improves computational efficiency, truncation may lead to the loss of key features, thereby affecting the model's predictive performance. To address this, we opted for padding only, without truncation, to preserve complete protein and drug information.

Table S2. PMMR and the baseline model in training speed, test speed, model size, and GPU memory usage.

Model	Training speed	Testing speed	Model size	GPU memory usage
PMMR	2m	2s	8.75M	30796M
GraphDTA	2s	0.02s	4.95M	2734M
DGDTA	2s	0.03s	18.73M	6716M
DeepGLSTM	6s	0.05s	131.92M	3630M

2.2 Ablation study

In order to verify the effect of the Transformer in the PMMR model, we conducted ablation experiments. As shown in Table S3, on both the Davis and PDBbind datasets, the method of fine-tuning pre-trained features using the Transformer significantly outperforms the method that does not use the Transformer and relies only on MLP. This demonstrates that fine-tuning the pre-trained features with the Transformer helps improve the predictive performance of the model.

Table S3. The effect of fine-tuning pre-trained features using different methods.

Dataset	Approach	Pearson	Spearman
Davis	Transformer	0.908	0.737
	MLP	0.864	0.709
PDBBind	Transformer	0.816	0.827
	MLP	0.786	0.788

2.3 Classification performance evaluation

Based on the BindingDB data set, we evaluated the classification performance. Figure S1 shows the ROC curve after five random seed initializations. It can be seen that the results of the five random tests are very close, indicating that the model is very stable in the classification test.

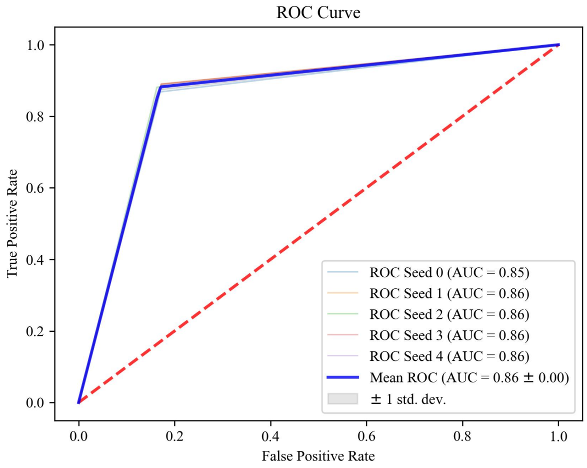

Figure S1. Average ROC curve of five random tests on the BindingDB dataset.

2.4 The effects of various PLMs

We explored several choices for using protein language models (PLMs) to generate sequence features, including ESM-2 (PMMR) (Lin, et al., 2023) , ProtTrans (PMMR-ProtTrans) (Elnaggar, et al., 2022), TAPE (PMMR-TAPE) (Rao, et al., 2019). As shown in Figure S2, we show the Spearman values of the three pre-training methods on the three benchmark affinity datasets. Judging from the results, using ESM-2 as a pre-training method achieved the best performance in all test results.

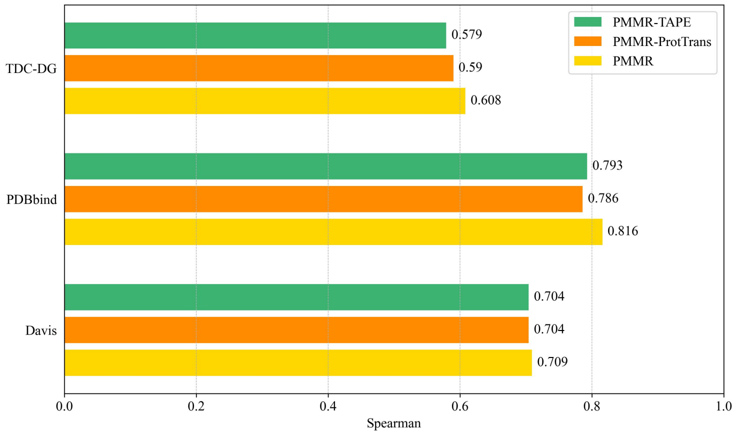

Figure S2. The evaluation of pre-trained features generated by different protein language models (PLMs) on three benchmark affinity datasets.

2.5 The effects of different views

In order to verify whether the global features extracted using Transformer and the global features extracted by GCN can complement each other, we designed two model variants: PMMR-Transformer and PMMR-GCN. And compared the Spearman value with PMMR on three benchmark affinity datasets. As shown in Figure S3, PMMR achieved the best sorting performance on TDC-DG, while on the Davis and PDBbind datasets, the results were similar to PMMR-GCN and PMMR-Transformer.

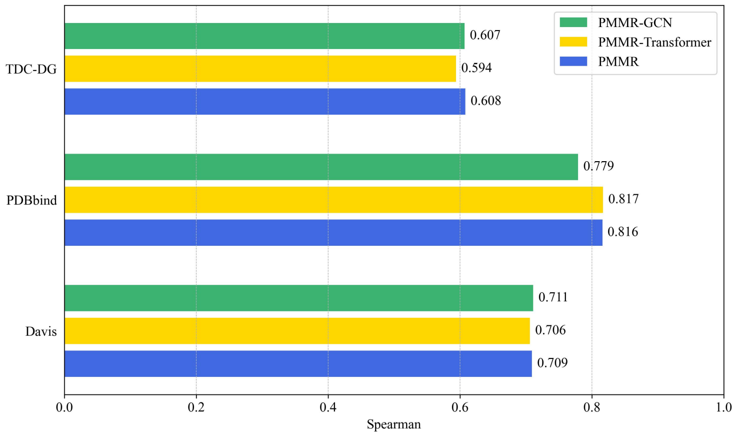

Figure S3. The impact of different view features of drugs on model performance was evaluated across three benchmark affinity datasets.

2.6 The difference between the predicted value and the experimental value

We present scatter plots of experimental affinities and PMMR predicted affinities on three benchmark affinity test sets in Figure S4. For each point in the graphs, the x-axis refers to its experimental affinity value and the y-axis refers to its predicted affinity value, the vertical distance $|\Delta y|$ from each point to y = x represents the discrepancy between its predicted value and the experimental value.

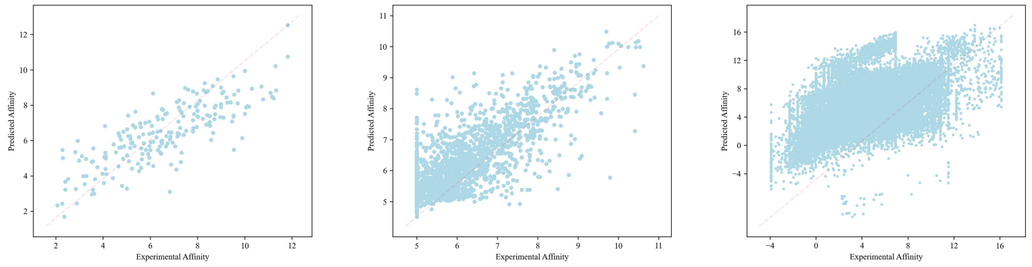

Figure S4. The differences in predicted and experimentally measured affinity values on three benchmark affinity datasets. From left to right are the scatter plots on the PDBbind, Davis and TDC-DG test sets.

2.7 Cross-dataset Experimentation

As shown in Table S4, we selected labels with affinity K_d values in PDBbind and used them as the test set. We then used the model trained on the Davis training set to evaluate its performance on the PDBbind test set. The obtained Pearson and Spearman correlation values are 0.129 and 0.141, respectively. However, when the model trained on the Davis training set was used to test on the PDBbind test set, and the model trained on the PDBbind training set was used to test on the Davis test set, the results were not satisfactory.

We analyzed the possible reasons for this situation based on the affinity distribution. As shown in the upper part of Figure S5, the affinity values in the Davis training and test sets range from 5 to 11, with most values concentrated around 5. In contrast, the affinity values in the PDBbind training and test sets range from 0 to 14. The training set values are mostly concentrated around 6, forming a normal distribution, while the test set values are more evenly distributed. When using the Davis training set, the model tends to be biased towards the majority class due to the highly concentrated label distribution. This bias negatively impacts both prediction accuracy and generalization performance, resulting in suboptimal performance on new data with different distributions. Similarly, training on the PDBbind training set and testing on the Davis test set can also affect the model's performance due to the inconsistent label distributions between the two sets.

In the future, we will explore the nature of drug-target interactions to enable the model to learn this key feature and further enhance its generalization performance.

Table S4. Cross-dataset training and testing performance.

Train	Test	Pearson	Spearman
Davis(K_d)	PDBbind(K_d)	0.129	0.141
Davis	PDBbind	0.248	0.231
PDBbind	Davis	0.083	0.103

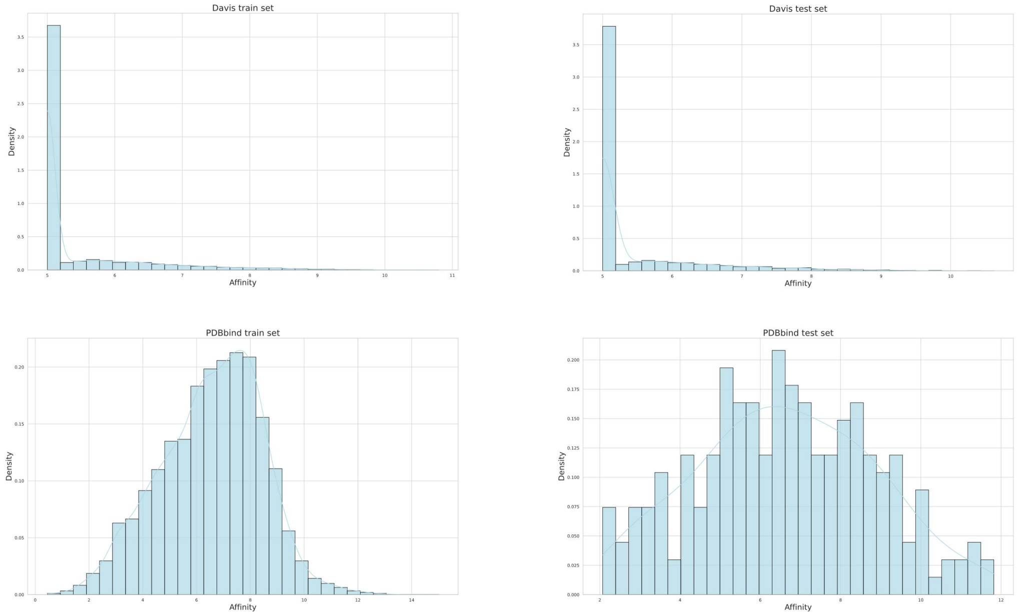

Figure S5. The frequency histogram of binding affinity in Davis and PDBbind dataset.

References

1 Elnaggar, A., *et al.* ProtTrans: Toward Understanding the Language of Life Through Self-Supervised Learning. *IEEE Trans Pattern Anal Mach Intell* 2022;44(10):7112-7127.

2 Lin, Z., *et al.* Evolutionary-scale prediction of atomic-level protein structure with a language model. *Science* 2023;379(6637):1123-1130.

3 Mukherjee, S., Ghosh, M. and Basuchowdhuri, P. DeepGLSTM: deep graph convolutional network and LSTM based approach for predicting drug-target binding affinity. In, *Proceedings of the 2022 SIAM international conference on data mining (SDM)*. SIAM; 2022. p. 729-737.

4 Nguyen, T., *et al.* GraphDTA: predicting drug-target binding affinity with graph neural networks. *Bioinformatics* 2021;37(8):1140-1147.

5 Rao, R., *et al.* Evaluating protein transfer learning with TAPE. *Advances in neural information processing systems* 2019;32.
