# Supplementary figures and images for: Improving generalizability of drug–target binding prediction by pre-trained multi-view molecular representations
Source: Bioinformatics. 2025 Jan 7;41(1):btaf002. doi: 10.1093/bioinformatics/btaf002 (PMC11751634; doi:10.1093/bioinformatics/btaf002)

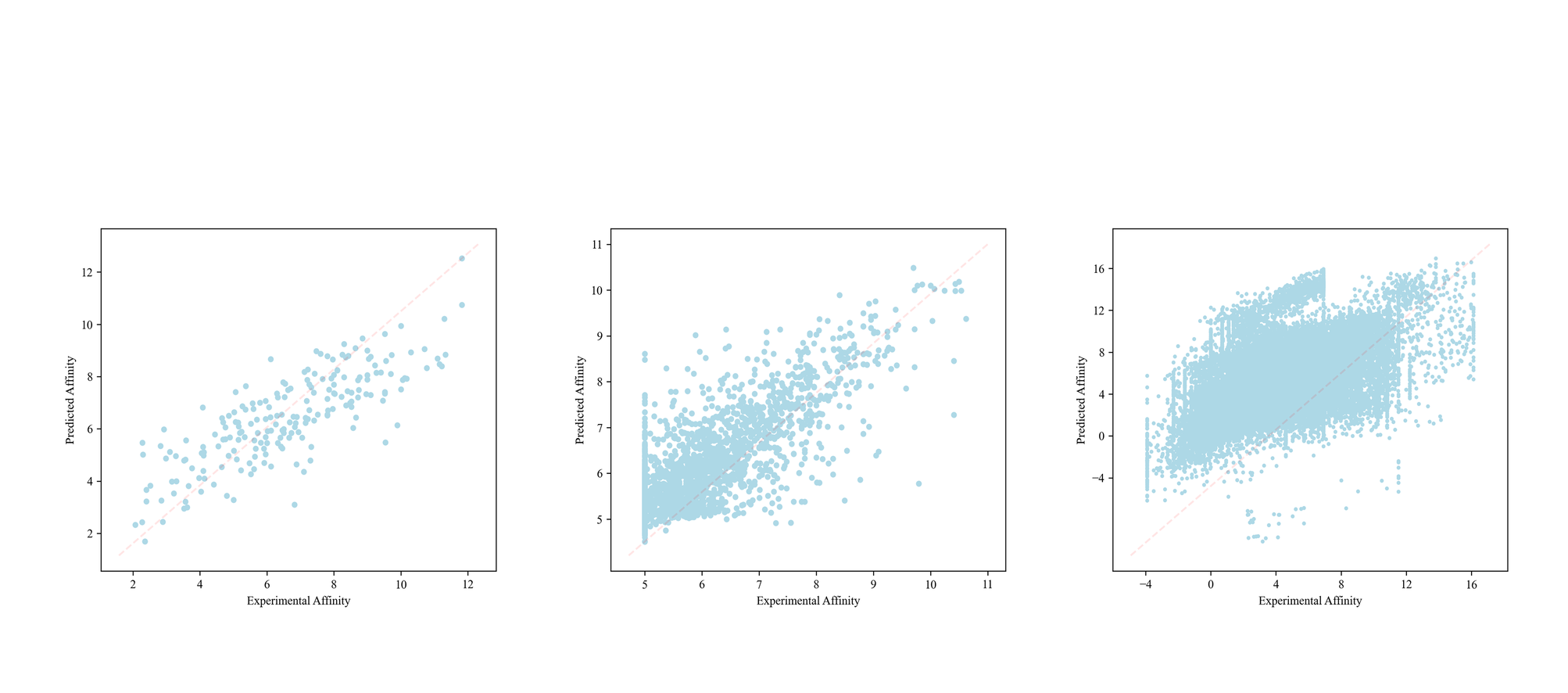

Supplement: btaf002_Supplementary_Data [file btaf002_supplementary_data.zip › 57a49_Fgiure S4.tif]

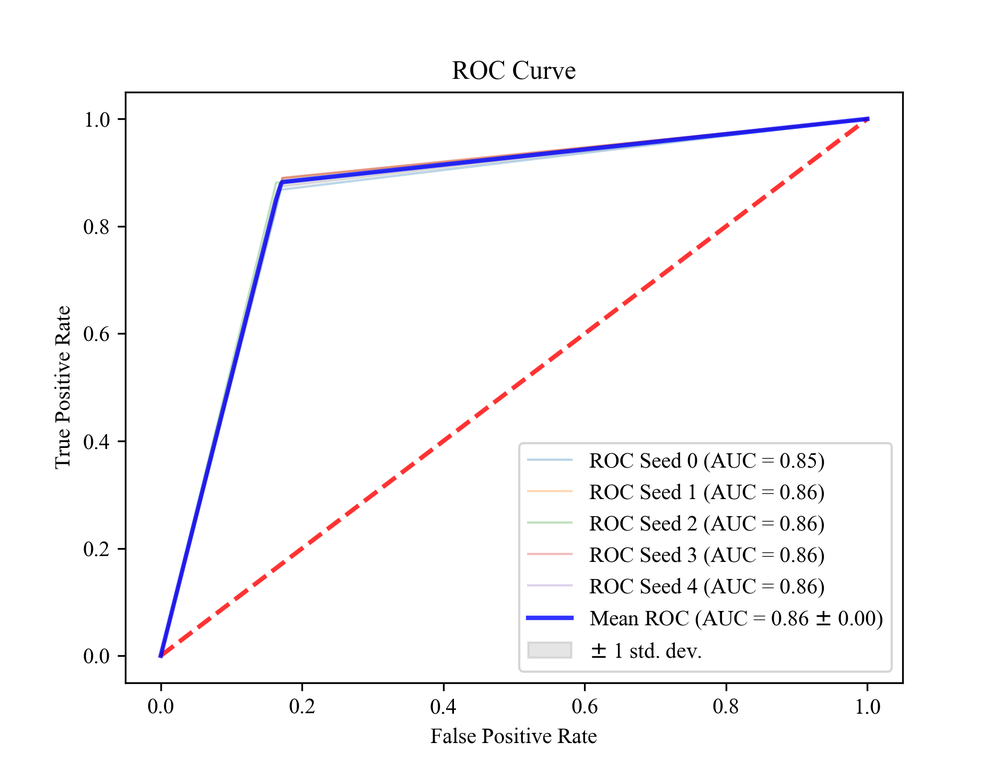

Supplement: btaf002_Supplementary_Data [file btaf002_supplementary_data.zip › 2dbe9_Figure S1.tif]

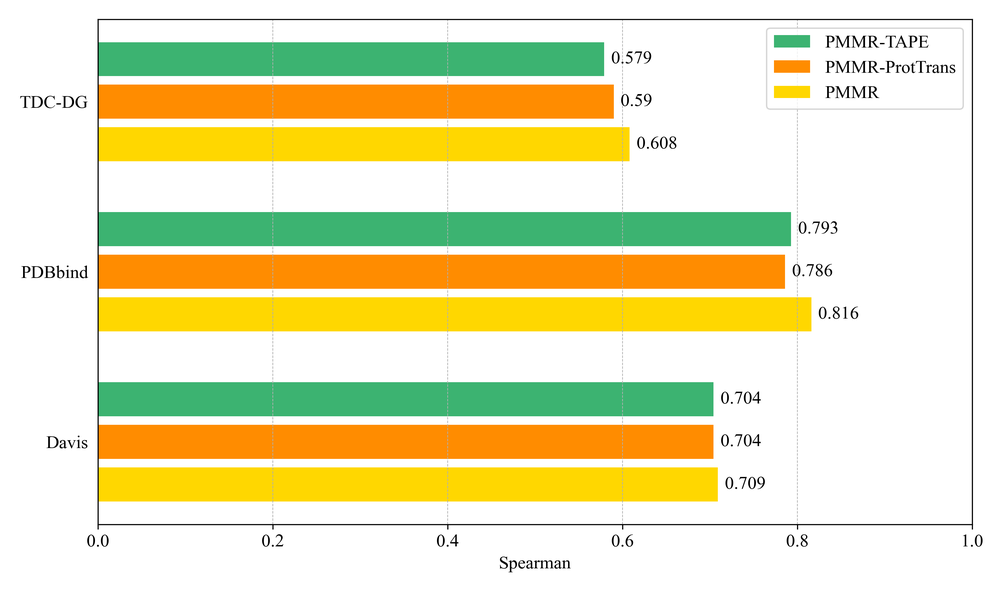

Supplement: btaf002_Supplementary_Data [file btaf002_supplementary_data.zip › 813ac_Figure S2.tif]

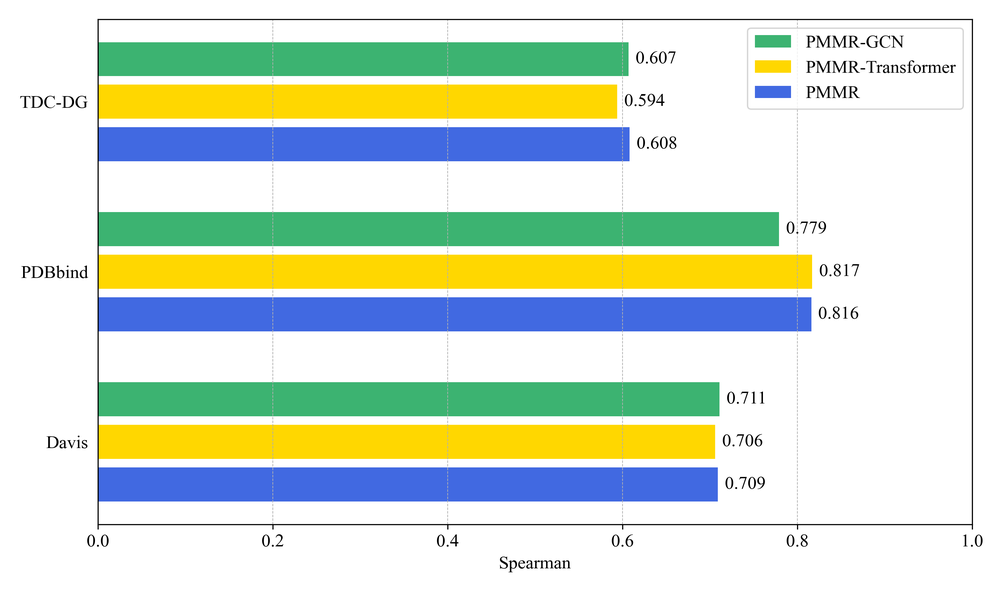

Supplement: btaf002_Supplementary_Data [file btaf002_supplementary_data.zip › b7cf4_Figure S3.tif]

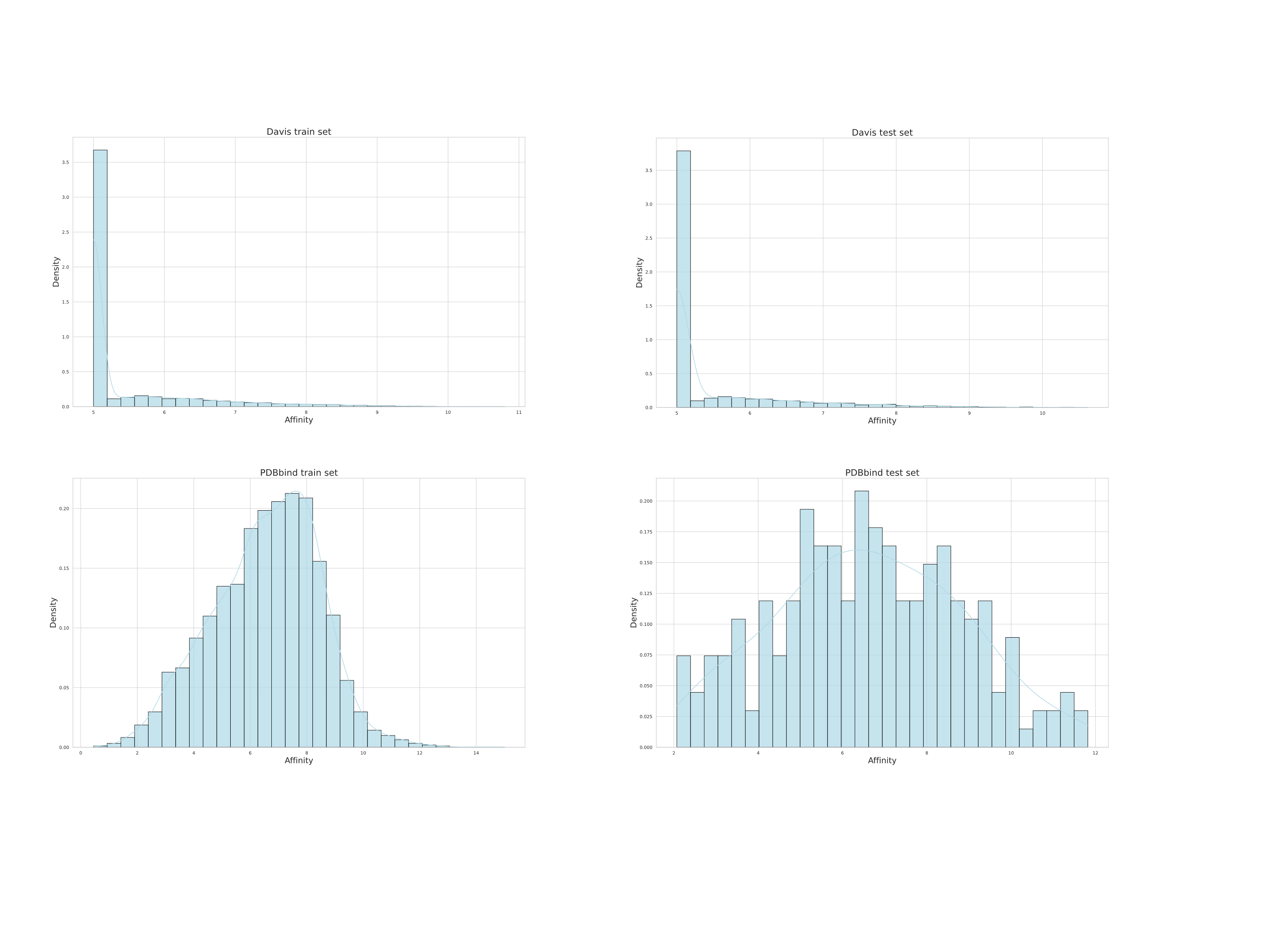

Supplement: btaf002_Supplementary_Data [file btaf002_supplementary_data.zip › 602ff_Figure S5.tif]
